# LPPtiger software for lipidome-specific prediction and identification of oxidized phospholipids from LC-MS datasets

**DOI:** 10.1038/s41598-017-15363-z

**Published:** 2017-11-09

**Authors:** Zhixu Ni, Georgia Angelidou, Ralf Hoffmann, Maria Fedorova

**Affiliations:** 1 Institute of Bioanalytical Chemistry, Faculty of Chemistry and Mineralogy, Universität Leipzig, Deutscher Platz 5, 04103 Leipzig, Germany; 2 Center for Biotechnology and Biomedicine, Universität Leipzig, Deutscher Platz 5, 04103 Leipzig, Germany

## Abstract

Oxidized phospholipids (oxPLs) have been recently recognized as important mediators of various and often controversial cellular functions and stress responses. Due to the low concentrations *in vivo*, oxPL detection is mostly performed by targeted mass spectrometry. Although significantly improving the sensitivity, this approach does not provide a comprehensive view on oxPLs required for understanding oxPL functional activities. While capable of providing information on the diversity of oxPLs, the main challenge of untargeted lipidomics is the absence of bioinformatics tools to support high-throughput identification of previously unconsidered, oxidized lipids. Here, we present LPPtiger, an open-source software tool for oxPL identification from data-dependent LC-MS datasets. LPPtiger combines three unique algorithms to predict oxidized lipidome, generate oxPL spectra libraries, and identify oxPLs from tandem MS data using parallel processing and a multi-scoring identification workflow.

## Introduction

Over last two decades, the predominant view of lipid peroxidation products (LPPs) underwent a significant paradigm shift – LPPs, previously seen as a toxic byproduct of free radical reactions, nowadays is recognized as an important mediator of various cellular responses and plays a significant role in organism redox balance^1^. Significant success in deciphering the role of free fatty acid (FFA)-derived LPPs, especially iso- and neuroprostanes, established them as biomarkers of inflammatory pathways^2^. Furthermore, the discovery of LPP synthesis enzymes, such as cyclooxygenases and lipoxygenases, allowed for the designing of widely used pharmacological intervention strategies^3^.

More recently, potential biological activity studies were translated from FFA-derived LPPs to oxidized phospholipids (oxPLs). PL-bound LPPs have been demonstrated to play a significant role in platelet differentiation^4^, induction of ferroptosis signaling^5^, as well as an anti-inflammatory mediator in the context of atherosclerosis^6^. Despite a relatively limited amount of available data, it becomes clear that the structure of LPPs is one of the main determinants of their diverse biological activities, including pro-inflammatory and death signaling as well as anti-inflammatory and pro-survival effects. Systems wide profiling and identification of large number of PL-bound LPPs in biological samples are required to understand structure-functional relationships determining biological activities. Until now, most of the research in biological systems involved targeted MS strategies due to the low abundance on endogenous LPPs in complex natural lipidomes. Availability of state-of-the-art MS instruments characterized by improved sensitivity and high dynamic range of detection, combined with optimized analytical workflows, provides possibility for untargeted LPP profiling. However, a bioinformatics solution for high-throughput identification of highly complex sets of possible LPPs remains the main challenge in systems wide LPP profiling.

## Results and Discussion

**Figure 1:**
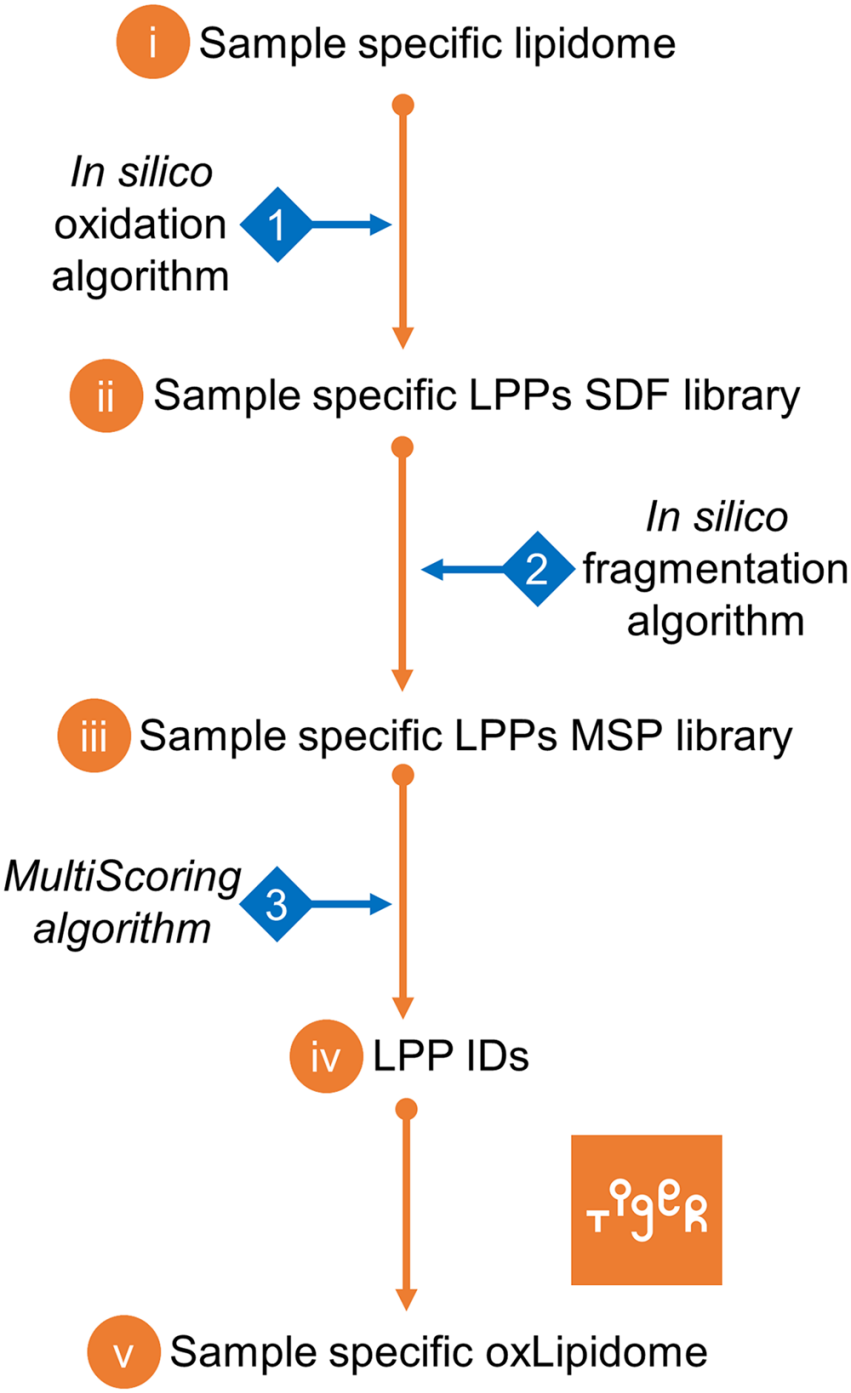
LPPtiger workflow. The LPPtiger uses three unique algorithms: *in silico* oxidation (1), *in silico* fragmentation (2), and multi-score-based identification (3). Additionally, LPPtiger utilizes sample-specific native lipidome (*i*) to predict PL-bound LPPs (*ii*) from which a simulated tandem mass spectra library (*iii*) is generated and used for LPP identification (*iv* and *v*).

Here we present LPPtiger, a new open-source software tool processing LC-MS/MS data dependent acquisition (DDA) datasets, which is capable of predicting sample-specific oxidized lipidome and used it for PL-LPP identification (Fig. 1). LPPtiger workflow relies on the sample-specific PL lipidome with a defined, discrete fatty acid composition. Considering the number, type, and positions of modifications, as well as combinations of these three factors, the estimated number of potential LPPs derived from the initial lipidome is several orders of magnitudes larger than the number of native lipids. The exact FA composition of PLs will define the ensuing variety of LPP formed. For instance, PC(36:4) may correspond to at least two distinct lipids – PC(18:2/18:2) and PC(16:0/20:4). Oxidation of PC(18:2/18:2) results in 17 different LPP structures, while at least 104 LPPs can be formed from its isomer PC(16:0/20:4). Thus, based on the knowledge of original lipidome composition, an estimation of LPP variety can largely reduce analysis time and increase specificity of LPP identification.

**Figure 2:**
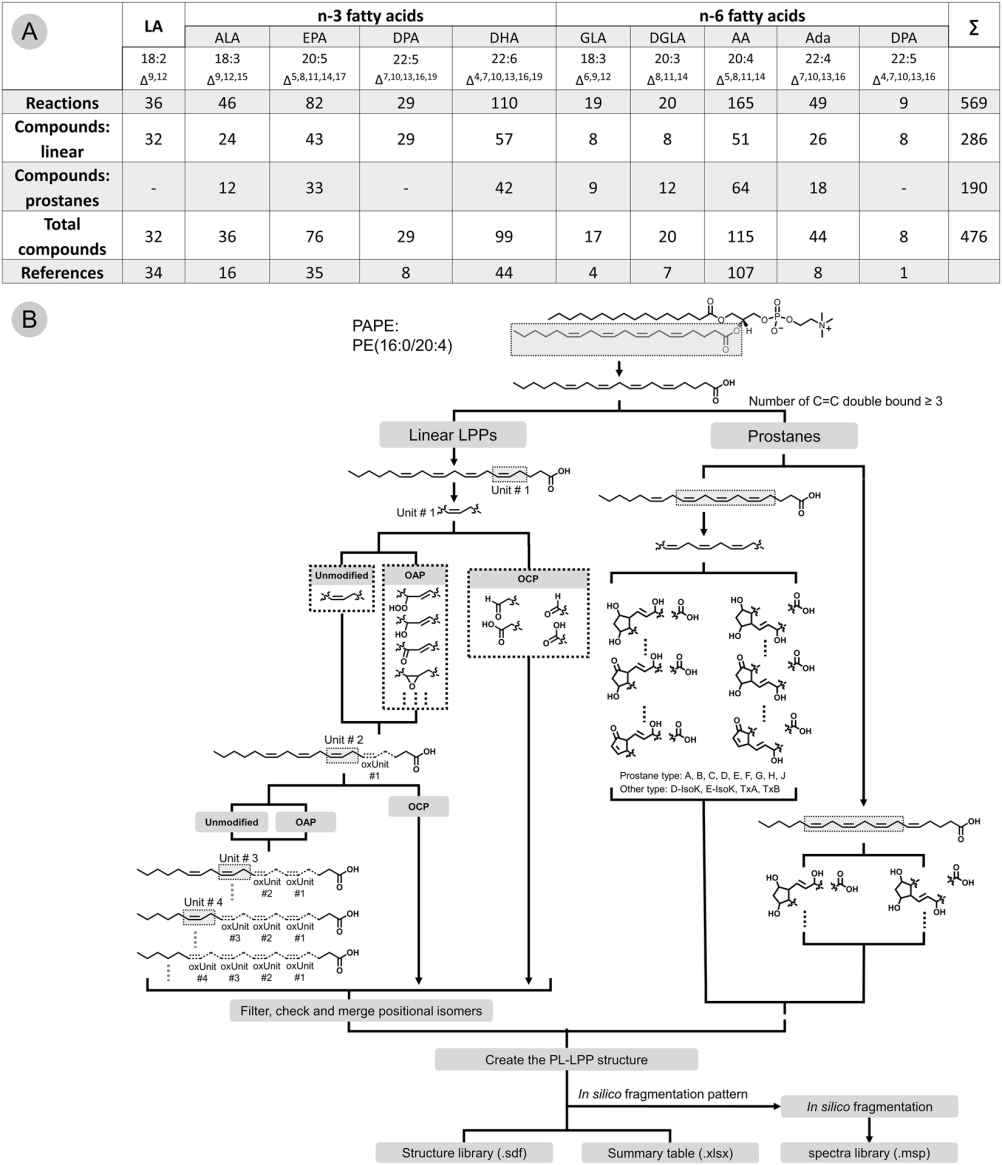
Summary of meta-study data integration (**A**) and (**B**) formulation of an *in silico* oxidation algorithm. (**A**) – Summary of LPP networks reconstructed for the ten most abundant PUFA. (**B**) – Schematic representation of an *in silico* oxidation algorithm used for prediction of oxygen addition (OAP), oxidative cleavage (OCP) and prostane-containing LPPs exemplified on PC(16:0/20:4) lipid.

Based on a comprehensive meta-study including over 170 publications focusing on the enzymatic and non-enzymatic LPPs production (Fig. 2A, Supplementary Table 1), networks of enzymatic and free-radical-driven oxidative reactions were reconstructed for the ten most abundant PUFAs (18:2, 18:3 *n-3*, 18:3 *n-6*, 20:3 *n-6*, 20:4 *n-6*, 20:5 *n-3*, 22:4 *n-6*, 22:5 *n-3*, 22:5 *n-6*, and 22:6 *n-3*). CellDesigner-reconstructed networks are available as SBML files (Supplementary Files 1–10) and images at https://bitbucket.org/SysMedOs/filesrepository. Using this information, knowledge on the oxidation mechanisms for the bis-allylic positions in PUFA and the rearrangement of the neighboring double bonds was summarized and translated into *in silico* oxidation algorithms. Each double bond and one of the neighboring (bis-)allylic positions was treated as a core unit of the oxidation process, and possible modifications of the defined core unit were predicted by enumeration methods (Fig. 2B). The overall theoretically possible LPPs can be predicted by applying all possible modifications (introduction of keto, hydroxy, hydroperoxy, epoxy groups) to all double bond units in a certain PUFA acyl residue. For cyclic LPPs (e.g., prostanes), a three-double-bond core unit was used to construct the essential ring structures. Provided LPP networks included only oxygen addition products (OAP). For prediction of truncated oxidation cleavage products (OCP), the majority of which are formed via the Hock cleavage mechanism, each double bond position was treated as a potential point of truncation^7–11^. Predicted OCP were further populated with oxidation-derived functional groups (e.g., hydroxy and keto) based on the number of remaining double bonds (Fig. 2B).

Reconstructed networks of PUFAs oxidation used to design *in silico* oxidation algorithm included both enzymatic and non-enzymatic oxidation routes. Although it is possible to predict all combinations of modifications for all double bond units in selected PUFA, limits for the number and type of overall modifications produced under physiological conditions were introduced based on the literature survey, and defined via three levels (most common mild oxidation, pathway dependent, and less often reported oxidation routes; Supplementary Figure S1). These theoretical oxidation rules were implemented as *in silico* oxidation algorithms capable of predicting LPPs from the list of original PLs within the LPPtiger software. Using sample-specific lipidome as .xlsx file input, LPPtiger can predict sample-specific oxLipidome and store it as an .sdf library.

**Figure 3:**
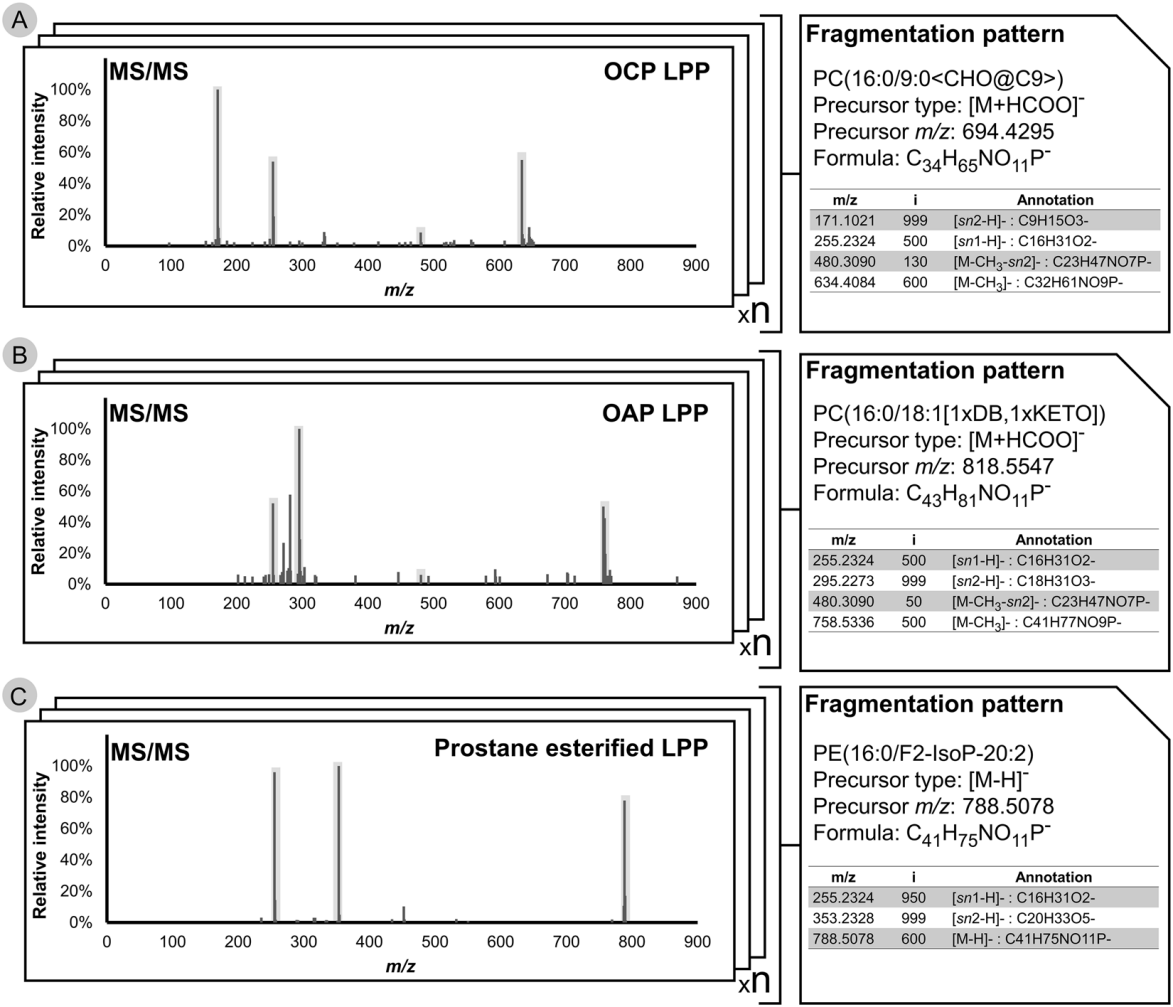
Illustration of LPP fragmentation patterns used to generate *in silico* spectra libraries. Based on a list of manually-assigned spectra of OCP (**A**), OAP (**B**), and prostane-esterified LPPs (**C**), the main structure-related signals were marked and the corresponding relative intensities were indexed for each individual spectrum. The type and relative intensities of structure-related signals from multiple assigned spectra of the same LPP type were summarized and averaged to generate a compound-specific fragmentation profile.

For a sample-specific .sdf library of potential oxidized PLs, LPPtiger utilizes an *in silico* fragmentation algorithm, aiming to simulate collision induced dissociation (CID) tandem mass spectra for each predicted LPP. MS and MS/MS behavior of native PLs have been intensively characterized by many research groups over the last decade^12–14^. However, far less information on ionization and fragmentation mechanisms of PL-bound LPPs is currently available. Moreover, general mechanisms of the MS behavior of LPPs cannot simply be extrapolated from corresponding PLs. The fragmentation mechanism of PL-bound LPPs, although somewhat similar to PLs, has its own specifics. A review of available literature^7,9–11,15,16^ and intensive MS and MS/MS studies (manual assignment of more than 300 in-house acquired MS/MS spectra) of *in vitro*-oxidized PL standards revealed several fragmentation patterns that have been defined for different types of PL-bound LPPs (Fig. 3; Supplementary Table 2). For instance, it should be noticed that truncated PC-bound LPPs will form different adducts upon the negative ionization mode in the presence of formate ions. Thus, carbonylated species will form prominent formate anion adducts similar to native PCs, whereas OCP, carrying terminal carboxylic moiety, will form deprotonated ions. Upon CID fragment ions and neutral losses of head group related structures, *sn*-1 and *sn*-2 residues are the main signals populating tandem mass spectra. Certain intensity distributions can be summarized for these ions based on the tandem mass spectra of LPP standards (Supplementary Table 2). Additionally, for some modifications, specific neutral losses can be detected (e.g. water loss from hydroxylated FA chains or hydrogen peroxide from lipid peroxides), while other modifications do not display any specific fragment ions (e.g. epoxy and keto groups). Modification specific fragments/neutral losses can partially assist prediction of MS/MS spectra for isomeric oxPLs. Thus, *in silico* fragmentation algorithm generated MS/MS spectra containing main (fragment ions and neutral losses of head group related structures, *sn*-1 and *sn*-2 residues) as well as modification specific (fingerprint predictions) fragment ions and neutral losses (Supplementary Figure S2). Together with the predicted LPP structures, these fragmentation features were used for the generation of an *in silico*-fragmented spectra library which can be used for the identification of LPPs by spectra matching approaches. Thus, LPPtiger utilized *in silico* fragmentation algorithms to generate an .msp library for all predicted LPPs. In the current version of LPPtiger, fingerprint prediction includes neutral losses specific for hydroxy, hydroperoxy and carboxy modified FA residues. However, upcoming LPPtiger versions will be able to predict elevated energy CID and MS^3^ spectra, necessary to assign positional isomers.

**Figure 4:**
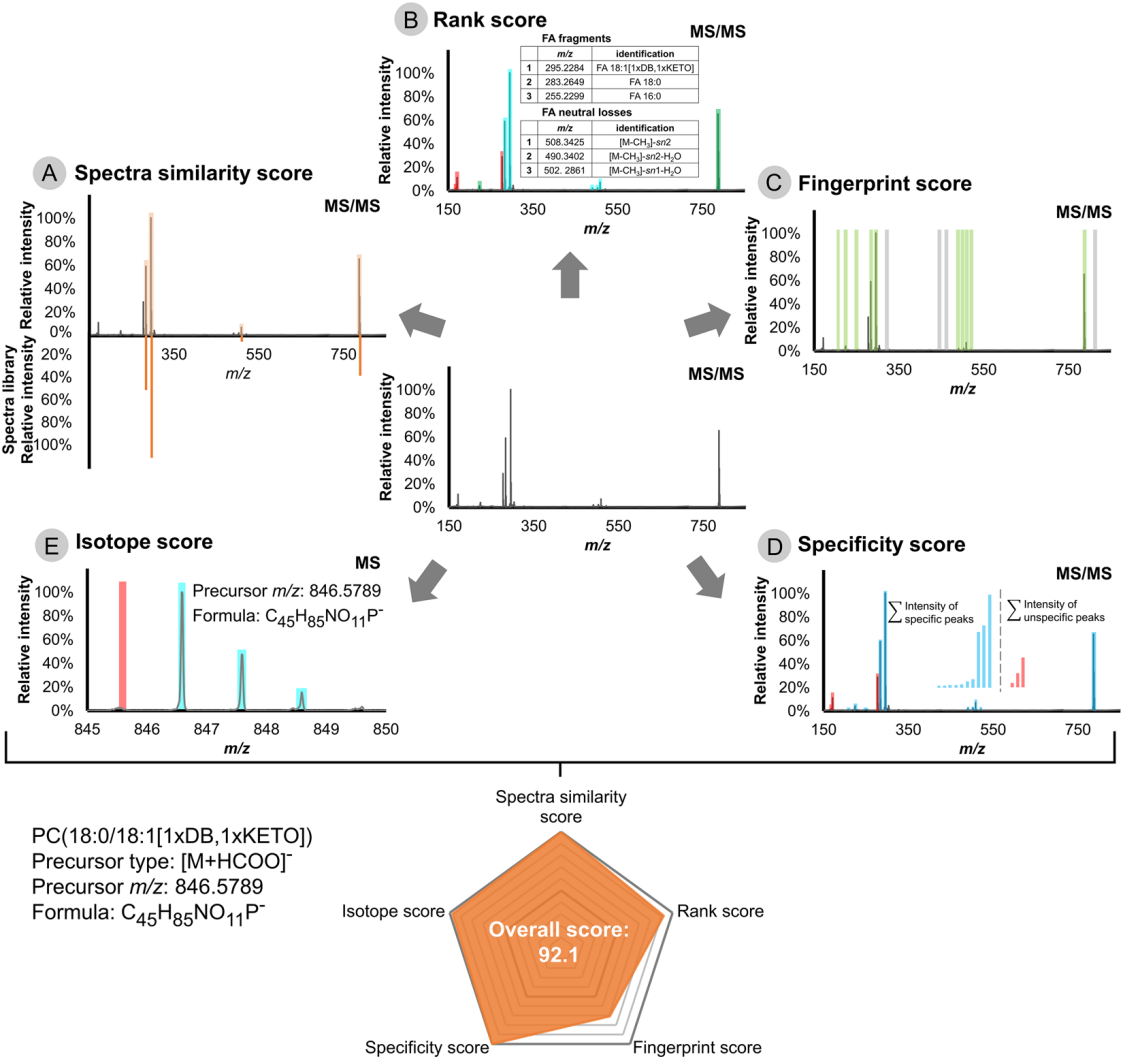
Overview of five scoring algorithms used by LPPtiger identification workflow. LPPtiger combines the reverse dot-product Spectra Similarity Score (**A**), intensity based Rank Score for bottom-up identification using a white list of possible oxFA and PL head group combinations (**B**), intensity-independent Fingerprint Score that considers oxPL-specific fragments and neutral losses (**C**), Specificity Score to evaluate the ratio between LPP-specific and unspecific fragments (**D**) and Isotope Score to evaluate isotope pattern fit (**E**). Five individual scores are averaged to derive a final LPPtiger Score for each identified PL-bound LPP.

The *in silico*-fragmented spectra library provides the opportunity to perform identification by spectra matching software tools, and was integrated into LPPtiger using a reverse dot-product algorithm^17^. However, the large diversity of LPPs structures required additional scoring systems to improve the identification quality and reduce the rate of false positive identification. Thus, LPPtiger features an unique architecture based on five individual scores, each having its own specific focus and providing unique information for LPP identification (Fig. 4):

1. The Spectra Similarity Score uses the spectra matching approach, which compares measured spectra with *in silico*-generated LPPs spectra libraries using a reverse dot-product algorithm^17^.
2. The Rank Score provides an intensity-dependent score based on a bottom-up strategy by implementing an intensity-ranked comparison of observed fragment ions with a predicted white list of oxidized fatty acyl residues and PL head group-specific signals^18^. The Rank Score helps to assign structure-related signals of low intensity with no bias for *sn*-1 or *sn*-2 FA residues assignment by default settings. However, it can be tuned to distinguish potential isomers by applying customized weight factors to *sn*-1 or *sn*-2 FA residue-related signals based on observed distributions of relative intensities for specific instruments.
3. The Fingerprint Score is based on the modification type-specific intensity-independent scoring algorithm. In contrast to the dominant fragment ions, modification-specific fragments and neutral losses (e.g., water losses and prostane ring specific ions) may not have consistent relative intensities, or be represented by low intensity signals. However, these low intense signals, especially the combination of several water losses and head group-specific losses, can significantly improve the specificity of the identification. The Fingerprint Scoring algorithm uses an extended *m/z* list of fragment ions, neutral losses and all possible modification-related signals generated during *in silico* oxidation as a white list matched to the measured spectra. Since it is an intensity-independent algorithm, even low abundant modification related signals can significantly contribute to the final identification score. With the contribution of the Fingerprint Score, keto and hydroxy groups containing isomers of PL differed by one double bond can be successfully distinguished.
4. The Specificity Score utilizes adapted signal-to-noise function calculations^19^, aimed at indicating the ratio between specific signals supporting the identification of certain LPPs (treated as “signal”) versus signals corresponding to the other species (treated as “noise”). The Specificity Score can differentiate multiple LPPs identified from the same tandem mass spectrum by the sum of the intensities of their structure-specific signals. It was shown to be effective for identification of discrete LPPs from partially co-eluting signals or MS/MS spectra acquired in the valley of two chromatographic peaks corresponding to the neighboring isomeric LPPs.
5. The Isotope Score provides an isotope distribution check, which is commonly used to evaluate the fit between the elemental composition of the proposed structure and the observed isotope distribution pattern^20^.

The overall LPPtiger identification score merges these five individual scores, representing the identification quality from the above-mentioned aspects, and is used as the main criteria in the LPPtiger output. The unique LPPtiger scoring system reduces the complexity of overall decision making through its use of a single overall score.

LPPtiger is a Python-based tool with a graphical user interface (Supplementary Figure S3) and the source code is freely available for both Windows- and Linux-based operating systems. Additional compiled executable (.exe) file is also available for Windows platforms. LPPtiger natively supports parallel processing, with a build-in batch mode to accelerate the identification process (Supplementary Figure S4). Current version of LPPtiger supports prediction and identification of PL-bound LPPs from five main PL classes (PC, PE, PS, PG, and PA) base on a negative ionization mode DDA datasets obtained using Waters and ThermoFisher Scientific MS instruments. Future updates will support PI, PIP, phosphosphingolipids, ceramides, di- and triglycerides as well as input files from other MS vendors.

**Figure 5:**
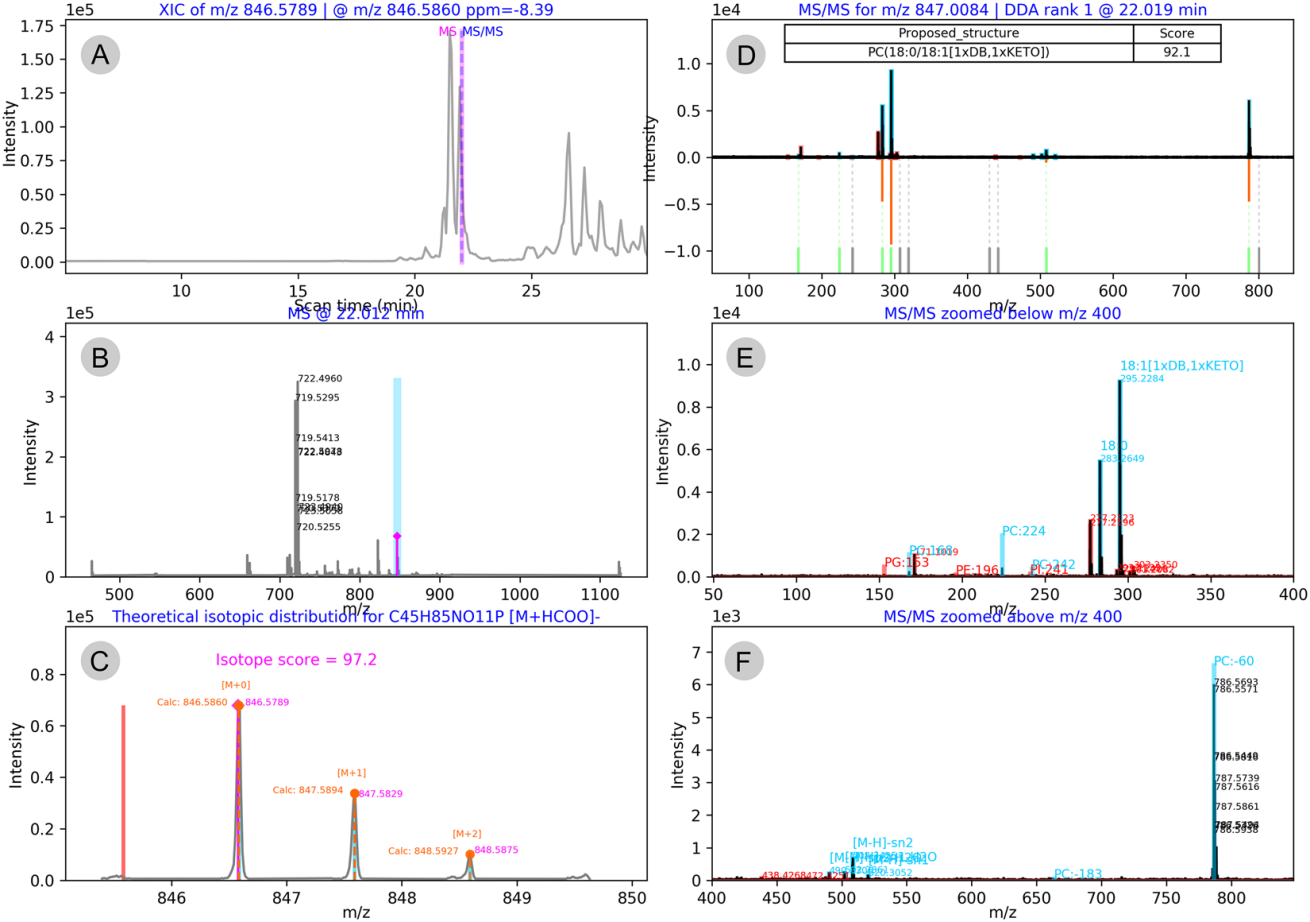
Example of the six-panel image from LPPtiger .html report for precursor at *m/z* 846.5789 identified as PC(18:0/18:1[1xDB,1xKETO]). Image summarizing extracted ion chromatogram (**A**), corresponding MS scan (**B**), zoomed MS scan with isotope pattern and Isotope Score (**C**), MS/MS spectrum used for the identification (**D**), MS/MS spectrum zoomed in on the region of fatty acid fragments (**E**) and neutral loss signals (**F**).

The final identification results are reported via a detailed excel table providing all main parameters – proposed LPP structure, elemental composition, theoretical and observed *m/z* values, mass accuracy, retention time, identification metrics (i.e., all scores, relative intensities of matched fragments, LPP specific and unspecific signals), and data specific details (e.g., DDA rank and scan number) (Supplementary Table 3). Furthermore, LPPtiger provides a graphical representation of each identification via a six-panel image integrated into an interactive HTML report file available for manual reviewing (Fig. 5, Supplementary Figure S5). Each identified LPP is supported by the image of precursor-specific XICs (Fig. 5A), a corresponding MS scan (Fig. 5B), a zoomed MS scan illustrating the isotope pattern and calculated Isotope Score (Fig. 5C), an MS/MS scan used for identification illustrating Spectra Similarity, Ranked and Fingerprint Scores, as well as a final LPP ID with an overall LPPtiger Score (Fig. 5D), zoomed region of fatty acid fragments (Fig. 5E), and neutral loss signals (Fig. 5F). A detailed step-by-step LPPtiger manual (Supplementary File 11) together with a training dataset is available at https://bitbucket.org/SysMedOs/lpptiger/.

LPPtiger was first validated using DDA LC-MS datasets of *in vitro*-oxidized PL standards. The sample set included PC, PE, PS, PG, and PA lipids with different compositions of esterified PUFA to provide complex oxidation patterns. Samples were separated by RP-HPLC coupled on-line to an ESI-QqTOF-MS operating in negative ion mode. Data were acquired using DDA and used for cross-validation of LPPtiger specificity. LPPtiger was found to demonstrate a high specificity of LPP identification (Supplementary File 12, Examples 1–12; Supplementary Table 4).

A final showcase of LPPtiger performance was performed using a DDA LC-MS dataset acquired from lipidomes of rat primary cardiomyocytes (CM) treated with peroxynitrite donor SIN-1 for 15, 30, 70 min and 16 h. Low SIN-1 concentrations (10 µmol/L) induce mild nitroxidative stress in treated cells, which is characterized by a low level of protein and lipid oxidation^21^. A significant increase in oxidized lipids and their accumulation in perinuclear space was demonstrated using fluorescent microscopy^21^. However, detailed MS-based identification of PL-bound LPPs had not been performed thus far. The cellular model of mild nitroxidative stress used here for LPPtiger validation imposes a number of challenges, including a low level of induced stress as well as the dynamic nature of the model (five time points over the course of the experiment). Furthermore, due to the high complexity of CM lipidome, large number of potential isomeric and isobaric species can be expected. Thus, combination of optimized LC separation with high resolution MS detection is critical to address oxLipidome diversity (Supplementary Figure S6). Low abundance of endogenous LPPs requires optimized separation and sensitive detection strategies^22–24^. Coupling of reverse phase liquid chromatography to MS often provides a possibility to separate oxPLs from a bulk of native PLs. Furthermore, untargeted MS methods such as DDA have to be tuned for the detection of low abundant LPPs (e.g. using low intensity threshold to select precursor ions for the fragmentation) and structural LPP isomers (e.g. optimizing DDA dynamic exclusion parameters). One should also consider that lipids in general and LPPs in particular are very labile and can undergo artificial oxidation during sample preparation step. Thus, application of antioxidants such as butylated hydroxytoluene, EDTA and glutathione at all steps of sample handling are required to prevent artificial lipid oxidation^25^.

**Figure 6:**
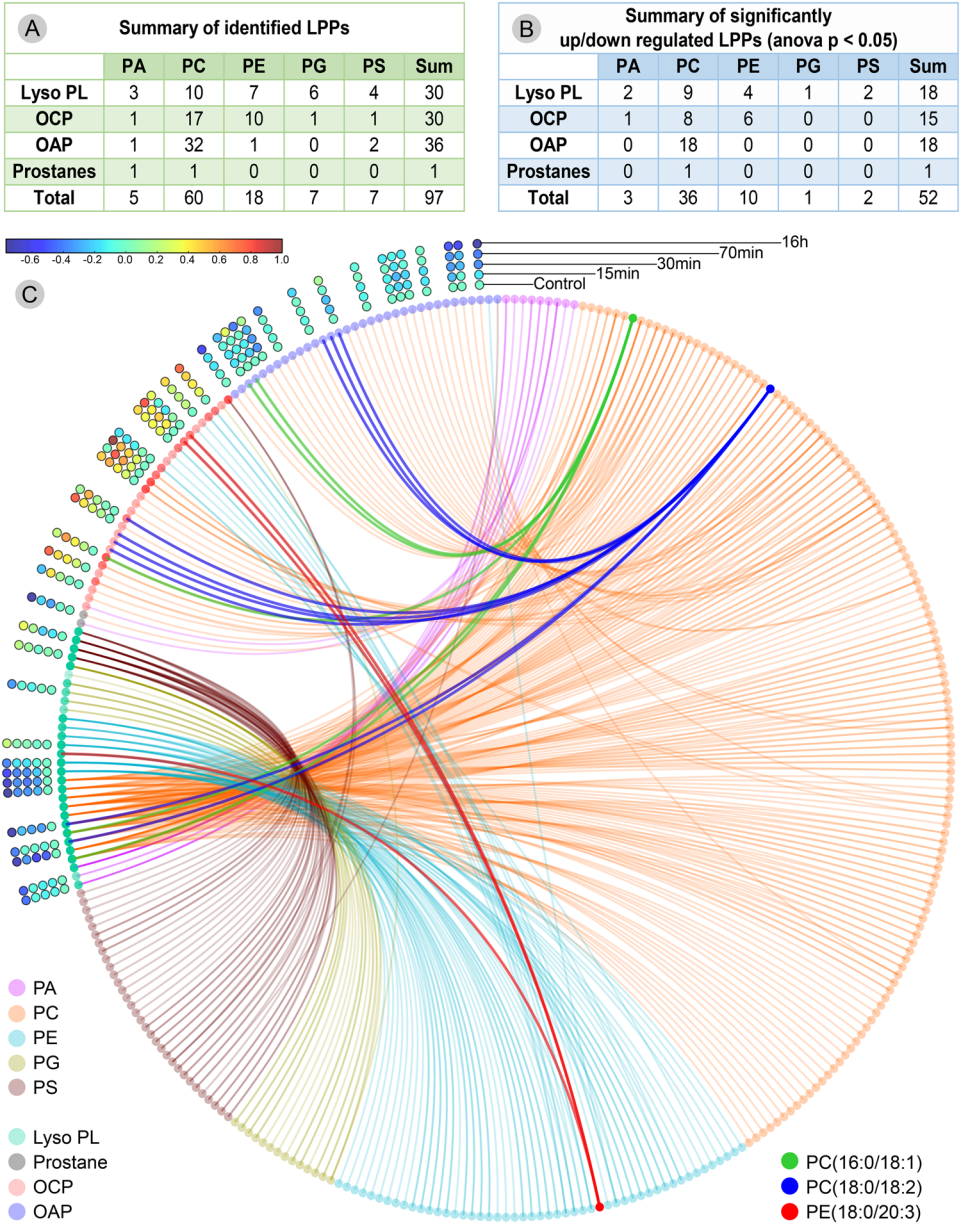
Summary of LPPtiger identified and relatively quantified PL-LPPs in lipidome of SIN-1 treated cardiomyocytes. (**A**) – LPPs including lysoPL, oxidative cleavage (OCP), oxygen addition products (OAP), and prostanes from five PL classes identified by LPPtiger. (**B**) – Significantly regulated (ANOVA p ≤ 0.05) PL-LPPs. (**C**) – Circos diagram illustrating relationship between parent PL species and identified/quantified LPPs.

The previously identified native CM lipidome, characterized by 202 discrete lipid species from six PL classes^18^, was used by the LPPtiger for oxLipidome prediction. The application of an *in silico* oxidation algorithm resulted in 22,817 predicted LPPs (530 oxPA, 10,458 oxPC, 5,085 oxPE, 3,875 oxPG, 2,869 oxPS) summarized as .sdf libraries (available at https://bitbucket.org/SysMedOs/filesrepository). Corresponding .msp spectra libraries were generated for predicted LPP structures (available at https://bitbucket.org/SysMedOs/filesrepository) and used for LPPtiger assisted identifications. Overall, 30 lysoPL and 67 PL-bound LPPs were identified in the CM lipidome, including 60 PC-, 18 PE-, 7 PG-, 7 PS-, and 5 PA-derived species, of which 30 were OCP, 36 OAP, and two prostane-containing LPPs (Fig. 6A, Supplementary Table 5). To the best of our knowledge, this represents the first dataset reporting such a high number of LPPs from five PL classes identified from untargeted lipidomics data. Examples of LPPs from each lipid class identified by LPPtiger in CM lipidomes together with a corresponding original CID tandem mass spectra are provided in Supplementary File 12 (Examples 13–20).

LysoPLs, known to be formed via oxidation-induced de-esterification of one of the FA residues, were distributed over all five detected PL classes, with the highest number identified for PC and PE lipids. OAP LPPs were represented by single keto (13 PC), single hydroxy (12 PC and one PS), double hydroxy or peroxy (two PC and one PS), and triple hydroxy or hydroxy and peroxy (six PC and one PE) derivatives. Most of the identified OAP LPPs were derived from PC lipids, whereas OCP were more evenly distributed among different PL classes. Most of the truncated PLs were represented by terminal carbonyls (28 out of 30) formed by a cleavage at positions four, five, seven, nine, ten or twelve of FA carbon chains, well corresponding to the general mechanisms of PUFA oxidation. Thus, five carbon-long OCPs are characteristic products of arachidonic acid oxidation. LPPtiger identified four LPPs carrying five carbon-long truncated moieties, including PE(18:0/5:0 < CHO@C5>), PE(18:0/5:0 < COOH@C5>), PS(18:0/5:0 < CHO@C5>), and PC(20:5/5:0 < CHO@C5>). Another prominent example are LPPs containing oxo-nonanoyl moiety formed by an oxidative cleavage of linoleic acid at C9 position. LPPtiger identified five oxo-nonanoyl-containing lipids corresponding to one PE and four PCs. PC-derived, C9-truncated LPPs were nicely LC-separated in agreement with the structure of a second fatty acyl present (e.g., 18:2 vs 16:0 vs 18:1 vs 18:0). Despite a high specificity of LPPtiger towards prostane-containing LPPs demonstrated on *in vitro*-oxidized standards, only two prostane LPPs, namely PA(18:1/A3-dihomo-IsoP) and PC(16:0/H1-phytoP), were identified in CM.

LPPtiger demonstrated a high specificity for the identification of isomers. Thus, isomeric species PC(18:1/18:1[1xDB, 1xKETO]) and PC(18:0/18:2[1xDB, 1xOH]) with the *m/*z 844.5704 (formate adduct) were identified at two different retention times – 19.7 and 22.2 min, respectively (Supplementary Figure S7). Similarly, three isobaric/isomeric PC-derived OCPs, PC(16:0/8:1[1xDB,1xOH] < CHO@C5>), PC(16:0/9:0 < CHO@C9>), and PC(18:0/7:0 < CHO@C7>) with precursor *m/z* 694.39, 694.43 and 694.43 were successfully distinguished and assigned to the signals with characteristic retention times (9.7, 10.7 and 12.2 min, respectively).

Changes in LPPtiger-identified oxidized PLs were relatively quantified using Progenesis QI. Out of 97 identified LPPs, 52 showed significant (ANOVA p ≤ 0.05) up or down regulation within five experimental time points (Fig. 6B and Supplementary Table 6). Out of 52 regulated species, 18 corresponded to lysoPLs, 15 to OCP LPPs, and 18 OAP LPPs. The PCA analysis of relatively quantified LPPs clearly separated experimental groups (with more than 74% of the data explained by principal components 1 and 2), showing the differences between control and 16 h SIN-1-treated cells (Supplementary Figure S8). Furthermore, 15 min and 30 min experimental time points were found to be more similar to each other than to 70 min and control. A hierarchical clustering analysis of relatively quantified LPPs demonstrated a clearly distinct behaviour of OCP- and OAP-oxidized lipids, whereas lysoPL species showed more diverse behaviour (Supplementary Figure S9). Using a circos diagram generated by an in-house developed script LipidCircos (https://bitbucket.org/SysMedOs/lipidcircos), the relationship between precursor PLs and corresponding OCP LPPs and OAP LPPs was demonstrated (Fig. 6C). Exemplified by PC(16:0/18:1) (dark green), PC(18:0/18:2) (dark blue), and PE (18:0/20:3) (dark red), the system demonstrated a significant elevation of truncated PL-LPPs by reducing OAP PL-LPPs and LysoPLs, while increasing OCP PL-LPPs. Moreover, the accumulation of truncated carbonylated PLs is in agreement with fluorescent microscopy imaging of cellular carbonyls^21^.

In summary, LPPtiger is an open-source software tool freely available for download (https://bitbucket.org/SysMedOs/lpptiger), and can be easily tuned by the user based on the experiment type, instrument used, set of selected PL classes, and modification types. LPPtiger relies on the combination of three unique algorithms and allows for the prediction of sample-specific oxidized lipidome using a discrete FA composition of native PLs. This software also generates an LPP-specific tandem mass spectra library, which can be further used for LPP identification based on the five scoring algorithms and thus increasing sensitivity and specificity of the results. Applied to large LC-MS/MS datasets powered by initial parallel processing support, LPPtiger can provide a systems wide view on oxPL lipidome in an high-throughput untargeted manner.

## Methods

### Chemicals

Phospholipid standards were obtained from Avanti Polar Lipids (AL, USA): 1,2-diheptadecanoyl-*sn*-glycero-3-phosphate [PA(17:0/17:0)], 1,2-diheptadecanoyl-*sn*-glycero-3-phosphocholine [PC(17:0/17:0)], 1,2-diheptadecanoyl-*sn*-glycero-3-phosphoethanolamine [PE(17:0/17:0)], 1,2-diheptadecanoyl-*sn*-glycero-3-phospho-(1′-*rac*-glycerol) [PG(17:0/17:0)], 1,2-diheptadecanoyl-*sn*-glycero-3-phospho-L-serine [PS(17:0/17:0)], 1-palmitoyl-2-oleoyl-*sn*-glycero-3-phosphate (POPA), 1-palmitoyl-2-linoleoyl-*sn*-glycero-3-phosphate (PLPA), 1-palmitoyl-2-oleoyl-*sn*-glycero-3-phosphocholine (POPC), 1-palmitoyl-2-linoleoyl-*sn*-glycero-3-phosphocholine (PLPC), 1-palmitoyl-2-oleoyl-*sn*-glycero-3-phosphoethanolamine (POPE), 1-palmitoyl-2-linoleoyl-*sn*-glycero-3-phosphoethanolamine (PLPE), 1-palmitoyl-2-arachidonoyl-*sn*-glycero-3-phosphoethanolamine (PAPE), 1-palmitoyl-2-oleoyl-*sn*-glycero-3-phosphoglycerol (POPG), 1-palmitoyl-2-linoleoyl-*sn*-glycero-3-phosphoglycerol (PLPG), 1-palmitoyl-2-oleoyl-*sn*-glycero-3-phospho-L-serine (POPS), and 1-palmitoyl-2-linoleoyl-*sn*-glycero-3-phospho-L-serine (PLPS). Acetonitrile, methanol, isopropanol and formic acid were purchased from Biosolve (Valkenswaard, Netherlands). Dulbecco’s Modified Eagle Medium/Ham’s F-12 (DMEM/F12), phosphate buffered saline (PBS), foetal bovine serum (FBS), penicillin-streptomycin, L-glutamine, non-essential amino acids, sodium pyruvate, and gelatine were purchased from Life Technologies GmbH (Darmstadt, Germany). Horse serum, trypsin-EDTA solution, and butylated hydroxytoluene (BHT) were obtained from Sigma-Aldrich GmbH (Taufkirchen, Germany). 3-Morpholinosydnonimine (SIN-1) was purchased from Enzo Life Sciences GmbH (Lörrach, Germany).

### PL standards oxidation

Lipid vesicles were prepared from five PL classes (PA: POPA and PLPA; PC: POPC and PLPC; PE: POPE, PLPE, and PAPE; PG: POPG and PLPG; and PS: POPS and PLPS) separately. PL standards (650 µg each; 10 mg/mL in chloroform) were diluted in chloroform (300 µL), dried under a stream of nitrogen and rehydrated in ammonium bicarbonate (143 µL; 3 mmol/L). Samples were sonicated for 15 min to form lipid vesicles. PL vesicles (125 µL; 6 mmol/L) were mixed with CuSO_4_ (50 µL; 0.75 mmol/L), ascorbic acid (50 µL; 1.5 mmol/L), and water (275 µL) followed by 72 h incubation at 37 °C^26,27^.

### Cell culture

Primary rat cardiomyocytes (Innoprot, Elealde Derio, Spain) were cultured in gelatine-coated 6-well-plates (CELLSTAR®, Greiner Bio-One GmbH, Frickenhausen, Germany) in DMEM/F12 medium supplemented with 20% FBS, 5% horse serum, 2 mmol/L L-glutamine, 3 mmol/L sodium pyruvate, 0.1 mmol/L non-essential amino acids, 100 U/mL penicillin and 100 µg/mL streptomycin at 37 °C (humidified atmosphere of 5% CO_2_ and 95% air). When cells reached 80% confluence, medium was replaced by serum-free medium (DMEM/F12 supplemented with 100 U/mL penicillin and 100 µg/mL streptomycin), and cells were treated with 10 µmol/L SIN-1. After 15 min, 30 min, 70 min, or 16 h, plates were placed on ice, and cells were washed with cold PBS containing BHT (0.01%, w/v) and scraped into 1 mL of cold methanol containing acetic acid (3%, v/v) and BHT (0.01%, w/v)^28^. Samples were spiked with internal standards [PC(17:0/17:0), PE(17:0/17:0), PS(17:0/17:0), PG(17:0/17:0), PA(17:0/17:0); 100 ng per sample), dried, and resuspended in 50 µL of a mixture of water, isopropanol, acetonitrile, and methanol (5:3:1:1, by volume).

### RPLC-MS

Acquity UPLC M-class (Waters GmbH, Eschborn, Germany) was coupled online to a Synapt G2-Si mass spectrometer equipped with an ESI source (Waters GmbH, Eschborn, Germany) operating in negative ion mode. Eluent A was a mixture of water and acetonitrile (90:10, v/v) containing formic acid (0.1%, v/v), and eluent B was a mixture of isopropanol, acetonitrile, and methanol (60:20:20, v/v/v) containing formic acid (0.1%, v/v). Lipids (1 μL in 50% B; each sample in triplicate) were loaded onto a C_18_-column Acquity UPLC® CSH^TM^ C18, (internal diameter 1.0 mm, length 100 mm, particle diameter 1.7 μm) and eluted with linear gradients from 50 to 90% eluent B (30 min) and to 99% B (1 min, and held for 10 min). Column temperature was set to 50 °C and the flow rate to 60 μL/min.

Sampling cone voltage was set to 40 V, source offset to 60 V, source temperature to 120 °C, cone gas flow to 30 L/h, desolvation gas flow to 650 L/h, desolvation temperature to 250 °C, nebuliser gas pressure of 6 bar, and an ion spray voltage of -2.0 kV. Data were acquired in negative ion data-dependent (DDA) and independent (MS^E^) resolution modes. In the DDA mode, precursor ion survey scans (scan time 0.5 sec) were acquired for *m/z* 200 to 1200. Minimum precursor intensity threshold for CID selection was set to 1000 counts. Tandem mass spectra were acquired for *m/z* 50 to 1200 with a ramp collision energy (LM CE start/end 10–40 and HM CE start/end 20–60). MS/MS spectra were recorded (scan time 0.25 sec or till total ion current reached 100,000 counts) for the 12 most intense signals in each survey scan using a dynamic exclusion for 30 sec. For MS^E^ low (3 V) and high collision energy scans (ramp from 20 to 50 V, scan time 0.6 sec) were acquired for *m/z* 50 to 1200. The signal of Leu-encephalin (554.26151^−^) was acquired as lock mass. DDA data were used for LPPtiger based identification of oxPLs. MS^E^ data were used only for relative label free quantification.

### Software assisted data analysis

DDA datasets were converted into mzML format using the MSConvert module from ProteoWizard project (version 3.0.9134 64 bit)^29^ and processed by an in-house developed software LPPtiger to assist high-throughput PL-bound LPP identification. All mzML files are available at https://bitbucket.org/SysMedOs/filesrepository/downloads/. LPPtiger was developed using Python programing language (version 2.7) with RDkit package to operate compound structures^30^, pymzML package^31^ to process mzML files, and other packages, such as Numpy, Pandas, Scipy, and Matplotlib for data processing and visualisation. LPPtiger provides a cross platform Graphic User Interface (GUI) that can be executed on Windows and Linux platforms, the project repository is hosted on Bitbucket (https://bitbucket.org/SysMedOs/lpptiger).

### Lipid abbreviations

LPPs were abbreviated using an extended nomenclature of modification name spaces based on the discrete nomenclature of phospholipids proposed by the LIPID MAPS consortium^32^. The OCP LPPs were indicated by the corresponding terminal enclosed in angular brackets (e.g. “<” and “>”), with the truncation site indicated by the carbon atom number (e.g., <COOH@C9> and <CHO@C12>, which can be found in Supplementary Figure S10A and B). The modification types and numbers were enclosed in a pair of square brackets, and the number of double bonds on the carbon chain were also marked (Supplementary Figure S10C).

### Reconstruction of PUFA oxidation networks

Networks summarizing oxidation of the ten most abundant PUFA [linoleic (∆^9,12^ 18:2), alpha-linolenic (∆^9,12,15^ 18:3), eicosapentaenoic *n-3* (∆^5,8,11,14,17^ 20:5), docosapentaenoic *n-3* (∆^7,10,13,16,19^ 22:5), docosahexaenoic *n-3* (∆^4,7,10,13,16,19^ 22:6), gamma-linolenic *n-6* (∆^6,9,12^ 18:3), dihomo-gamma-linolenic *n-6* (∆^8,11,14^ 20:3), arachidonic *n-6* (∆^5,8,11,14^ 20:4), docosatetraenoic *n-6* (∆^7,10,13,16^ 22:4), docosapentaenoic *n-6* (∆^4,7,10,13,16^ 22:5)] were reconstructed based on a literature meta-study (Supplementary Table 1) using CellDesigner 4.4^33^. Networks include 490 (native and oxidized) LPPs and 569 reactions (enzymatic and non-enzymatic) are available as SBML^34^ files (Supplementary Files 1–10) and images at https://bitbucket.org/SysMedOs/filesrepository. PUFA and LPPs are defined using a “simple molecules” symbol, interconnected with straight lines and filled arrow heads to illustrate the reactions. Enzymes catalyzing the reactions are denoted by circle-headed lines and illustrated using a “protein” symbol. For non-enzymatic reactions driven by reactive oxygen species, ROS are marked using a “simple molecule” symbol.

### *In silico* oxidation algorithm

PL *in silico* oxidation was performed by a systematic enumeration of all possible combinations of modification types and amounts based on the number of double bonds in each FA residue in the initial PL structure. Initial PLs, e.g., PE(16:0/20:4), were converted into SMILES (simplified molecular-input line-entry system)^35^ representations of the structure, decomposed, and reconstructed for each FA residue. For the selected unsaturated FA residues, SMILES segments representing double bond units together with neighboring bis-allylic positions were transformed into eight oxygen additions and four oxidative cleavage modifications (Supplementary Figure S1) based on the predefined SMILES in the configuration files (Supplementary Table 7). The combinatorics was controlled and filtered to keep only LPPs fulfilling user-defined criteria in the number and type of the modifications (e.g., maximum of three oxygen additions per LPP). In the current version of LPPtiger, all positional isomers with same number and type of modifications were merged into one entry in the structure library. The list of SMILES and corresponding abbreviations resulting from *in silico* oxidation of FA residues was used to reconstruct oxPL. A similar strategy was applied to prostanes and other more specific structures (e.g., thromboxanes and levuglandins) using predefined SMILES codes with a three-double bond. However, the positional isomers of prostanes were retained and abbreviated separately to maintain the possibility of specific identification. After *in silico* oxidation of all initial PLs, the generated SMILES were converted into .mol files and annotated with an additional 18 descriptors (Supplementary Figure S11). The annotated .mol files were exported as a single .sdf file, which could also be analyzed using other software, e.g, ChemAxon InstantJ^36^, Progenesis QI (version 2.1.0, Nonlinear, Newcastle, UK), and Progenesis SDF studio (version 1.0, Nonlinear, Newcastle, UK). Summary tables for essential descriptors were saved in .xlsx format for all LPPs and all FA residues (with and without *in silico* oxidation) for fast reviewing and for use as input files for further identification steps.

### *In silico* fragmentation algorithm

*In silico* fragmentation algorithm for predicted LPPs is based on the summarized knowledge of LPP MS/MS patterns. The algorithms include two major parts – the generation of *in silico* fragmentation spectra and the fingerprint *m/z* list. *In silico* fragmentation routes were formulated based on the data collected for *in vitro*-oxidized PL standards (Supplementary Table 2). Since the relative intensity of the fragmentation patterns are instrument-dependent, the optimization of fragmentation rules is crucial for the *in silico* prediction of tandem mass spectra. Product ions were predicted as structure-specific elemental compositions, and further annotated with a corresponding *m/z* value, relative intensity and ion type. *In silico* generated spectra are stored as a JSON (JavaScript Object Notation) string in the .sdf structure library, as .xlsx output, and as a .msp spectra file compatible with NIST MS Search and MSpeptide search tools^37^.

The Fingerprint *m/z* lists were generated by using elemental compositions of PL head groups and FA residue-specific fragments and neutral losses, as well as their combinations, to calculate all possible combinations of product ions including corresponding water losses. Calculated lists of elemental compositions were converted into *m/z* values to be stored in the .sdf structure library, and as .xlsx output.

### LPPtiger scoring algorithms

LPPtiger uses several scoring algorithms to generate the overall identification score for each proposed LPP structure. Each of the scoring algorithms has its own specific focus and provides unique information. The current overall score is generated from the average of the five individual scores, including Spectra Similarity Score, Rank Score, Fingerprint Score, Specificity Score, and Isotope Score.

### Spectra Similarity Score

As one of the most widely used classical algorithm of spectra matching^38^, the dot-product similarity scoring algorithm was incorporated into LPPtiger as a robust scoring method for spectra search-based identification strategies. A weight factor W is calculated (Equation 1) to represent each *m/z* and intensity pair, and further used for dot-product similarity scoring (Equation 2).

1 $$W={[peakintensity]}^{m}{[m/z]}^{n}$$

2 $$Spectra\,Similarity\,Score=100\times \frac{{({\sum }^{}{W}_{lib}{W}_{obs})}^{2}}{{\sum }^{}{{W}_{lib}}^{2}\,{\sum }^{}{{W}_{obs}}^{2}}$$

The default values of m and n are set to 0.6 and 3, respectively, according to Stein *et al*.^38^. These values can be changed based on the instrument used, e.g., m = 0.5 and n = 1^39^, m = 0.5 and n = 2^40^, or any other combinations.

Introduced by the NIST MS Search program^37^, the reverse dot-product algorithm modified the original algorithm published by Stein *et al*.^38^ in such a way that only signals matched to the user spectra are considered. In the reverse dot similarity algorithm, the match score is not penalized by unmatched or impurity signals. Of note is that LPPtiger generates a spectra similarity score using only the reverse dot-product algorithm.

### Rank Score

The Rank Score is a bottom-up strategy scoring system upgraded from LipidHunter software^18^ in which discrete LPP species are identified by comparing each signal of an MS/MS peak list with a predefined list of possible FA and PL head group-specific fragment and neutral loss signals, generated from the *in silico* oxidation step, and can be customized based on the .xlsx template. A list of all possible LPP *sn-1* and *sn-2* residues generated by an *in silico* oxidation algorithm, together with a list of all PL head group-specific signals, are used as a white list of FA and PL class specific signals. The observed tandem mass spectra (peak lists) are then compared against the white list. The top ten most intense matched signals corresponding to FA fragment or FA neutral loss ions are ranked by intensities and subsequently indexed (RankIndex). The Rank factor R_frag_ is calculated for each matched signal using Equation 3.

3 $${R}_{frag}=\frac{(10-(RankIndex-1))}{10}\,\times 100 \% \,$$

The Rank Score is calculated (Equation 4) as the sum of identification-supporting Rank factors multiplied by the instrument-dependent, user definable Weight factor W_frag_ (Supplementary Table 7).

4 $$Rank\,Score={\sum }^{}{W}_{frag}\times {R}_{frag}$$

### Fingerprint Score

As described in the *in silico* fragmentation algorithm section, all LPPs upon CID have specific neutral losses and product ions. Thus far, *in silico* spectra generated by a fragmentation algorithm only considers the main fragments, since some fragments and neutral losses might not have consistent relative intensities, or present as a low intensity signals. However, these low intensity signals – especially the combination of several water losses and head group- specific losses – can significantly support the identification of certain modifications. A fingerprint scoring algorithm predicts all possible combinations of modified FA acyl residues, as well as PL head group-specific fragments and neutral loss ions (e.g., water losses, prostane rings specific fragments) as a list of predicted *m/z* values. Any match between *m/z* value in fingerprint list with a signal in observed MS/MS spectrum will be marked as 1, while fingerprint *m/z* value with no match in observed MS/MS spectrum will be marked as 0. The reverse dot-product of the observed fingerprint list (a list of 0 and 1) versus the entire fingerprint list (a list of 1) will be calculated using cosine similarity algorithm (Equation 5).

5 $$Finger\,print\,Score=100\times \frac{{F}_{obs}\cdot {F}_{lib}}{\parallel {F}_{obs}{\parallel }_{2}\parallel {F}_{lib}{\parallel }_{2}}$$

Application of the Fingerprint Score allows structural isomers to be distinguished which are differed by one double bond in combination of hydroxy versus keto functional groups.

### Specificity Score

A list of signals supporting certain LPP identifications and identified by the Rank Score, Spectra Similarity Score, and Fingerprint Score is generated and compared with the list of LPP-unspecific signals provided by the Rank Score algorithm (e.g., signals matched to other FA or head group combinations). The Specificity Score indicates the confidence of particular LPP assignments in comparison to all other possibilities supported by unspecific signals. Considering that a dynamic range of summed signal intensities can be distributed across several orders of magnitudes, the signal to noise ratio function by decibels definition^19^ was used to calculate the Specificity Score (Equation 6).

6 $$Specificity\,Score={F}_{amplify}\times 20\times {\mathrm{log}}_{10}(\frac{{\sum }^{}intensity\,of\,signals\,support\,identified\,structure}{{\sum }^{}intensity\,of\,signals\,support\,other\,structures})$$

The amplification factor F_amplify_ was introduced as a user definable factor to amplify the score of certain intensity ratios to 100. By default, this value is set to 10.4795, resulting in a Specificity Score of 100 when the ratio of structure-specific and unspecific signals is equal to 3. All Specificity Scores above 100 or below 0 are set to 100 and 0, respectively. In case no structure-unspecific signal is identified, the Specificity Score is set to 100.

### Isotope Score

The Isotope Score is calculated using the previously reported algorithms^20^. The LPPtiger Isotope Score algorithm consists of two primary steps: the prediction of the isotope distribution by means of binomial and McLaurin expansion (Equation 7) and similarity score of the relative intensity distribution by calculating the similarity between experimental and theoretical isotope distributions.

For instance, the following [M + HCOO]^−^ adduct of PC(18:0/18:1[1xDB,1xKETO]) with an elemental composition of C_45_H_85_NO_11_P^−^ is as follows (phosphorus as a monoisotopic element is not considered in the equation).

7 $$\begin{array}{c}{({}^{12}C+{}^{13}C)}^{45}{({}^{1}H+{}^{2}H)}^{85}({}^{14}N+{}^{15}N){({}^{16}O+{}^{17}O+{}^{18}O)}^{11}\\ \quad =({}^{12}C_{45}{}^{1}H_{85}{}^{14}N{}^{16}O_{11}){(1+\frac{{}^{13}C}{{}^{12}C})}^{45}{(1+\frac{{}^{2}H}{{}^{1}H})}^{85}(1+\frac{{}^{15}N}{{}^{14}N}){(1+\frac{{}^{17}O}{{}^{16}O}+\frac{{}^{18}O}{{}^{16}O})}^{11}\end{array}$$

Afterwards, the Isotope Score is generated using Equation 8 based on the observed intensities of signals from [M+0] to [M+i], while I_M_ and I_M+i_ refer to their signal intensities, respectively.

8 $$Isotope\,Score=100\times (1-{\sum }^{}|{r}_{obs.i}-{r}_{lib.i}|)with\,{r}_{i}=\frac{{I}_{M+i}}{{I}_{M}},1\le i\le 2$$

### LPPtiger Score

When it is possible to calculate and overcome defined thresholds for all five scores provided above, the overall LPPtiger Score is assigned to the proposed LPP identification by averaging all five individual scores (Equation 9):

9 $$LPPtiger\,Score=\frac{(Rank\,S+Spec\,Sim\,S+FpS+Specif\,S+Isotope\,S)}{5}\,$$

The LPPtiger Score is used as the main representative value of the identification quality and reported in the HTML reports and six-panel assigned spectra images, while the individual scores can be accessed in the final results summary table.

### Parallel and batch processing mode

The identification of LPPs from *in silico*-predicted libraries with five-criteria LPPtiger scores from large LC-MS/MS datasets demands a large source of computing power and could be a time consuming process. Thus, a native three-level parallel processing workflow was integrated into LPPtiger to accelerate the identification process (Supplementary Figure S4). The basic level of parallel processing (Lv1) is to run multiple files in parallel, which allows for a full CPU load, yet results in inefficient RAM usage.

The core level of parallel processing (Lv 2) distributes a task into multiple sub-processes within three phases of the identification process (Lv2.1 - precursor match, Lv2.2 - XIC extraction, and Lv 2.3- MS/MS assignment and scoring). Level 2 parallel processing is extremely intensive for both CPU and RAM load, but provides the most significant acceleration of the entire process.

Level 3 (Lv3) parallel processing utilizes vectorized functions to accelerate the calculation of precursor selection windows, mass tolerance, and mass accuracies. Moreover, the vectorized functions can take advantage of multicore CPUs. Optimization of GPU-based parallel processing is currently under development.

Using a three-level parallel processing workflow gradually reduces the processing time from over four hours (single thread algorithm) to less than one hour (four CPU cores and 8 GB RAM configuration). Since a performance check using up to 16 CPU cores did not exhibit any significant improvement in processing time over five CPU cores on a single file (Supplementary Figure S12), we recommend to run multiple files in parallel, with each file assigned to five CPU cores and 10 GB of RAM as an optimal configuration.

LPPtiger provides an integrated batch mode to process multiple files in parallel or as a sequence. Using batch mode and parallel processing of 15 LC-MS/MS datasets (3.5 GB), the identification time was significantly reduced from over one month to less than five days for LPP identification from five PL classes.

### Relative label free quantification

Datasets acquired in MS^E^ mode were imported into Progenesis QI (version 2.1.0, Nonlinear, Newcastle, UK). PLs identified by LipidHunter from DDA data were matched to the corresponding features (*m/z* values and retention time) in aligned MS^E^ datasets. Spiked internal standards were used for normalization.

PLs detected with significantly different intensities (ANOVA p value ≤ 0.05) were exported and further analyzed by EZinfo (version 1.0, MKS Instruments, Crewe, UK) and Genesis (version 1.7.7)^41^. A circos diagram^42^ was generated by customized python scripts named LipidCircos based on Circos package (version 1.3.5)^43^, and the scripts are freely available at https://bitbucket.org/SysMedOs/lipidcircos.

### Data availability

LPPtiger source code is freely available at https://bitbucket.org/SysMedOs/lpptiger. Fatty acid oxidation networks are available as SBML files and images at https://bitbucket.org/SysMedOs/filesrepository. LipidCircos source code is freely available at https://bitbucket.org/SysMedOs/lipidcircos. The datasets generated during and/or analysed during the current study are available at https://bitbucket.org/SysMedOs/filesrepository/downloads/.

## Electronic supplementary material

Supplementary Information

Supplementary Table 1

Supplementary Table 2

Supplementary Table 3

Supplementary Table 4

Supplementary Table 5

Supplementary Table 6

Supplementary Table 7

Supplementary File 1

Supplementary File 2

Supplementary File 3

Supplementary File 4

Supplementary File 5

Supplementary File 6

Supplementary File 7

Supplementary File 8

Supplementary File 9

Supplementary File 10

Supplementary File 11

Supplementary File 12
