# Supplementary material for: LPPtiger software for lipidome-specific prediction and identification of oxidized phospholipids from LC-MS datasets
Source: Sci Rep. 2017 Nov 9;7:15138. doi: 10.1038/s41598-017-15363-z (PMC5680299; doi:10.1038/s41598-017-15363-z)
Supplement: Supplementary file 1 — Supplementary Information [file 41598_2017_15363_MOESM1_ESM.pdf]

LPPtiger software for lipidome specific prediction and identification of oxidized phospholipids from LC-MS datasets

Zhixu Ni[1,2], Georgia Angelidou[1,2], Ralf Hoffmann[1,2], Maria Fedorova[1,2,*].

[1]Institute of Bioanalytical Chemistry, Faculty of Chemistry and Mineralogy and [2]Center for Biotechnology and Biomedicine, Universität Leipzig, Deutscher Platz 5, 04103 Leipzig, Germany.

Corresponding Author:

Dr Maria Fedorova, Institut für Bioanalytische Chemie, Biotechnologisch-Biomedizinisches Zentrum, Deutscher Platz 5, 04103 Leipzig, Germany. E-mail: maria.fedorova@bbz.uni-leipzig.de.

Supplementary Information

Supplementary Figures

Supplementary Figure S1: The default configuration of *in silico* oxidation products derived from one C=C double bond unit.

Supplementary Figure S2: The illustration of LPPtiger *in silico* fragmentation and fingerprint matching algorithms exemplified for PE(16:0/18:2[2xDB,1OH]).

Supplementary Figure S3: The screenshot of LPPtiger graphic user interface (GUI) on Windows 10 64bit.

Supplementary Figure S4: The parallel processing workflow of LPPtiger represented by three-level architecture.

Supplementary Figure S5: Screenshot of LPPtiger HTML output report file opened in Mozilla Firefox browser.

Supplementary Figure S6: Effect of MS resolving power on detection of predicted isomeric and isobaric oxPLs species within four *m/z* units (*m/z* range 814.00 - 818.00).

Supplementary Figure S7: LPPtiger specificity towards interclass isomeric LPPs.

Supplementary Figure S8: Principal component analysis of differentially regulated PL-LPPs in SIN-1- treated cardiomyocytes, identified by LPPtiger and relatively quantified using Progenesis QI.

Supplementary Figure S9: Hierarchical clustering analysis of differentially regulated PL-LPPs in SIN-1-treated cardiomyocytes, identified by LPPtiger and relatively quantified using Progenesis QI.

Supplementary Figure S10: Examples of the nomenclature for PL-LPPs used by LPPtiger.

Supplementary Figure S11: LPP descriptors provided for each LPPtiger-generated entry in .sdf structure library exemplified for PC(18:0/18:1[1xDB,1xKETO]).

Supplementary Figure S12: The processing time benchmark of LPPtiger multicore performance.

Supplementary Tables

Supplementary Table 1: List of references to fatty acid oxidation related publications used to reconstruct SBML networks.

Supplementary Table 2: Summarized MS/MS fragmentation patterns of PL-bound LPPs obtained by *in vitro* oxidation of PC, PE, PA, PG, and PS standards and analyzed with ESI-QTOF instrument.

Supplementary Table 3: Example of LPPtiger output table for PC-, PE-, PS-, PG-, and PA-bound LPPs identified in 70min_SIN dataset.

Supplementary Table 4: The cross-validation between LPPtiger predicted and measured *in vitro* oxidized PL-LPPs from different PL classes.

Supplementary Table 5: Summary of PL-LPPs identified by LPPtiger in lipids extracts from cardiomyocytes treated with SIN-1. Corresponding lipids used for quantification are marked (T in dark green cell).

Supplementary Table 6: Summary of relative quantification for PL-LPPs identified by LPPtiger in extracts from SIN-1 treated cardiomyocytes performed by Progenesis QI. Results are expressed relative to control values. Only significantly regulated PL-LPPs (ANOVA $p \leq 0.05$) are shown.

Supplementary Table 7: Configuration files used to define fatty acid residues, modification types, PL-LPP class specific product and neutral loss ions, and instrument dependent weight factors used by LPPtiger for PL-LPP identification.

Supplementary Files:

Supplementary Files 1-10: SBML files of fatty acid oxidation networks for linoleic (9,12 18:2; **File 1**), alpha-linolenic (9,12,15 18:3; **File 2**), eicosapentaenoic n-3 (5,8,11,14,17 20:5; **File 3**),

docosapentaenoic n-3 (7,10,13,16,19 22:5; **File 4**), docosahexaenoic n-3 (4,7,10,13,16,19 22:6; **File 5**), gamma-linolenic n-6 (6,9,12 18:3; **File 6**), dihomo-gamma-linolenic n-6 (8,11,14 20:3; **File 7**), arachidonic n-6 (5,8,11,14 20:4; **File 8**), docosatetraenoic n-6 (7,10,13,16 22:4; **File 9**) and docosapentaenoic n-6 (4,7,10.13,16 22:5; **File 10**) reconstructed using CellDesigner based on the literature meta-study.

Supplementary File 11: LPPtiger User Guide.

Supplementary File 12: Examples of LPPtiger generated six-panel image outputs and corresponding original CID tandem mass spectra of LPPs identified in *in vitro* oxidized PL standards (Examples 1-12) and cardiomyocyte lipidomes (Examples 13-20).

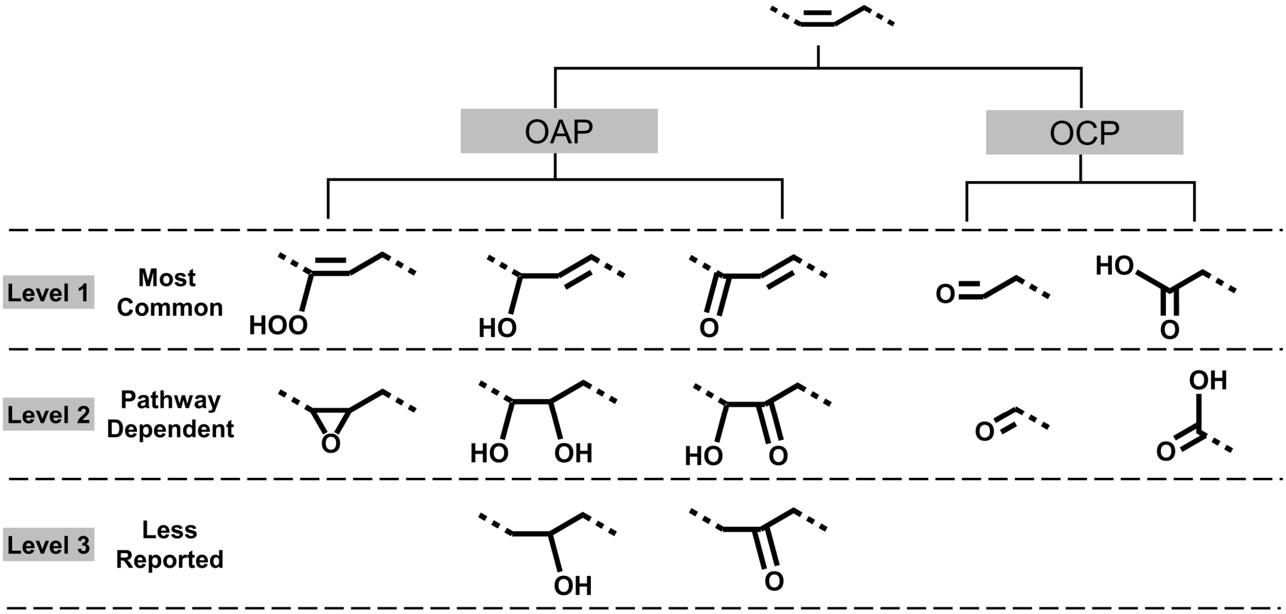

Figure S1. The default configuration of *in silico* oxidation products derived from one C=C double bond unit. The oxidation modifications were divided into three levels based on the literature survey, and can be expanded and altered depending on specific requirements.

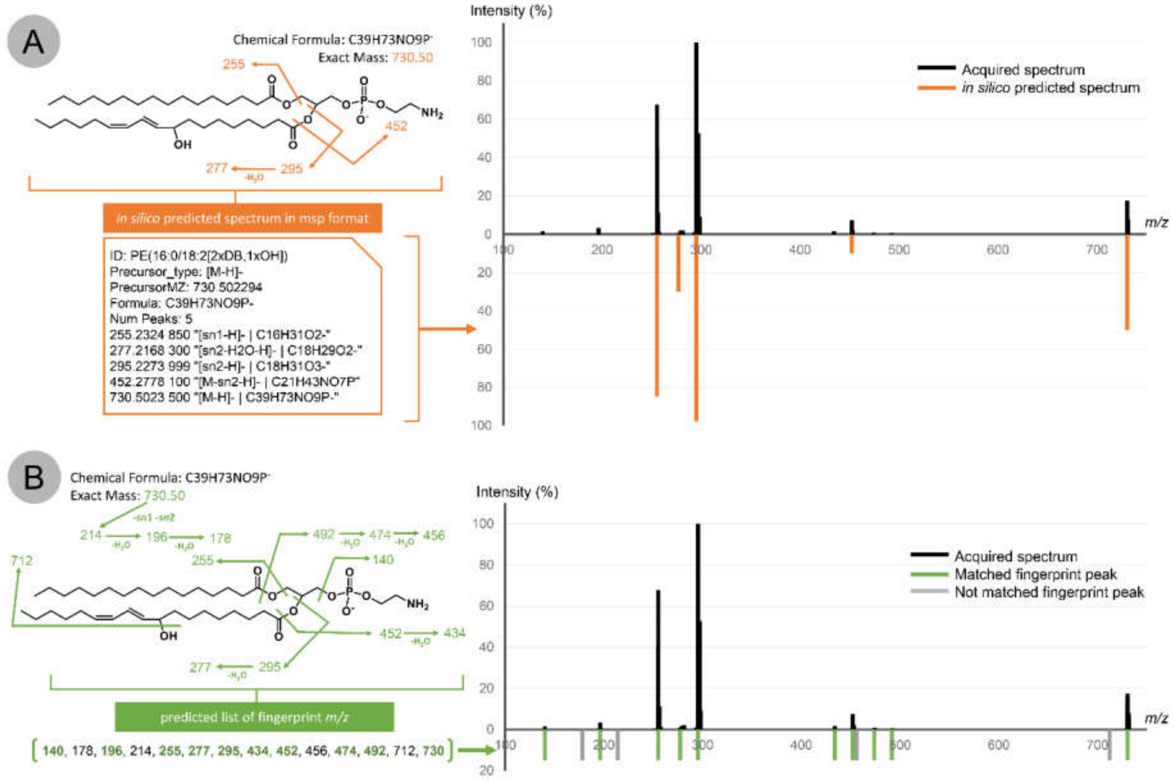

Figure S2. The illustration of LPPtiger *in silico* fragmentation and fingerprint matching algorithms exemplified for PE(16:0/18:2[2xDB,1OH]). (**A**) LPPtiger predicted LPP is *in silico* fragmented into the main types of the product ions including fatty acid anions and corresponding neutral losses, which are then assigned with a relative intensity values according to the summarized fragmentation patterns (in this example, fragmentation pattern of PE OAP products with one hydroxy group). Generated fragment/relative intensity pairs are saved as predicted spectra (orange trace) in .msp format and used to match measured spectra (black) to generate the Spectra Similarity Score. (**B**) The Fingerprint Fragmentation algorithm perform fragmentation at all known sites to generate a *m/z* list of all possible fragments without corresponding relative intensity values. Predicted fragments include fatty acid anions (e.g. 295.227 and 255.232), corresponding neutral losses (e.g. 492.273, 452.278, and 214.048), head group specific ions (e.g. 140.011, 196.037, and 178.027) and possible water losses (e.g. 712.492, 474.262, 456.251, 434.267, and 277.217). Measured spectra (black) are then matched to calculated fingerprint *m/z* list. Matched and unmatched *m/*z values are illustrated in green and grey, respectively, and used to calculate the Fingerprint Score. Visualization of *in silico* fragmentation and fingerprint matching for each identified LPP is illustrated by the six-panel image outputs provided in LPPtiger html reports.

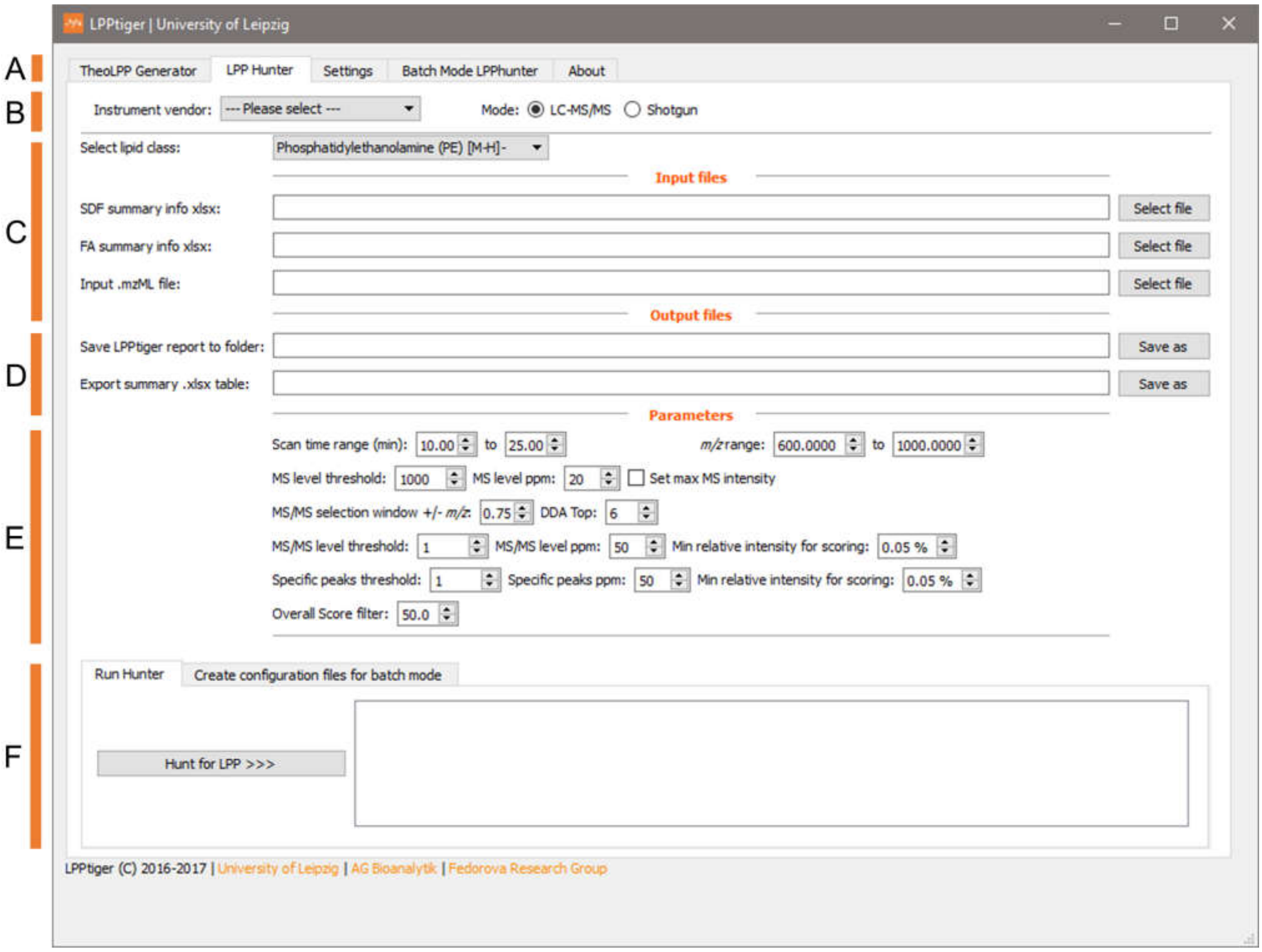

Figure S3. The screenshot of LPPtiger graphic user interface (GUI) on Windows 10 64bit. Section A – The navigation tabs. The LPPHunter tab is selected here as an example. Users are advised to read the "About LPPtiger" page, agree with the license disclaimer, and read the user guide to adjust the configuration settings in the "Settings" tab before beginning the data processing. **Section B** – Panel to choose for the instrument vendor (currently available for .mzML converted from Waters and Thermo Fisher Scientific .raw files) and acquisition method (LC-MS/MS or shotgun). **Section C** – Panel to select PL class and provide input files. **Section D** – Output files selection panel. **Section E** – Parameters selection panel. **Section F** – Start and status area. When "Run Hunter" tab is selected, user can execute the computational step. Summary information will be displayed after the process is finished. If the "Create configuration files for batch mode" tab is selected, the user can save all parameters to a configuration file which can be used in batch mode. Detailed information on GUI navigation is provided in the User Guide.

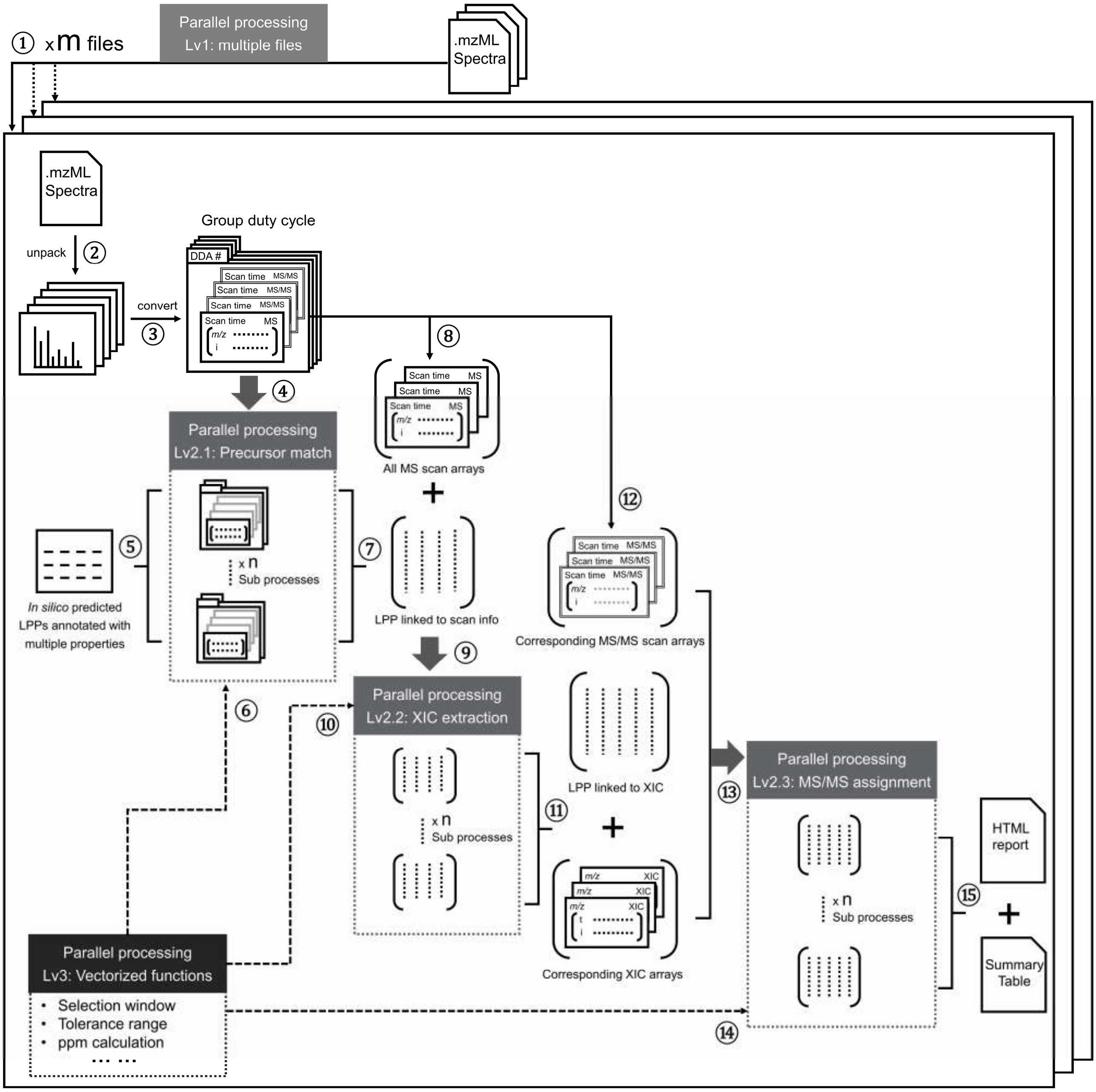

Figure S4. The parallel processing workflow of LPPtiger represented by three-level architecture. ① Multiple .mzML spectra can be processed by LPPtiger in parallel (Level 1). ② All MS and MS/MS scans are unpacked and extracted from the .mzML file by scan time. ③ Extracted scans are converted into arrays of m/z and corresponding intensities, and further grouped by their initial DDA duty cycle. ④ All MS/MS precursor m/z are taken for a precursor match using the summary information of in silico generated LPPs (⑤) to generate a list of LPPs labelled with corresponding spectra information (⑦). In step ④, the overall spectra library was divided into several subgroups (n = number of sub processes defined by user) to perform

precursor match in parallel, which is the first parallel processing step for an individual .mzML spectra file (Level 2.1). ⑥ Since the calculation of certain parameters, e.g., selection window, tolerance range, mass accuracy error (in ppm), need to be performed for a vast number of data, a third level of parallelization using vectorized functions is implemented to accelerate computation speed. ⑨ The pre-identified LPPs list (⑦) is split into n sub-lists for the parallel extraction of XIC using all MS spectra arrays (⑧) and accelerated by Level 3 parallel processing (⑩). Each extracted XICs are stored as an array of retention time and signal intensities, and linked to the pre-identified LPP list (⑪). ⑬ XIC extraction results and all MS/MS arrays (⑫) are used to perform MS/MS spectra assignment and scoring in parallel with acceleration (⑭) and generate final HTML report with integrated images and overall summary table (⑮).

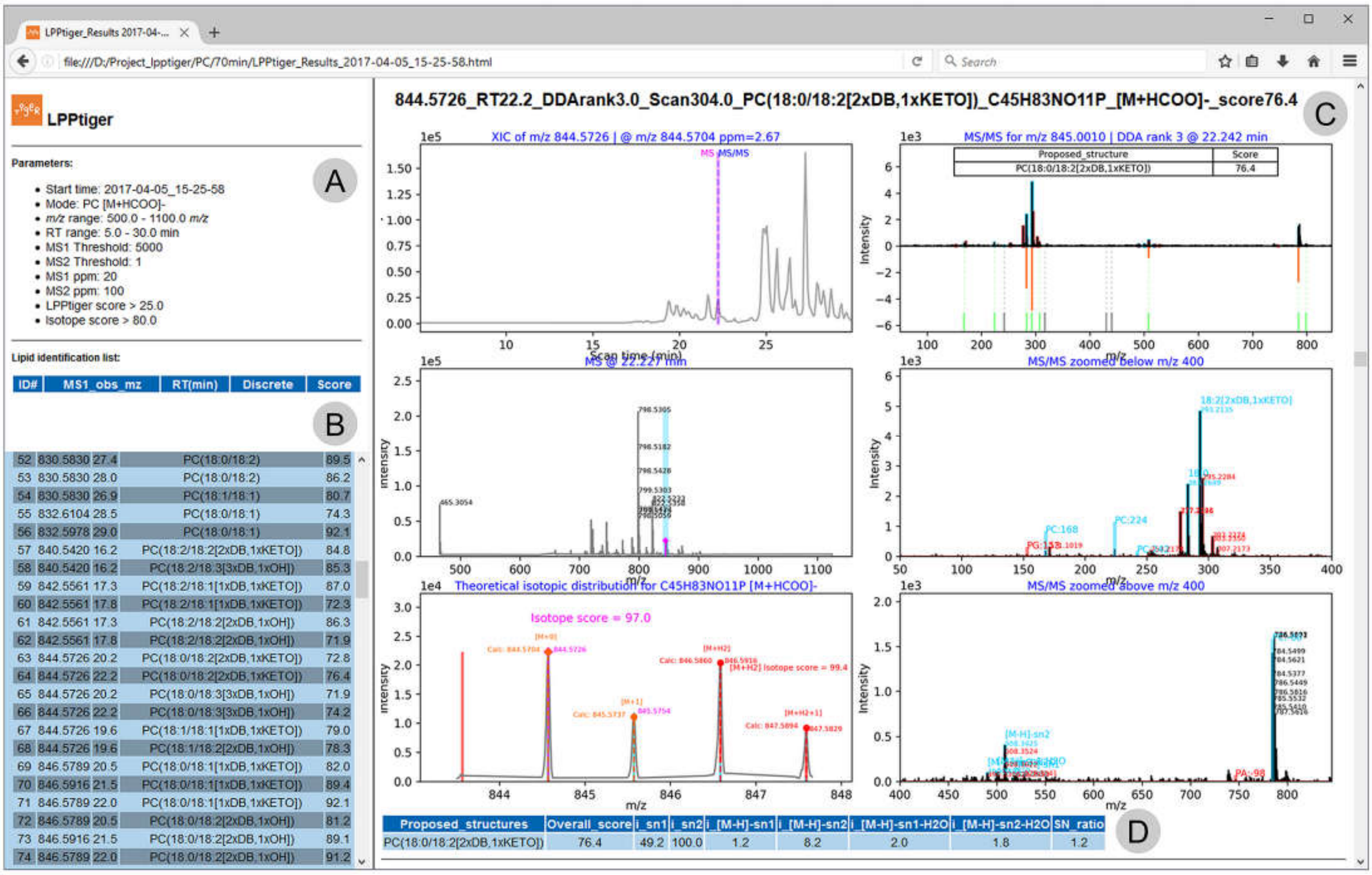

Figure S5. Screenshot of LPPtiger HTML output report file opened in Mozilla Firefox browser. The LPPtiger report provides a simple and organized solution to review, store and exchange the assigned outputs. The rich information .html report file from LPPtiger has four major parts: **A** - the main parameters used for identification, **B** - the overall identification table, **C** - the output images, and **D** - the table with the relative intensities of product ions for each identified LPP. Users can navigate between output images by clicking on the corresponding entry in the identification table. The original image can be accessed by clicking on the six-panel image (C).

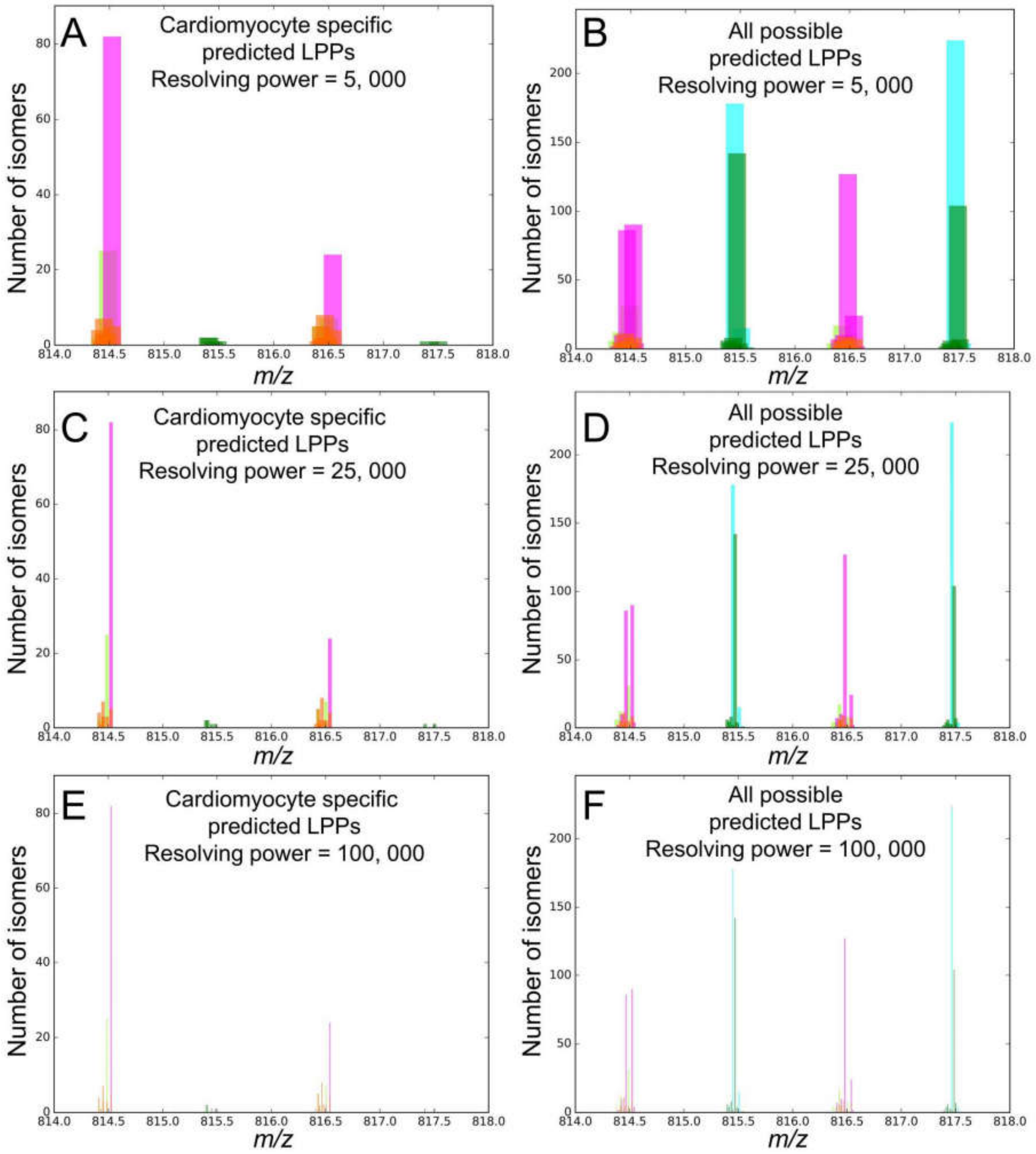

Figure S6. Effect of MS resolving power on detection of predicted isomeric and isobaric oxPLs species within four *m/z* units (*m/z* range 814.00 - 818.00) for cardiomyocyte (A, C, and E) and total (B, D, and F) predicted oxLipidomes. Higher MS resolving power will allow to distinguish more isobaric species. However, a large number of isomers will still not be resolved on MS1 level. Isomers resolution would require optimized orthogonal separation techniques (LC and/or ion mobility) as well as tandem MS analysis. Colour code: PA-LPPs (light green), PC-LPPs (magenta), PE-LPPs (cyan), PG-LPPs (dark green), and PS-LPPs (orange).

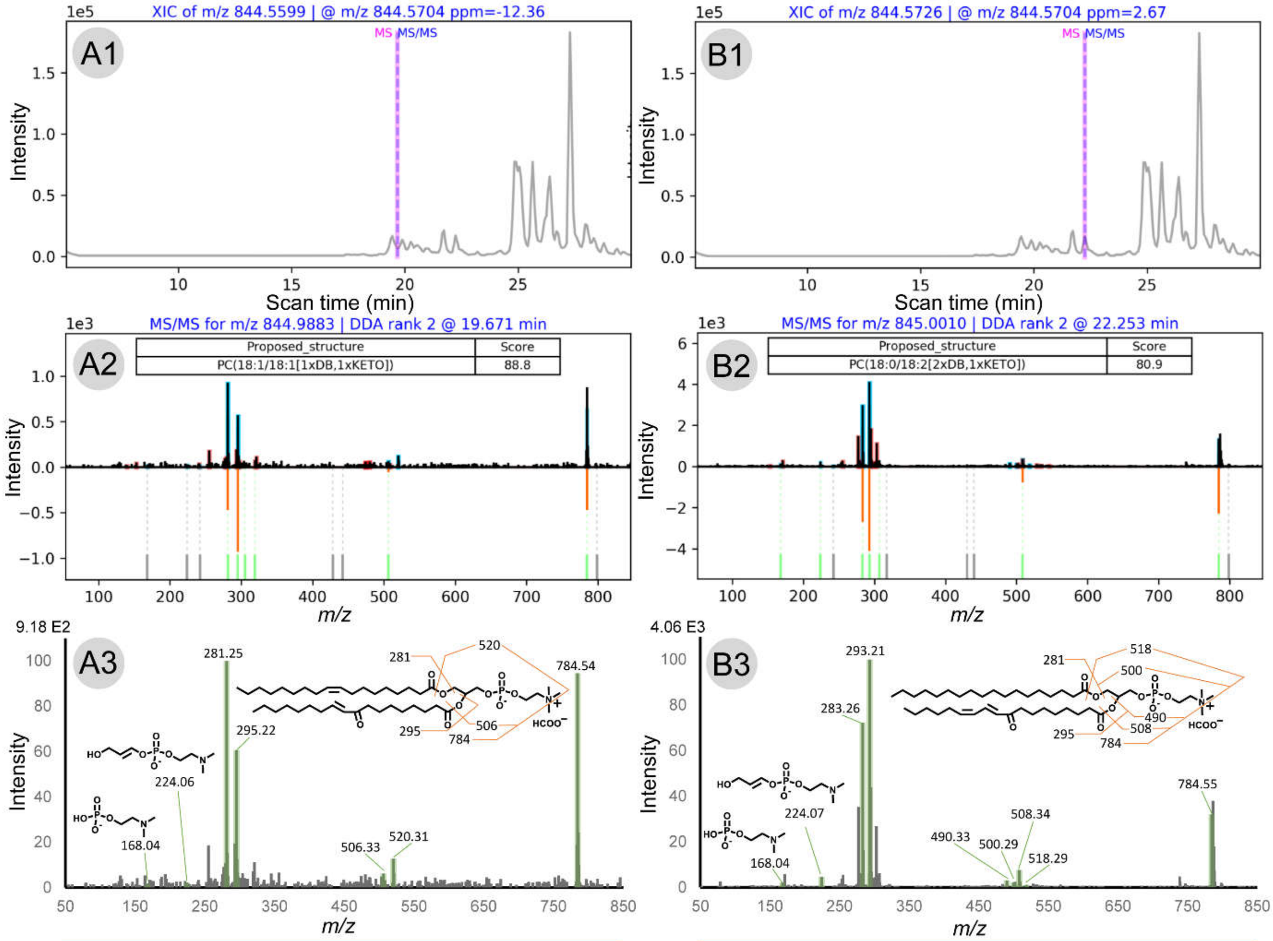

m/z	i	%	*m/z*	i	%
281.25	9.18E+02	100.0%	319.23	6.50E+01	7.1%
784.54	8.68E+02	94.6%	297.24	6.10E+01	6.6%
295.22	5.57E+02	60.7%	356.48	6.00E+01	6.5%
785.54	2.23E+02	24.3%	304.23	5.80E+01	6.3%
293.21	1.79E+02	19.5%	521.29	5.80E+01	6.3%
783.52	1.73E+02	18.8%	278.22	5.70E+01	6.2%
255.23	1.71E+02	18.6%	345.24	5.70E+01	6.2%
282.25	1.41E+02	15.4%	506.33	5.70E+01	6.2%
520.31	1.17E+02	12.7%	193.13	5.50E+01	6.0%
296.22	1.15E+02	12.5%	275.21	5.50E+01	6.0%
321.24	1.03E+02	11.2%	480.29	5.30E+01	5.8%
279.23	1.00E+02	10.9%	...	...	...
786.54	9.30E+01	10.1%	224.06	2.10E+01	2.3%
294.21	8.80E+01	9.6%	...	...	...
277.22	8.30E+01	9.0%	168.04	1.50E+01	1.6%

m/z	i	%	*m/z*	i	%
293.21	4.06E+03	100.0%	307.22	2.50E+02	6.2%
283.26	2.93E+03	72.2%	293.19	2.42E+02	6.0%
295.23	1.78E+03	43.8%	171.10	2.36E+02	5.8%
786.56	1.54E+03	38.0%	255.23	2.24E+02	5.5%
277.22	1.43E+03	35.3%	304.23	2.24E+02	5.5%
784.55	1.30E+03	32.0%	738.50	1.93E+02	4.8%
303.24	1.09E+03	26.8%	224.07	1.87E+02	4.6%
294.22	9.42E+02	23.2%	...	...	...
787.56	7.25E+02	17.9%	490.33	1.25E+02	3.1%
785.57	7.14E+02	17.6%	...	...	...
284.27	6.67E+02	16.4%	500.29	9.20E+01	2.3%
508.34	3.10E+02	7.6%	...	...	...
788.52	2.97E+02	7.3%	168.04	8.40E+01	2.1%
296.23	2.80E+02	6.9%	...	...	...
279.24	2.50E+02	6.2%	518.29	4.40E+01	1.1%

Figure S7. LPPtiger specificity towards interclass isomeric LPPs. Two isomers, with elemental composition C44H82NO9P and precursor *m/z* 844.5704, were separated by RPC (19.7 and 22.2 min) and identified by LPPtiger as two discrete species –PC(18:1/18:1[1xDB,1xKETO]) and PC(18:0/18:2[2xDB,1xKETO]), respectively. **A1/B1**, **A2/B2** – LPPtiger reconstructed XIC, and corresponding MS/MS spectrum used for the identification of PC(18:1/18:1[1xDB,1xKETO]) (**panels A**) and PC(18:0/18:2[2xDB,1xKETO]) (**panels B**). **A3/B3** – original manually assigned tandem mass spectra of isomeric LPPs with corresponding tables representing fragment ions *m/z* values, absolute and relative intensities used to assign PC(18:1/18:1[1xDB,1xKETO]) (**A3**) and PC(18:0/18:2[2xDB,1xKETO]) (**B3**) lipids.

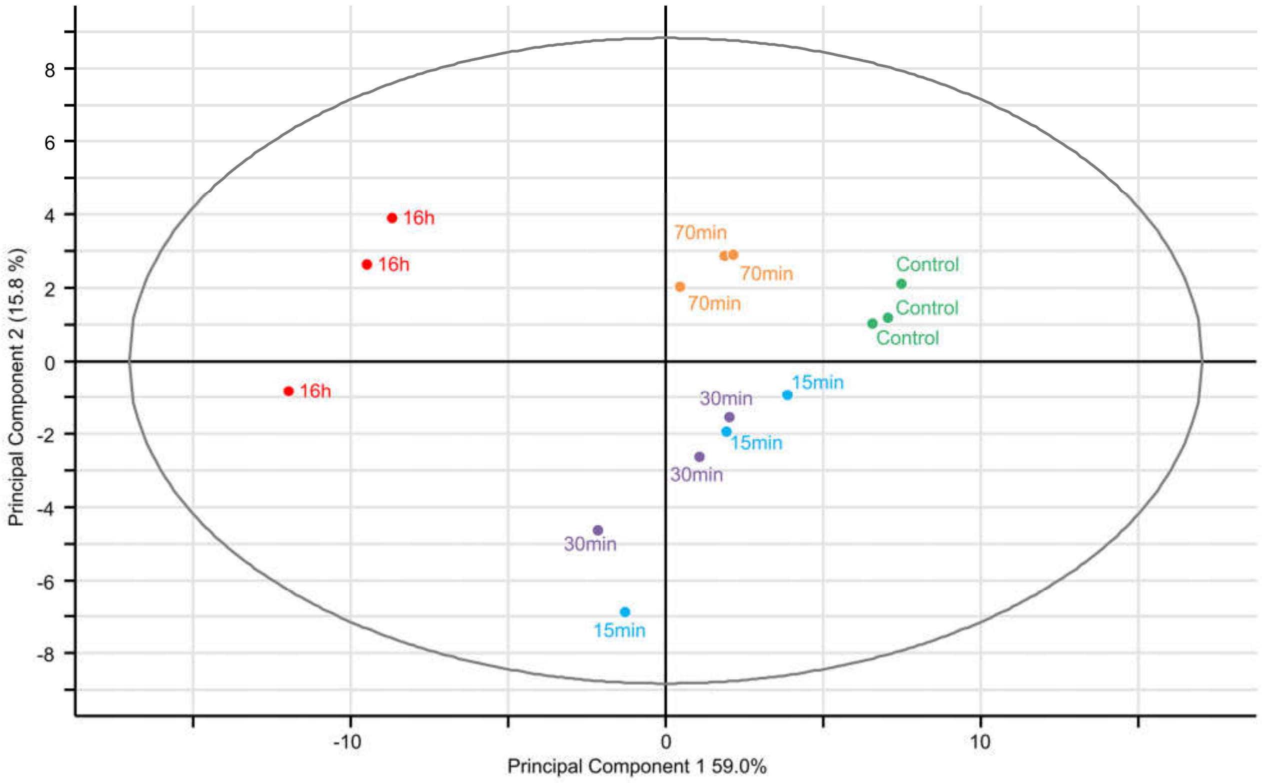

Figure S8. Principal component analysis of differentially regulated PL-LPPs in SIN-1- treated cardiomyocytes, identified by LPPtiger and relatively quantified using Progenesis QI.

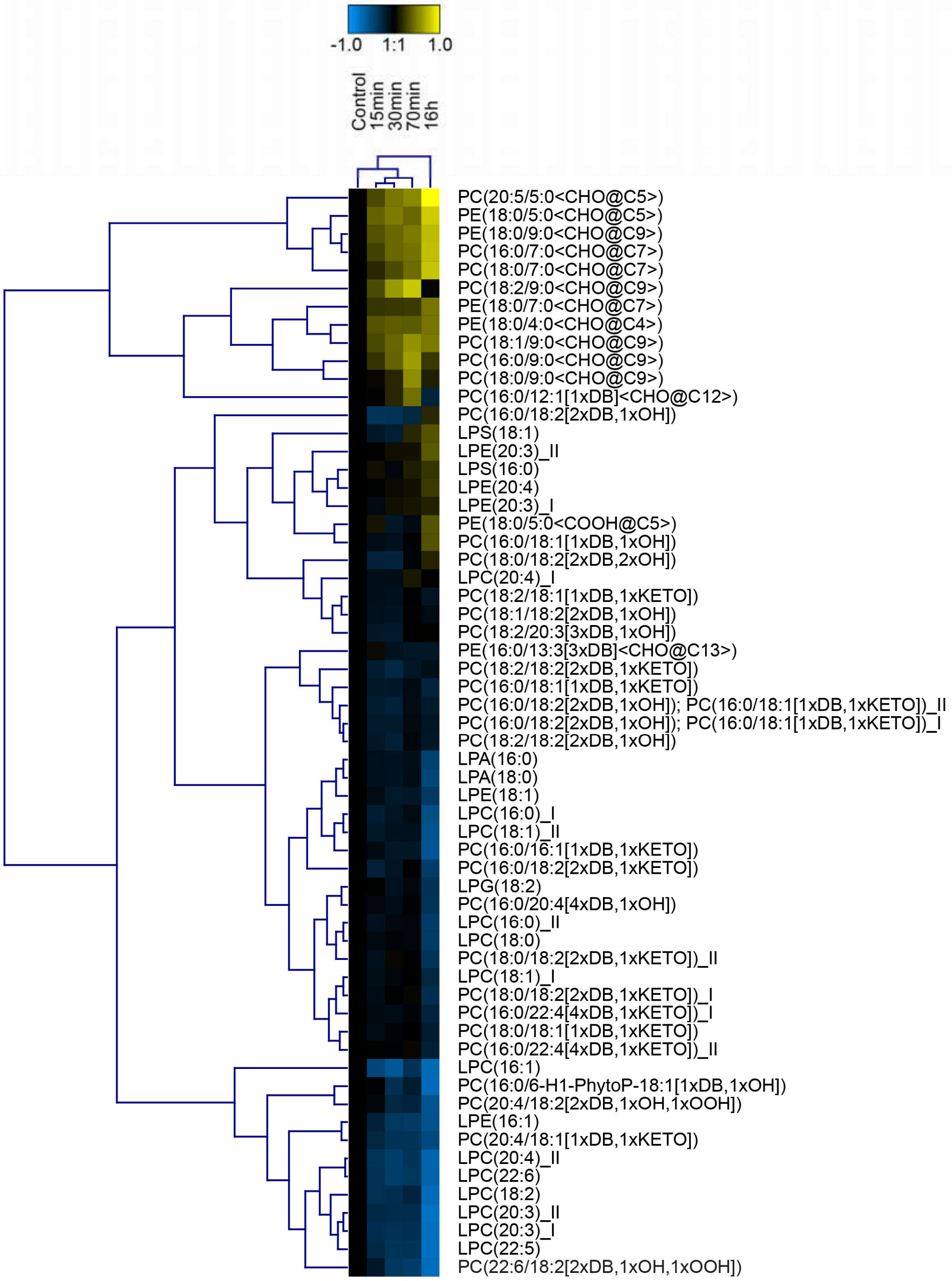

Figure S9. Hierarchical clustering analysis of differentially regulated PL-LPPs in SIN-1-treated cardiomyocytes, identified by LPPtiger and relatively quantified using Progenesis QI. Analysis was performed using Genesis software to generate the hierarchical clustering diagram.

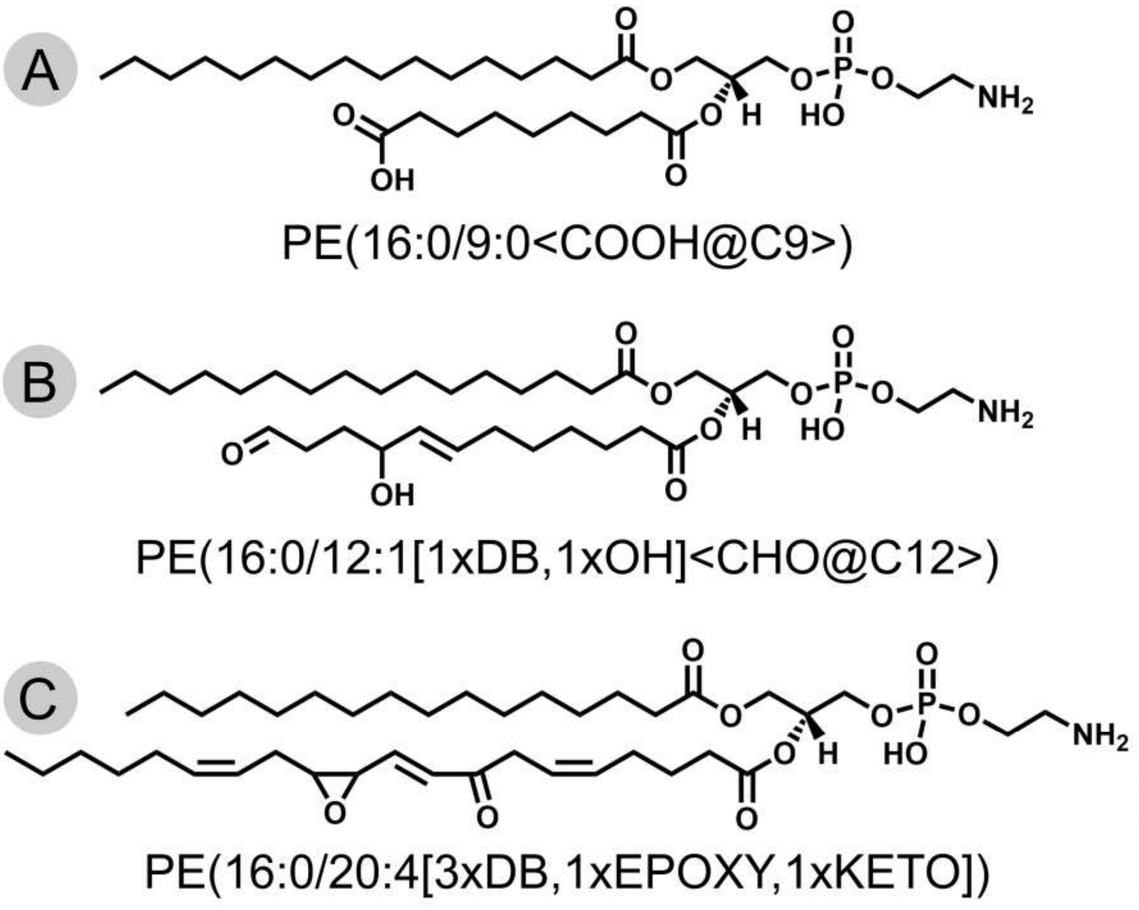

Figure S10. Examples of the nomenclature for PL-LPPs used by LPPtiger. A - PE(16:0/9:0<COOH@C9>), B - PE(16:0/12:1[1xDB,1xOH]<CHO@C12>), and C - PE(16:0/20:4[3xDB,1xEPOXY,1xKETO]).

LM_ID	PC(18:0/18:1[1xDB,1xKETO])
Structure	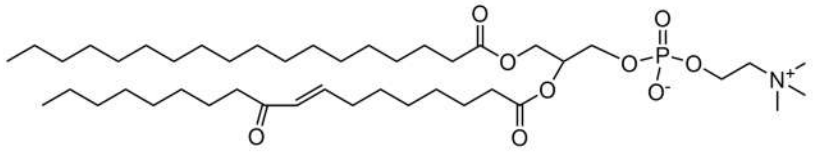
Formula	C44H84NO9P
NOMINAL_MASS	802.128
EXACT_MASS	801.588370
LPP_CLASS	PC
LPP_ORIGIN	PC(18:0/18:1)
LPP_SMILES	[O-]P(OCC[N+](C)(C)C)(OCC(OC(CCCCCCC(=O)/C=C/CCCCCCCC)=O)COC(CCCCCCCCCCCCCCCCC)=O)=O
PRECURSOR_JSON	{"[M+HCOO]-": ["C45H85NO11P-", 846.586024]}
SN_JSON	{"SN1": "UNMOD", "SN2": "OAP"}
SN1_ABBR	18:0
SN1_FORMULA	C18H36O2
SN1_SMILES	OC(CCCCCCCCCCCCCCCCC)=O
SN1_JSON	{"C": 18, "DB": 0, "CHO": 0, "EPOXY": 0, "OAP": 0, "OCP": 0, "COOH": 0, "KETO": 0, "OH": 0, "OOH": 0, "LINK_TYPE": ""}
SN2_ABBR	18:1[1xDB,1xKETO]
SN2_FORMULA	C18H32O3
SN2_SMILES	OC(CCCCCCC(=O)/C=C/CCCCCCCC)=O
SN2_JSON	{"C": 18, "DB": 1, "CHO": 0, "EPOXY": 0, "OAP": 1, "OCP": 0, "COOH": 0, "KETO": 1, "OH": 0, "OOH": 0, "LINK_TYPE": ""}
MSP_JSON	{"[M-CH3]-": {"i": 500, "formula": "C43H81NO9P-", "mz": 786.5649}, "[sn2-H]-": {"i": 999, "formula": "C18H31O3-", "mz": 295.2273}, "[sn1-H]-": {"i": 500, "formula": "C18H35O2-", "mz": 283.2637}, "[M-CH3-sn2]-": {"i": 50, "formula": "C25H51NO7P-", "mz": 508.3403}, "[M-H]-": {"i": 0, "formula": "C44H83NO9P-", "mz": 800.5805}}
FINGERPRINT	[168.046, 224.069, 242.079, 283.264, 295.227, 307.264, 319.227, 430.309, 442.272, 508.34, 786.565, 800.581]

Figure S11. LPP descriptors provided for each LPPtiger-generated entry in .sdf structure library exemplified for PC(18:0/18:1[1xDB,1xKETO]). Annotations can be divided into four main categories – (i) information about the whole PL-LPP structure (LM_ID, Formula, NOMINAL_MASS, EXACT_MASS, LPP_CLASS, LPP_ORIGIN, LPP_SMILES, PRECURSOR_JSON, and SN_JSON), (ii) sn-1 information (SN1_ABBR, SN1_FORMULA, SN1_SMILES, and SN1_JSON), (iii) sn-2 information (SN2_ABBR, SN2_FORMULA, SN2_SMILES, and SN2_JSON), and (iv) in-silico fragmentation information (MSP_JSON and FINGERPRINT). All fields end with "_JSON" can be parsed into key-value pairs.

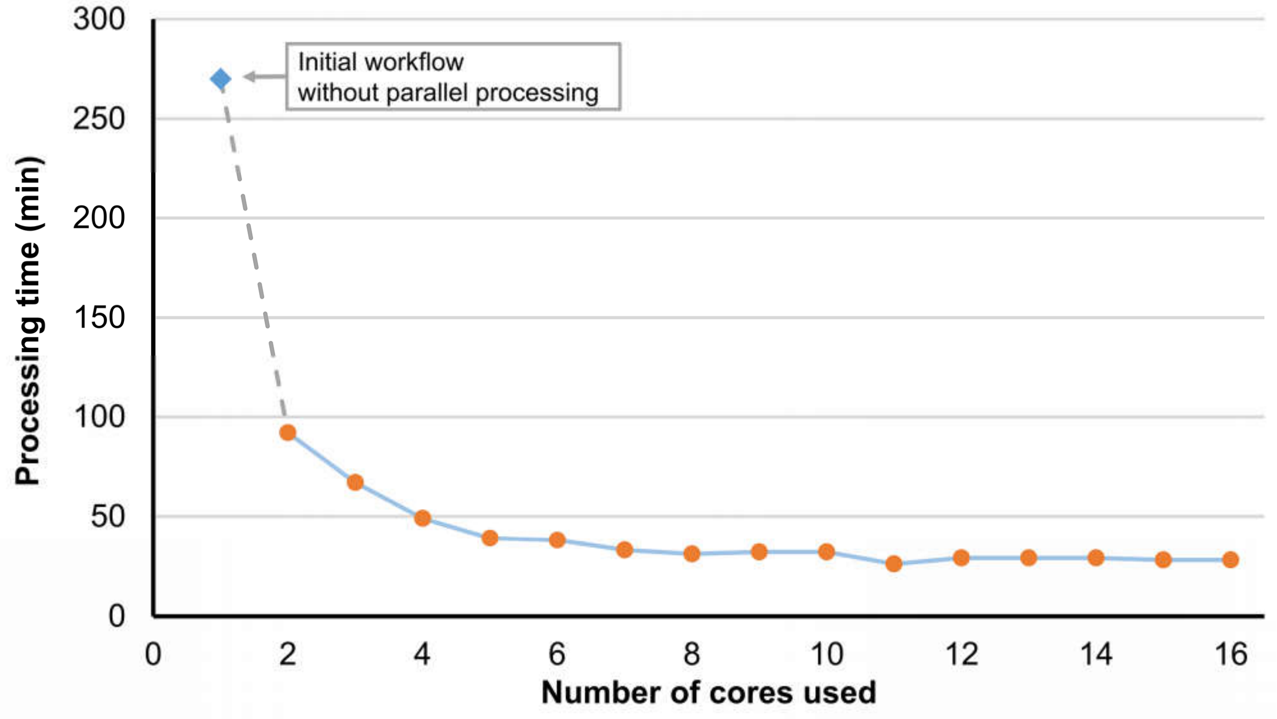

Figure S12. The processing time benchmark of LPPtiger multicore performance. A workstation configured with a 3 GHz CPU equipped with eight cores and hyper threading (16 threads in total) together with 32 GB of RAM was selected as the platform to evaluate the optimal parallel processing configurations using a sample dataset (250 MB). The processing time is significantly reduced as more threads are in use. However, the performance improvement above eight threads was found to be beneficial. Based on the benchmark results, the optimal settings for this particular workstation can be configured to run three files in parallel with five threads assigned to each file.
