# Supplementary material for: LPPtiger software for lipidome-specific prediction and identification of oxidized phospholipids from LC-MS datasets
Source: Sci Rep. 2017 Nov 9;7:15138. doi: 10.1038/s41598-017-15363-z (PMC5680299; doi:10.1038/s41598-017-15363-z)

**
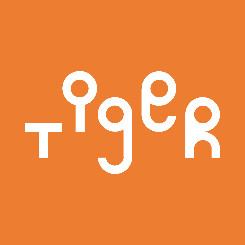
**

**User Guide**

**To**


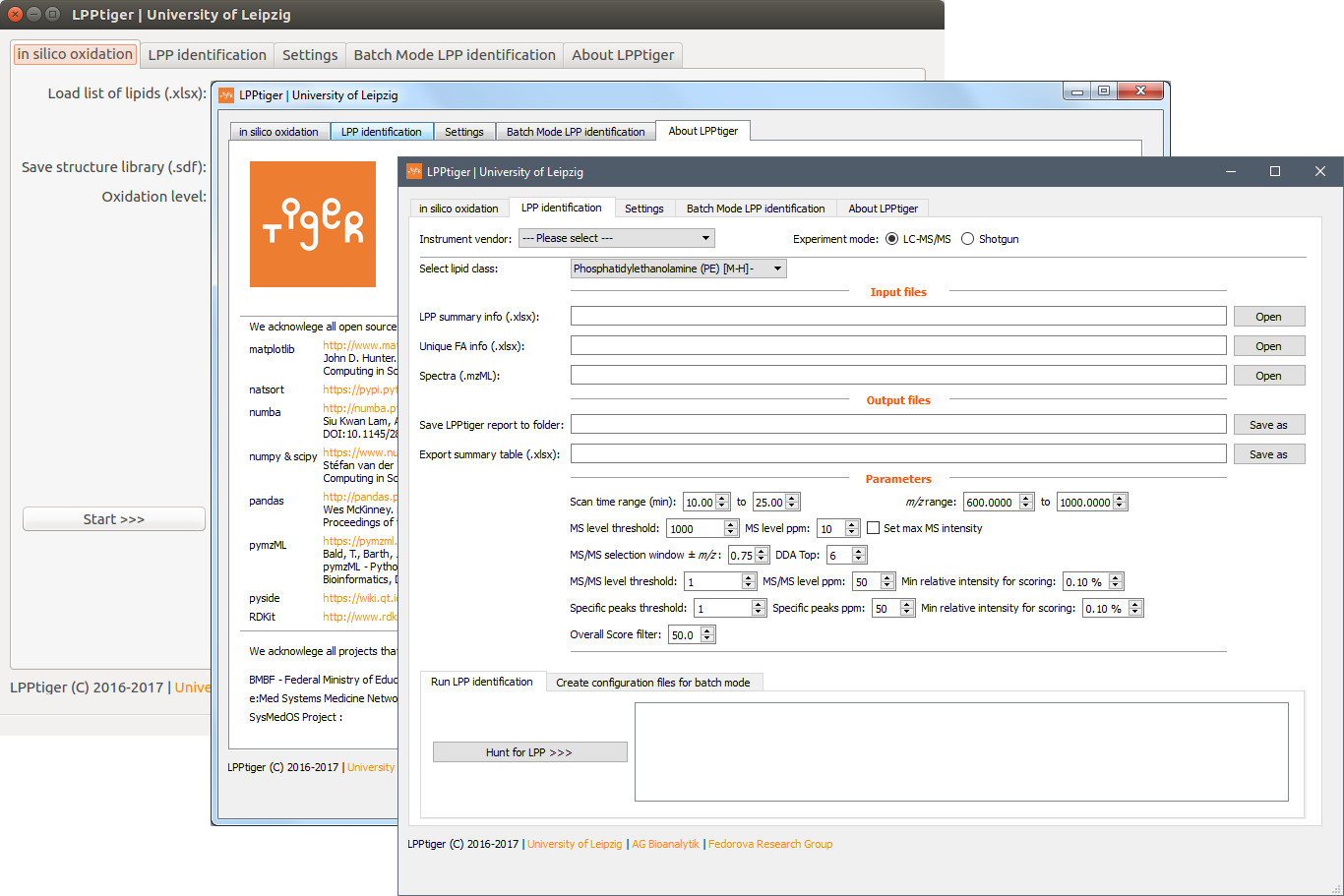
**LPPtiger**

For LPPtiger Beta version 31^th^, May, 2017

User guide version 19^th^, June, 2017


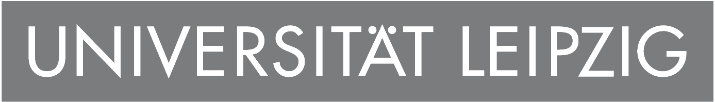

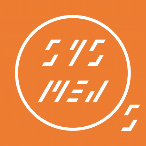


Table of Contents

[1. Introduction 1](#__RefHeading___Toc486428178)

[2. License 1](#__RefHeading___Toc486428179)

[3. Download and install LPPtiger 1](#__RefHeading___Toc486428180)

[4. Data conversion to .mzML 4](#__RefHeading___Toc486428181)

[4.1 Convert raw data to .mzML 5](#__RefHeading___Toc486428184)

[4.2 Check converted .mzML file by ProteoWizard SeeMS tool 6](#__RefHeading___Toc486428185)

[5 LPPtiger workflow overview 6](#__RefHeading___Toc486428186)

[5.1 Before you start 7](#__RefHeading___Toc486428187)

[5.2 Step I: in silico oxidation of selected phospholipids 11](#__RefHeading___Toc486428188)

[5.3 Step II: Hunt for LPPs 16](#__RefHeading___Toc486428189)

[5.4 Step III batch mode 19](#__RefHeading___Toc486428190)

[6 LPPtiger output 21](#__RefHeading___Toc486428191)

[7 Configuration files 24](#__RefHeading___Toc486428192)

# Introduction

LPPtiger is an open-source software for prediction and identification of lipid peroxidation products (LPPs) from data-dependent shotgun and LC-MS/MS data. LPPtiger main features include:

- Prediction of specific oxLipidome from native lipidome using novel *in silico* oxidation algorithm;
- Five-criteria scoring algorithm for LPP identification;
- Unique interactive HTML output with integrated annotated images for fast assessment of identification results;
- Fast and easy customizable workflows for different PL modifications;
- Available for Waters and ThermoFisher .raw files;
- User-friendly graphic interface;
- Native support for parallel processing;
- Cross platform application (Windows and Linux);
- Free and open-source;
- Rapid and transparent source code development managed by online public repository.

# License

LPPtiger is dual-licensed. For academic and non-commercial use – apply GPLv2 License (<https://www.gnu.org/licenses/old-licenses/gpl-2.0.en.html>). For commercial use please contact the SysMedOS team by email. Please cite our publication in an appropriate form.

# Download and install LPPtiger

There are two versions of LPPtiger available - Windows executable and source code version. General information and installation instructions can be found here - <https://bitbucket.org/SysMedOs/lpptiger>

- Windows executable version:
  - Executable versions are provided for Windows 7, 8 and 10 64bit system only.
  - Please read instructions how to download Windows version here [https://bitbucket.org/SysMedOs/lpptiger_exe](../../../../D:%5CPhDbbz%5CPublications%5CLPPtigerPaper%5C%09https:%5Cbitbucket.org%5CSysMedOs%5Clpptiger_exe)
  - Download link  [https://bitbucket.org/SysMedOs/lpptiger_exe/downloads/](../%20https://bitbucket.org/SysMedOs/lpptiger_exe/downloads/)
- Source code version:
  - For developers or other platform users (Linux, Windows server editions), LPPtiger source code is available. Please read the instructions how to download the source code version here <https://bitbucket.org/SysMedOs/lpptiger>
- Other files provided with LPPtiger:
  - - Test files to be used with this user guide includes sample dataset in .mzML format, templates and other sample outputs and can be downloaded here <https://bitbucket.org/SysMedOs/lpptiger_exe/downloads/>
    - Updates of LPPtiger user guide. Please check the following link for the latest version of LPPtiger user guide <https://bitbucket.org/SysMedOs/lpptiger_exe/downloads/>
- Other software required to work with LPPtiger:
  - .mzML spectra converter and viewer - ProteoWizzard MSconvert and SeeMS (<http://proteowizard.sourceforge.net/>)
  - SDF structure library viewer. Please install an appropriate SDF file viewer based on your interests from:
- ChemAxon Instant JChem (cross platform, academic license available) - <https://www.chemaxon.com/products/instant-jchem-suite/instant-jchem/>
- Progenesis SDF Studio (Windows only, free to use) - <http://www.nonlinear.com/progenesis/sdf-studio/>

We recommend to use 7-zip program to unpack LPPtiger zip package (7-Zip is a free and open source software and can be downloaded from [http://www.7-zip.org](http://www.7-zip.org/)).


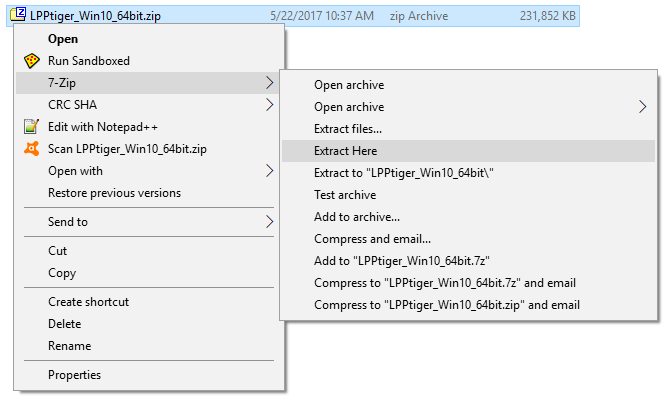


After the extraction, we recommend sorting the content of the folder using the “Group by” function.


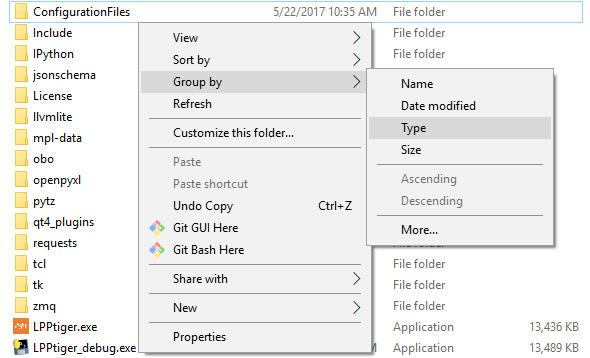


There are two excitable files named “LPPtiger.exe” and “LPPtiger_debug.exe” corresponding to the original LPPtiger program and a debug mode of LPPtiger, respectively. The debug mode will show the background information in a separated command line window connected to LPPtiger interface.


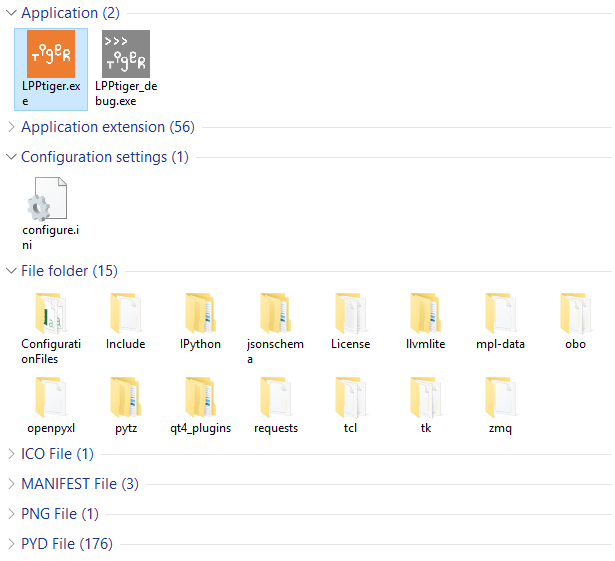


Default LPPtiger settings are saved in “configure.ini” file. Default configuration files are provided in the “ConfigurationFiles” folder. Licenses of LPPtiger dependencies are saved in the “License” folder.

**!!! General Notes**

- LPPtiger might be blocked by anti-virus software, e.g., Avast, Kaspersky, etc. Please provide permissions to run LPPtiger or add LPPtiger to the white list and try to launch it again.
- We recommend creating a shortcut of “LPPtiger.exe” to your desktop.

**How to uninstall LPPtiger?**

LPPtiger is a green software, which means you can simply delete the LPPtiger program folder (and shortcuts if there is any) to uninstall it.

# Data conversion to .mzML

LPPtiger is designed to work with .mzML files obtained from LC-MS/MS and shotgun data-dependent acquisition experiments. Original data should be converted to .mzML files using ProteoWizard MSconvert tool.

- Go to <http://proteowizard.sourceforge.net/downloads.shtml>
- Download the version suitable for your system
- Install ProteoWizard
- Open MSConvert from ProteoWizard folder

**!!! General Notes**

- The conversion of .mzML is the most critical step for LPPtiger workflow. The .mzML files provided in the LPPtiger test example were converted by ProteoWizard version 3.0.9134.
- The ProteoWizard version you download from the ProteoWizard website might be different.
- The .mzML file converted from specific instruments by different ProteoWizard version might be different, thus they might not be compatible with LPPtiger. Please find a suitable ProteoWizard version to convert your raw files and keep using the same ProteoWizard version for further analysis as long as not critical updates of ProteoWizard are necessary.
- If you failed to convert or the .mzML cannot be processed by LPPtiger, please read our wiki to understand the LPPtiger specific requirement of the .mzML file format. Please contact ProteoWizard team to get a proper version to convert your files.
- Please read LPPtiger wiki about essential data sections in the .mzML files at:

[https://bitbucket.org/SysMedOs/lpptiger/wiki/LPPtiger%20.mzML%20format%20requirements](https://bitbucket.org/SysMedOs/lpptiger/wiki/LPPtiger .mzML format requirements)

## Convert raw data to .mzML

To generate a **.mzML file** containing both MS survey scan and MS/MS information:

- Please create a new folder and save .mzML file to the new folder. (Saving mzML file to the previous location might overwrite previous converted files.)
- Input your data in .raw format
- Click “Add”
- Choose binary coding precision **“32 bit”** to minimize the file size
- Other parameters (e.g. ScanTime, mzWindow, Threshold) can be specified

| Options | Required parameters |
| --- | --- |
| Binary coding precision | 32-bit |
| Write index | True |
| Use zlib compression | True |
| TPP compatibility | True |
| Package in gzip | False |
| Use numpress options | False to all |
| MS level | Do not apply filter here (Important for Waters .raw files) |
| Options | Suggested parameters |
| Threshold peak filter | Absolute intensity |
| Subset 🡪 Scan Time | Scan time range in seconds |
| Subset 🡪 *m/z* Window | Do not apply any filter here |

## Check converted .mzML file by ProteoWizard SeeMS tool

It is very important to check your converted mzML files before starting to work with LPPtiger. The SeeMS tool in the ProteoWizard program folder provides an excellent way to view .mzML files.

Please check following parameters of converted .mzML files.

- The MS level range.
  - **For Waters files**, the MS-Level 1 corresponding to MS survey scan, MS-Level 2 corresponding to DDA rank 1, MS-Level 3 corresponding to DDA rank 2, thus DDA rank N experiment should have MS-Level N+1 converted.
- MS2 spectra *m/z* range
  - Please make sure that MS2 spectra contain *m/z* range of specific fragments and neutral losses necessary for the identification.
- Spectra intensity
  - Please check the intensity of structure representative signals. Adjust the “Absolute intensity” filter accordingly and convert again if necessary. Absolute intensity filter above 100 000 for MS2 is NOT recommended.
  - Please start your own data with a relatively low threshold to finish the workflow of LPPtiger. You can adjust these conversion settings carefully based on a spectra quality and from preliminary LPPtiger results.

# LPPtiger workflow overview

**!!! General Notes**

- Several .xlsx and .csv tables will be generated and used by LPPtiger. To avoid “comma vs dot” derived errors, please make sure that language on the computer is set to “US English” while working with LPPtiger.
- Note that during computational steps LPPtiger will display *“Not Responding”* message on the window title. The *in silico oxidation* process (level 2 and level 3) and identification steps might take very long time to run, during this time, LPPtiger GUI might freeze and do not respond to user actions, please be patient and wait until processing finishes. LPPtiger source code version users can monitor detailed information during the run. Windows executable version users can run the debug mode to monitor the background processes through the command line window.

To test LPPtiger we recommend you to use the provided sample data package available at [https://bitbucket.org/SysMedOs/lpptiger_exe/downloads/](../%20https://bitbucket.org/SysMedOs/lpptiger_exe/downloads/). The template file and identification output can be found in corresponding subfolders. We recommend constructing the same structure of sub-folders for your own projects.


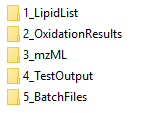


## Before you start

Configuration files containing a fatty acid white list, PL specific product/neutral loss signals, and weight factor tables should be uploaded to the **Settings** tab. Instructions how to modify/create user specific configuration files are provided in [Chapter 7](#_Configuration_files).

The default configuration files are provided in the “ConfigurationFiles” folder under LPPtiger root folder. Please select the files according to the following instructions and press “save above settings as default” button to save the settings.


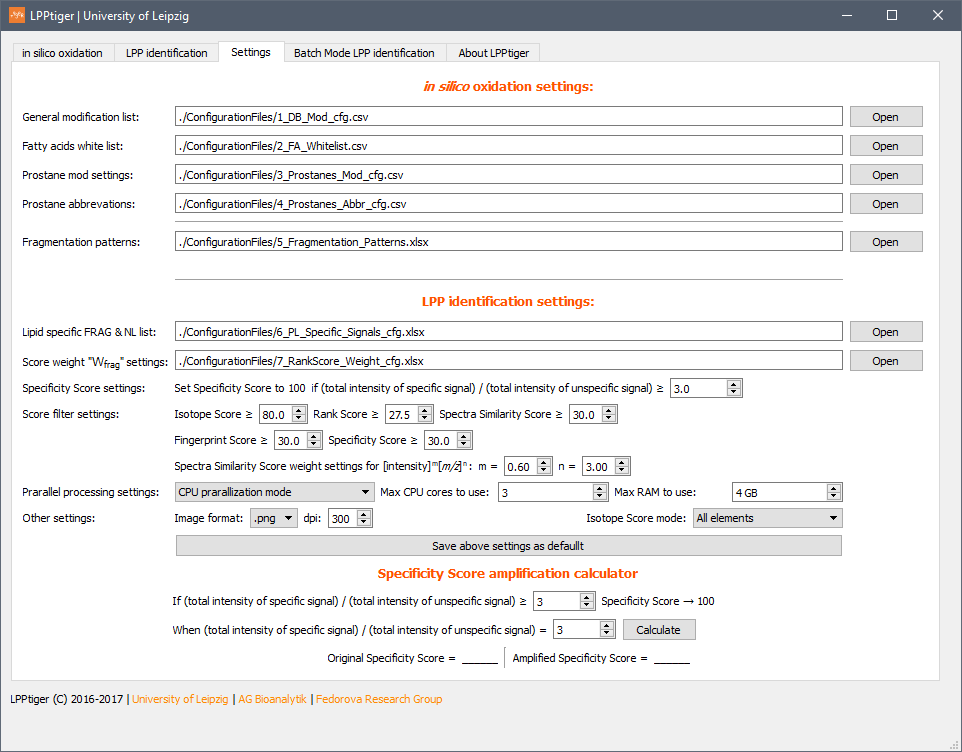


Please restart LPPtiger, and check the **Settings** tab to see if configure files are loaded correctly before you start to use LPPtiger. An example is provided below.


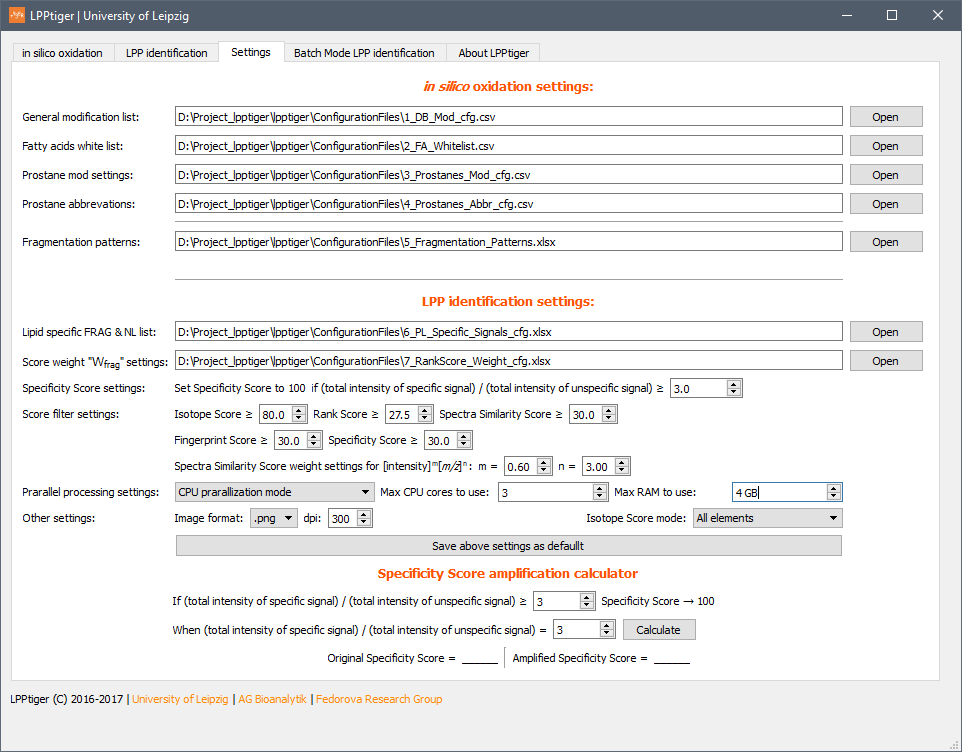


Please go to [Chapter 7](#_Configuration_files) for more detailed description of each configuration file.

**Parameters:**

- **Specificity score settings:**

Set the intensity ration threshold, which defines a filter intensity ratio for structure specific vs unspecific signals. LPPs with the ratio equal or higher than defined threshold will be assigned with 100 points for Specificity Score. This ratio is used to calculate the amplification factor of Specificity Score. We recommend to use intensity ration threshold of 5 (for Orbitrap data) or 3 (for Q-ToF data).

- **Score filter settings:**

**Score filter for individual scores: I**ndividual scores can be used to control the identification quality for all five scoring dimensions based on the characteristics of each dataset. The default values can be applied to most of the situations. However, specific adjustment to the instruments and sample types may reduce the number of false positives and avoid false negatives in the outputs.

**Spectra similarity score weight settings:** The spectra similarity score is calculated by modified signal to noise ratio (SNR) function with a user definable weight factor. With the help of this factor, user can define a specific ratio between the overall intensity of structure and unspecific specific signals. The default value will enhance the specificity score to 100 in case ratio of specific vs unspecific signals is 5 or above. A simple calculator is provided in the settings tab to help users to decide which ratio and corresponding weight factor are the optimal values for selected instrument and datasets.

- **Parallel processing settings:**

**Parallel processing mode:** LPPtiger uses CPU only for vectorized functions in parallel processing. An experimental GPU mode can be applied to Windows systems with CUDA compatible graphic cards. We recommend to use default CPU only mode in the current version.

**Number of CPU cores** defines the maximum number of sub processes that can be used to process one file. This parameter is usually suggested to correspond to the number of logical processors minus one, e.g., if you have a dual core CPU with hyper-threads, you get four logical processors and this parameter can be set to 3. We recommend to use maximum of five cores for a single file.

**Max RAM LPPtiger:** we recommend to use at least 4 GB RAM (maximum 10 GB) for typical LC-MS/MS .mzML file. Please reserve at least 2 GB RAM for the Windows systems, e.g., if you have 8 GB of RAM installed, this parameter can be set to 6 GB.

**# Note:** For Windows users, the number of CPU cores, number of logical processors, and installed RAM can be read out from the task manager.

- **Other settings:**

**Image Format:** available formats are “.png” and “.svg”.

**dpi:** we recommend to use 300 dpi or lower for preliminary research and 600 dpi for publication quality results.

**Isotope score mode:** Set to “all elements” to consider all isotopes. “Fast mode” considers only carbon isotopes.

**!!! General Notes**

- Save defined settings for **Spectra similarity score weight** and **Parallel processing mode** to default configuration file and restart LPPtiger to make changes effective.
- **ALL other parameters can be temporarily changed.** You can change these parameters instantly without saving the parameters to test different combinations. However, we recommend you to save an optimized parameters to default configuration file.
- **The default configuration file** is saved as “configure.ini” in the LPPtiger folder. We recommend to back up the optimized parameters to prevent unexpected changes.

Please make sure that you have read the information listed in the **About** tab and agree with our license.

We recommend you to check LPPtiger project page regularly for the latest updates.

Source code users and developers can find additional information about the python libraries that are used by LPPtiger.

## Step I: *in silico* oxidation of selected phospholipids

To start the *in silico* oxidation of phospholipids (PLs), a table of the selected PLs need to be prepared in a template and saved in “.xlsx” format. In this simplified example, input information for two PLs, PC(16:0/18:1) and PC(16:0/18:2), is saved as “PC_for_oxidation.xlsx”.


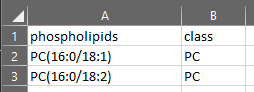


**!!! General Notes**

- If you want to specify the Omega series of the FA, you can write as follows. Please check the configuration file to ensure that you have the corresponding FA in the FA list.


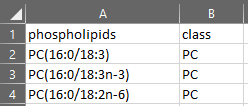


- For FA without specified Omega series LPPtiger will use the first FA configuration in the FA list configuration file.
- Please create a separate PL list for each class using one table sheet.
  - LPPtiger can load .xlsx files containing multiple sheets, so you can put different classes of PLs into different sheets.
  - Please do not change the default names of the excel sheets, and keep them as ascending order (e.g. Sheet1, Sheet2, Sheet 3 …).


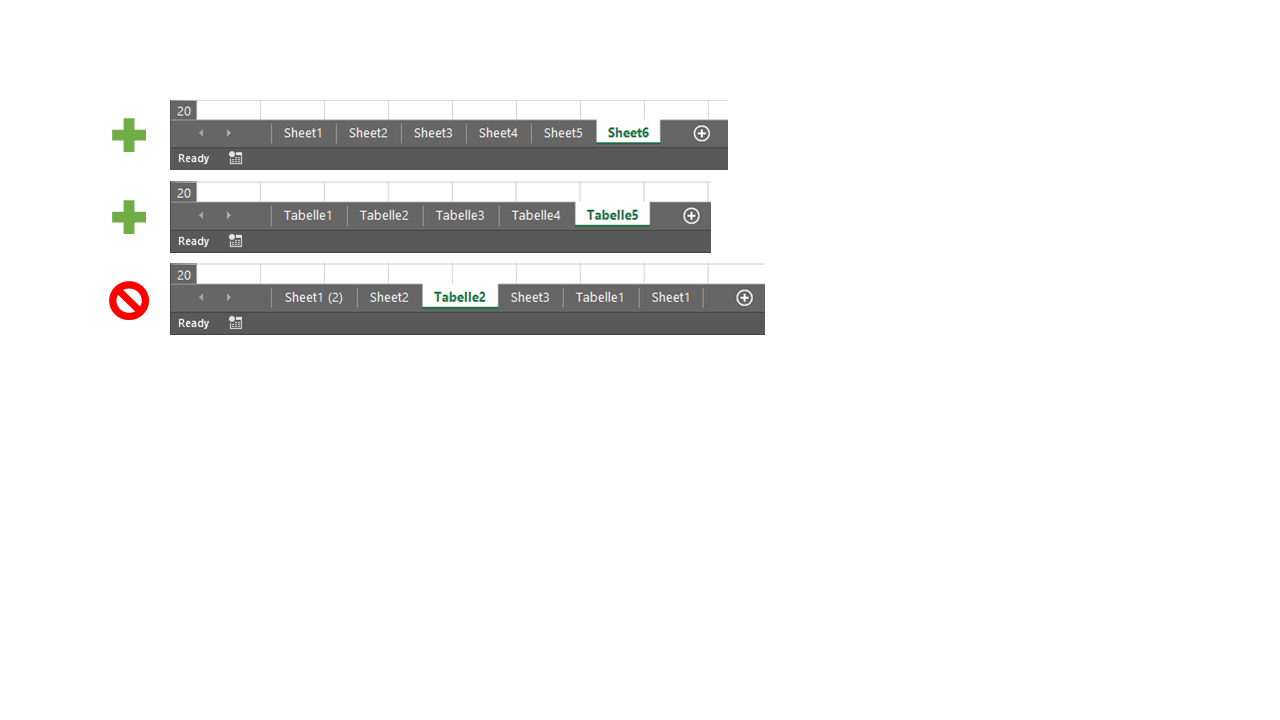


- - Please avoid sheet names with brackets e.g., “Sheet1 (2)”. Rename it as “Sheet2”.


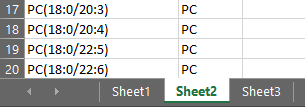


- Please check that all FA residues in the input file are present in the FA list configuration file.

Select the “*In silico* oxidation” generator tab, click on “Open” to upload the PLs table, e.g., “PL_template_for_oxidation.xlsx”.

Once the table is loaded, an “Excel Sheet name” selection list will appear below the path of the input PLs table. Please select the name of the sheet and the PL class accordingly.

To save the .sdf output, click on the save button and choose the folder and file names for the output.


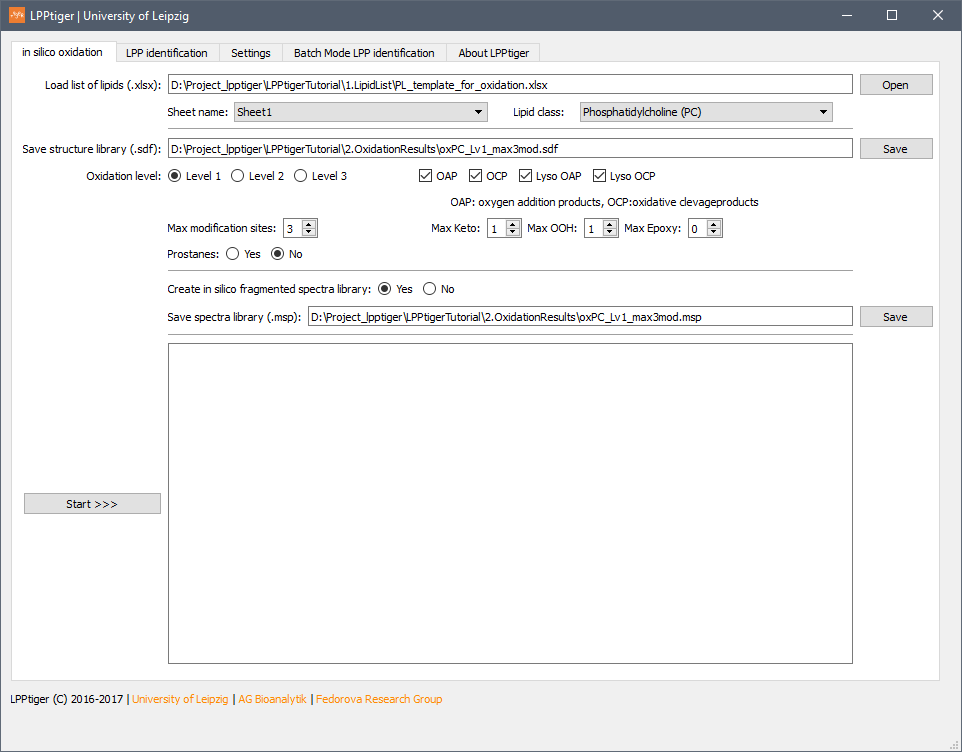


**Parameters for *in silico* oxidation step:**

- **Oxidation level:** Select the level of oxidation according to predefined settings. See [Chapter 7](#_Configuration_files) for more information.
- **Type of the LPPs:** Select types of LPPs you would like to consider for in silico oxidation. Multiple
- **Max number of modification sites:** Limits the number of oxidative modifications (e.g. hydroxy groups) per one FA residue. The default value is set to 3. Can be modify according to the number of *bis*-allylic positions in FAs of choice.
- **Max number of individual modification type:** Limits the number of each modification type. The max number of hydroxyl groups is equal to the maximum number of modification sites.
- **Generation of prostanes and other structures:** Enable or disable the function to generate prostanes and other specific structures.
- **Generation of spectra:** Enable or disable the function to generate predicted spectra library for *in silico* generated LPPs. The prediction of spectra is only available when proper fragmentation pattern is defined. See [Chapter 7](#_Configuration_files) for more information.


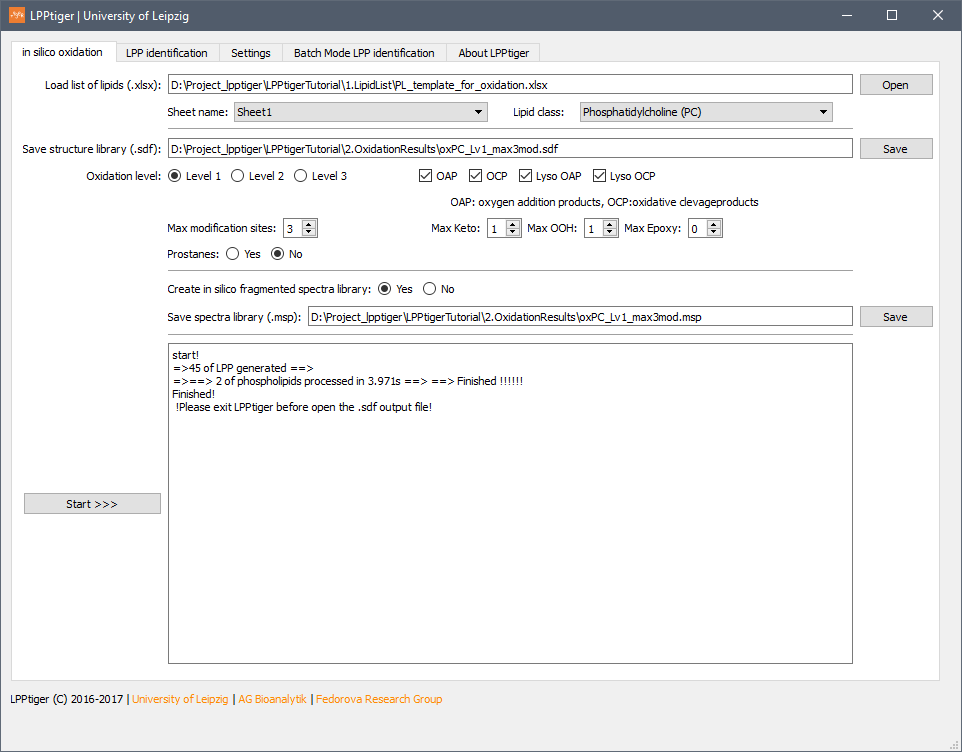


**!!! General Notes**

- The system need some time to finish the generation of the .sdf files in the background. Since the process requires full access of the .sdf file, the operating systems (Windows and Linux) will wait for the exit signal of LPPtiger to finish the generation of .sdf file. Please close LPPtiger and wait few minutes until the system finish the generation of .sdf file.

The program will generate three to four output files, e.g.,

- oxPC_Lv1_ max3mod.sdf
- oxPC_Lv1_max3mod.xlsx
- oxPC_Lv1_max3mod_FA_SUM.xlsx
- (optional) oxPC_Lv1_ max3mod.msp

**.Sdf file** is a structure library of all generated structures with corresponding annotations including 17 predicted properties for each LPP. Developers can use any text editor or any programming language to extract, edit, and process all data entries inside the .sdf file. Please use ChemAxon InstantJ (view, search and modify) or Progenesis SDF studio (view only) to review the contents. SDF files generated by LPPtiger can be imported into other software e.g., Progenesis QI to be used as a structure library for LPP identification.

Two generated **.xlsx files** stores essential parameters for predicted LPPs (oxPC_Lv1_max3mod.xlsx) and all unique FA residues (oxPC_Lv1_max3mod_FA_SUM.xlsx). These two files are used as inputs for the identification steps. Users can edit the file to add and/or remove any entries if necessary.

Optional **.msp file** is generated only when “create *in silico* spectra” is selected. The .msp file contains the predicted spectra generated by *in silico* fragmentation with annotation of each fragment ion. The .msp file is compatible with NIST MS search and MS peptide search programs, and are expected to be compatible with other programs using spectra libraries in .msp format for identification.

**!!! General Notes**

Users can customize two .xlsx files to shorten or enlarge the list of LPPs and corresponding FA lists.

Here are some remarks:

- Please back up these files before editing them.
- For the LPP summary .xlsx tables:


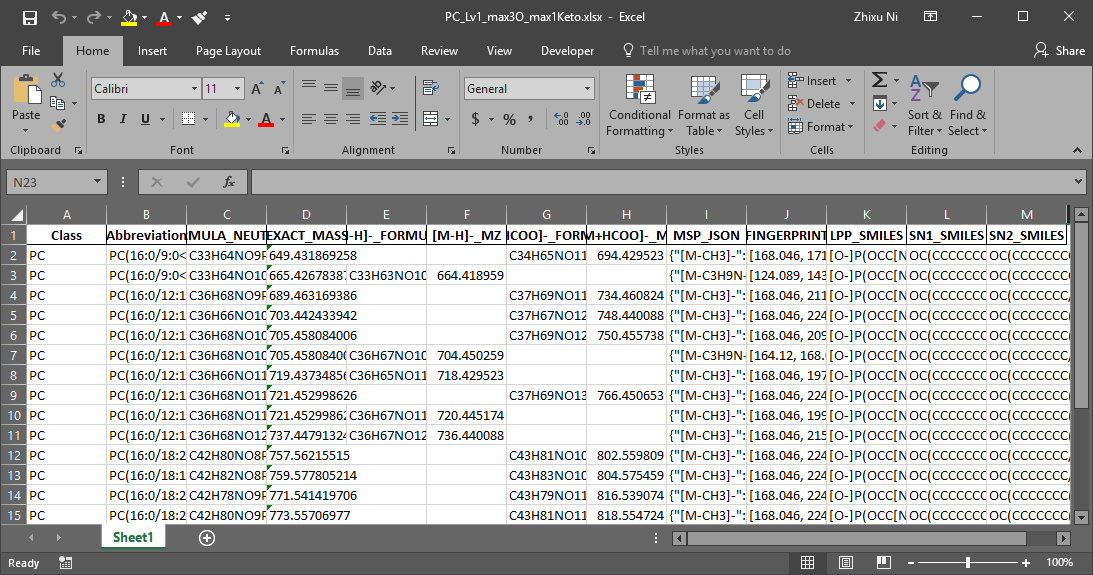


- - The data type and abbreviations are critical for the program, please format the styles of the new entries according to existed fields.
  - The “FINGERPRINT_JSON” and “MSP_JSON” column should be entered properly in the same format as existed entries. Please read more about JSON format (http://www.json.org) and create the code for *m/z* information accordingly.
  - If you are not sure how to generate the information for required data fields, please use a similar structure as a reference.
  - If any error appeared, please use the backup of the original file and try again.
- For the unique FA list:


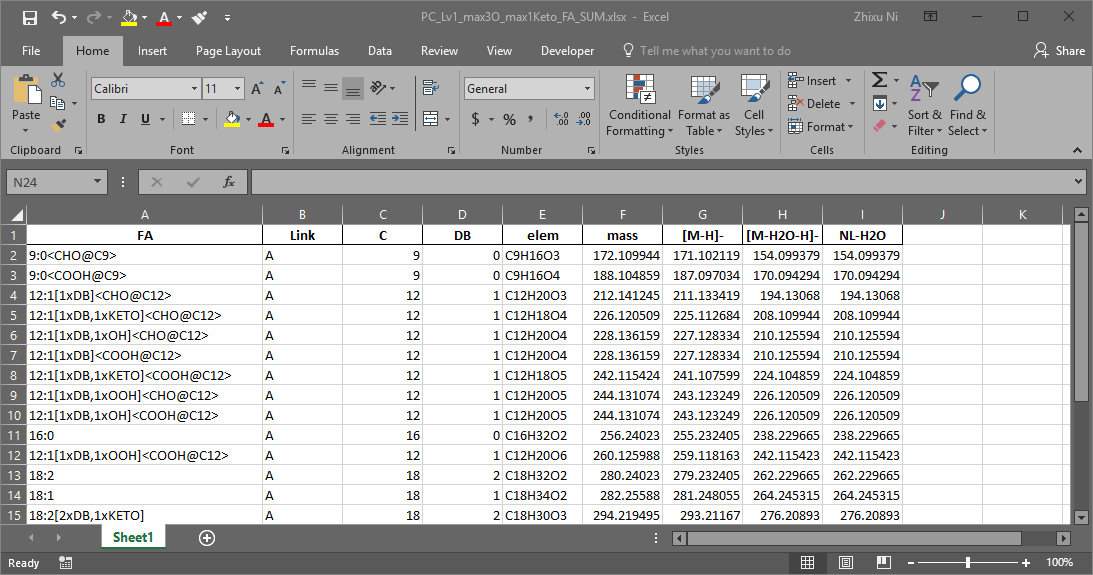


- - Please ensure that all new LPPs manually added to the LPP summary tables have their original FA residues present in this table.
  - The FA list (e.g., oxPC_Lv1_max3mod_FA_SUM.xlsx) should have all the unique FA from the LPP summary table (e.g., oxPC_Lv1_max3mod.xlsx). When it is necessary, the table can be extended to include additional FA e.g., FA 17:0 from the internal standards or common impurities to indicate corresponding signals in the MS/MS spectra.
  - The data type and abbreviations are critical for the program, please format the styles of the new entries according to the existing data.
  - Addition or removal of any entry in the FA list (e.g., FA 17:0) may influence the specificity score due to the possible changes of total intensities of the unspecific peaks. Due to additional structure unspecific signals identified using the modified FA list (e.g., FA 17:0), the Specific score may decrease and the overall LPPtiger score can be lower than before. Thus, additional FA in the list may reduce possible false positives and improve identification accuracy.
  - If any error appeared, please use the backup original file and try again.

## Step II: Hunt for LPPs

Defined parameters are extremely important for the identification accuracy. Please use the following parameters for the test file, and find optimized values for your dataset.


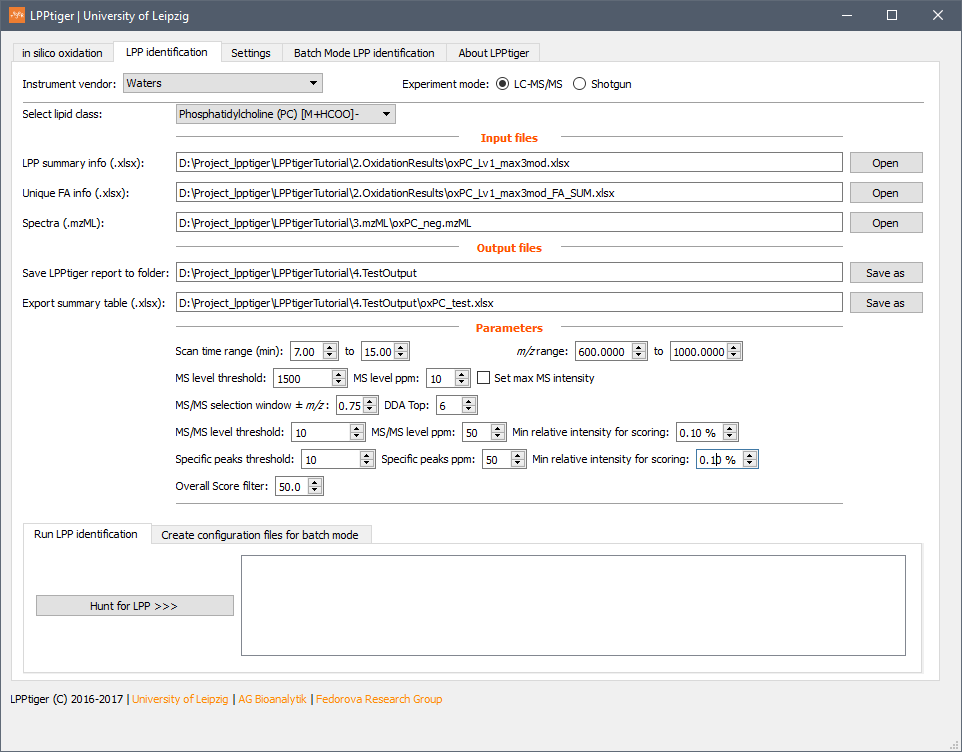


**Input files and mode selection:**

- **Instrument vendor:** Currently, LPPtiger supports .mzML files generated from .raw/.RAW files from **Waters** and **Thermo Fisher Scientific** instruments; working with other files might require adjustment of the file format.
- **Experiment Mode:** select between LC-MS/MS or shotgun.
- **LPP summary info** **(.xlsx):** The summary table of all LPPs generated during *in silico* oxidation step (e.g. oxPC_Lv1_max3mod.xlsx).
- **Unique FA info** **(.xlsx):** The summary table of unique FA residues generated during *in silico* oxidation step (e.g. oxPC_Lv1_max3mod_FA_SUM.xlsx).
- **Spectra (.mzML):** Spectra file in .mzML format to be searched for identification. Please make sure that the .mzML file selected matched to the vendor and experiment mode.
- **Save LPPtiger report to folder:** Choose the path for the HTML report file and corresponding image folder.
- **Export summary table (.xlsx):** Choose the path for the summary output table.

**Parameters:**

- **Ranges:** Scan time (retention time) and *m/z* ranges
- **Threshold:** MS level threshold, MS/MS level threshold, and specific peaks threshold. (optional: a max intensity filter can be defined to focus on LPPs within a certain intensity range)
- **ppm:** Mass accuracy limits defined for MS level, MS/MS level, and structure specific signals.
- **Min relative intensity for scoring:** Defined for MS/MS level and structure specific signals.
- **Overall score filter:** Only LPPs with a score higher than defined will be reported.
- **Other parameters:** DDA top number and MS/MS precursor selection window

Click “***Hunt for LPP***” to start!

LPPtiger creates a log file named “LPPtiger_Params-Log_YYYY-MM-DD_HH-MM-SS.txt” and an HTML file with associated folder of image and “LPPtiger_Results_YYYY-MM-DD_HH-MM-SS.html”.

You can open the HTML report in your web browser and refresh from time to time to check the latest results. It may take few minutes until the first image is generated. We recommend to use Mozilla Firefox, Chrome or Chromium to review the HTML report.

**!!! General Notes**

LPPtiger will not respond during the run. The process may take some time (e.g., up to 3 hours for a 50 min LC-MS dataset from Waters Instruments), please be patient.


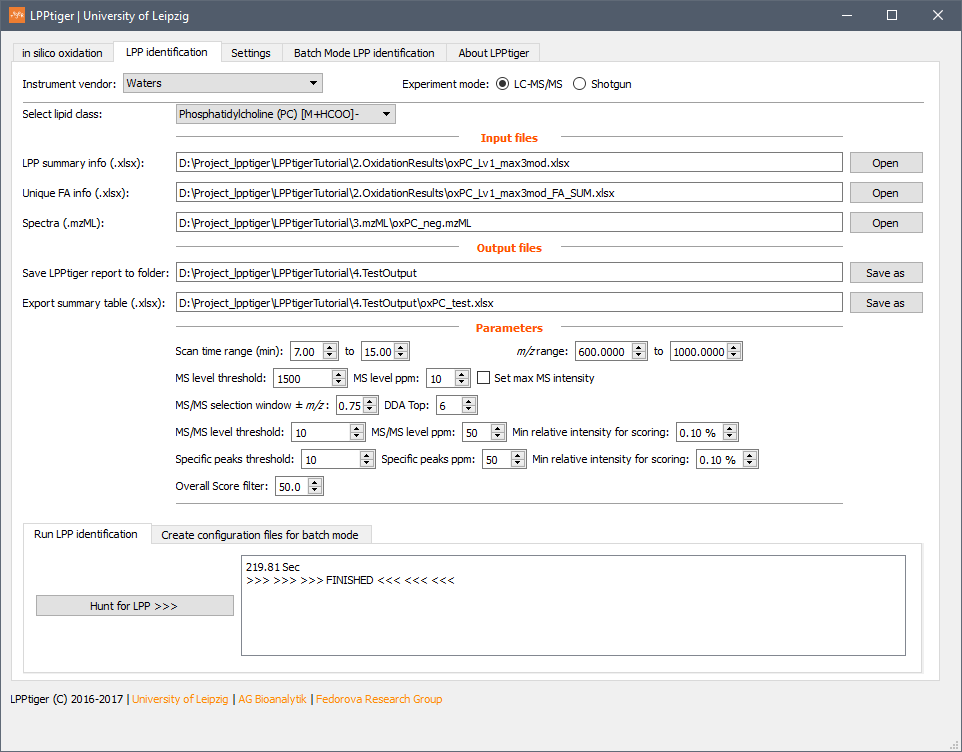


## Step III batch mode

Instead of running individual files separately with manual input of all parameters, users can save all parameters to a configuration text file which can be submitted to the integrated batch mode. The configuration file can be generated from “**Create configuration files for batch mode**” tab next to the “**Run LPP identification**” tab.


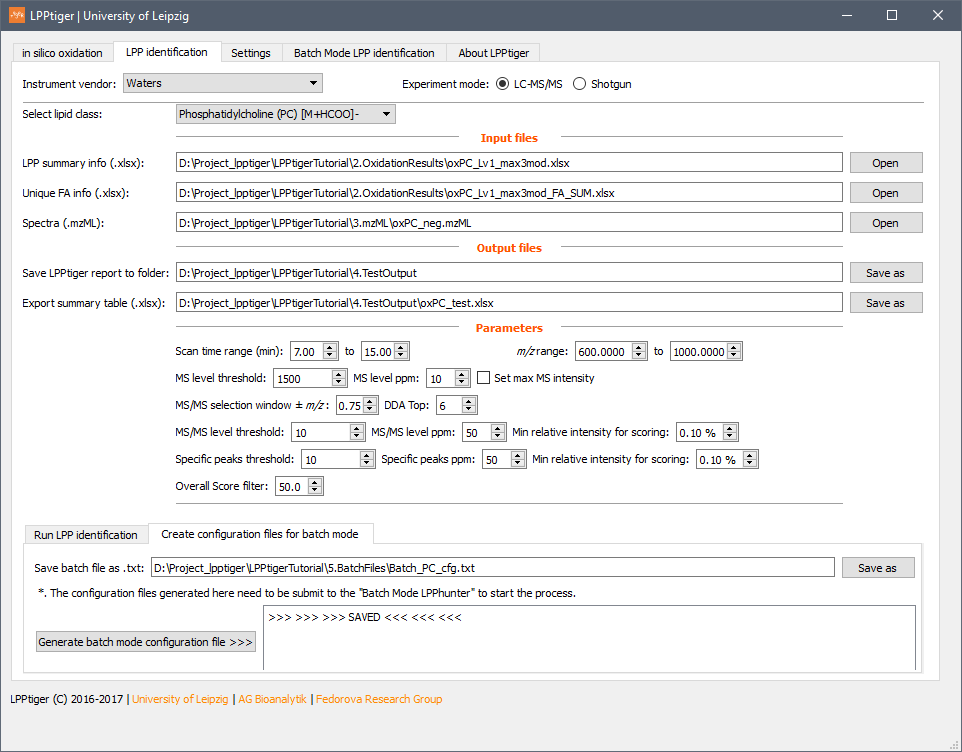


Saved configuration files can be edited by any text editor.

After generation of all configuration files, please click on the “**Batch Mode LPP identification**” tab, add individual files by “**Add single file**” or load all .txt configuration files by “**select a folder**”, then click on “**Run Batch Mode**” to run.

The default mode is set to process all configurations files one by one as a sequence.


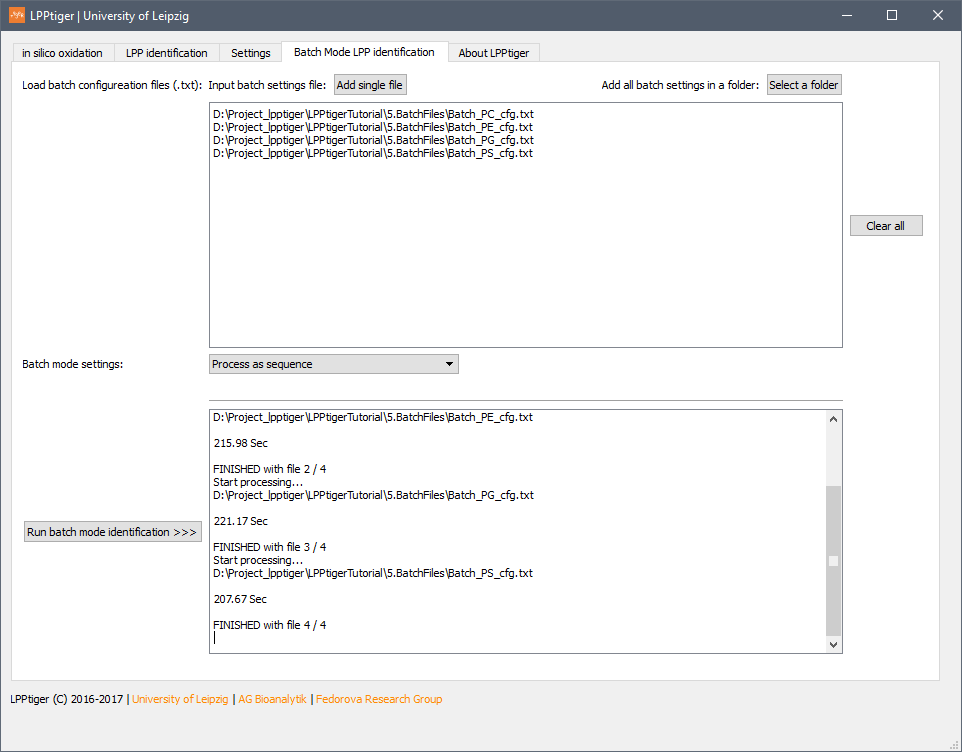


However, for high configuration servers, optional mode to process several files in parallel is supported.


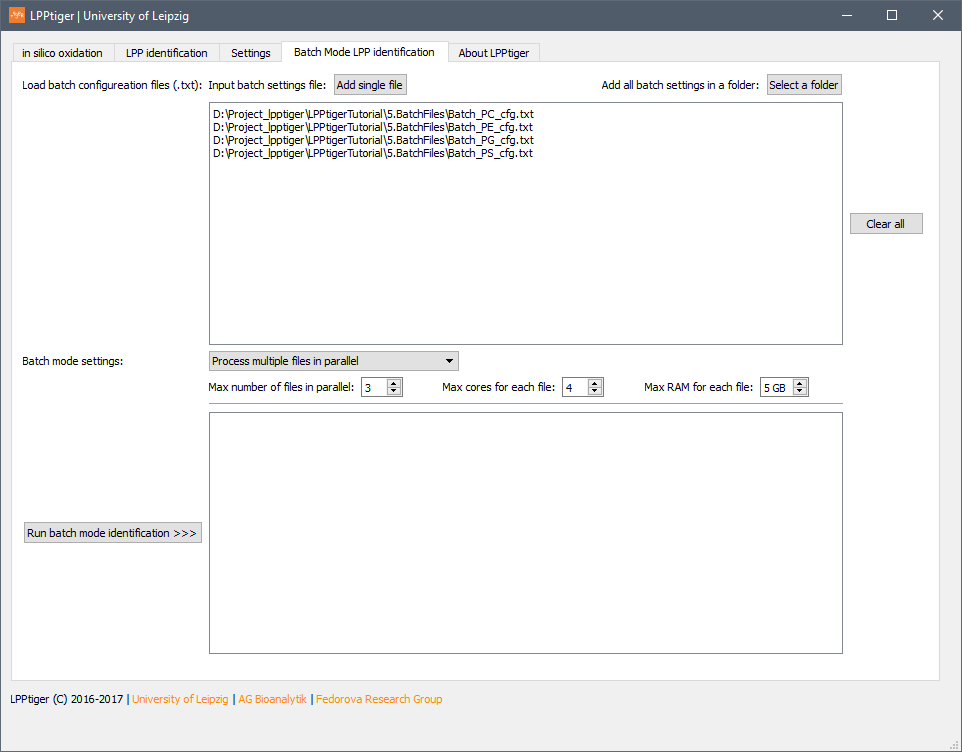


**!!! General Notes**

- Max core number and RAM size parameters for the batch mode parallel processing have to be defined here, while the initial values in the configuration files will not be used.

# LPPtiger output

For each submitted dataset LPPtiger provides the **output .xlsx table** which summarize lipid identities (bulk identification, proposed discrete structure, elemental composition, theoretical and observed *m/z* values, mass accuracy, retention time), identification metrics (LPPtiger and isotope scores, relative intensities of matched fragments, PL specific and unspecific signals) and data specific details (DDA rank, scan number).

LPPtiger generates a separate **six-panel image for each identified LPP**:


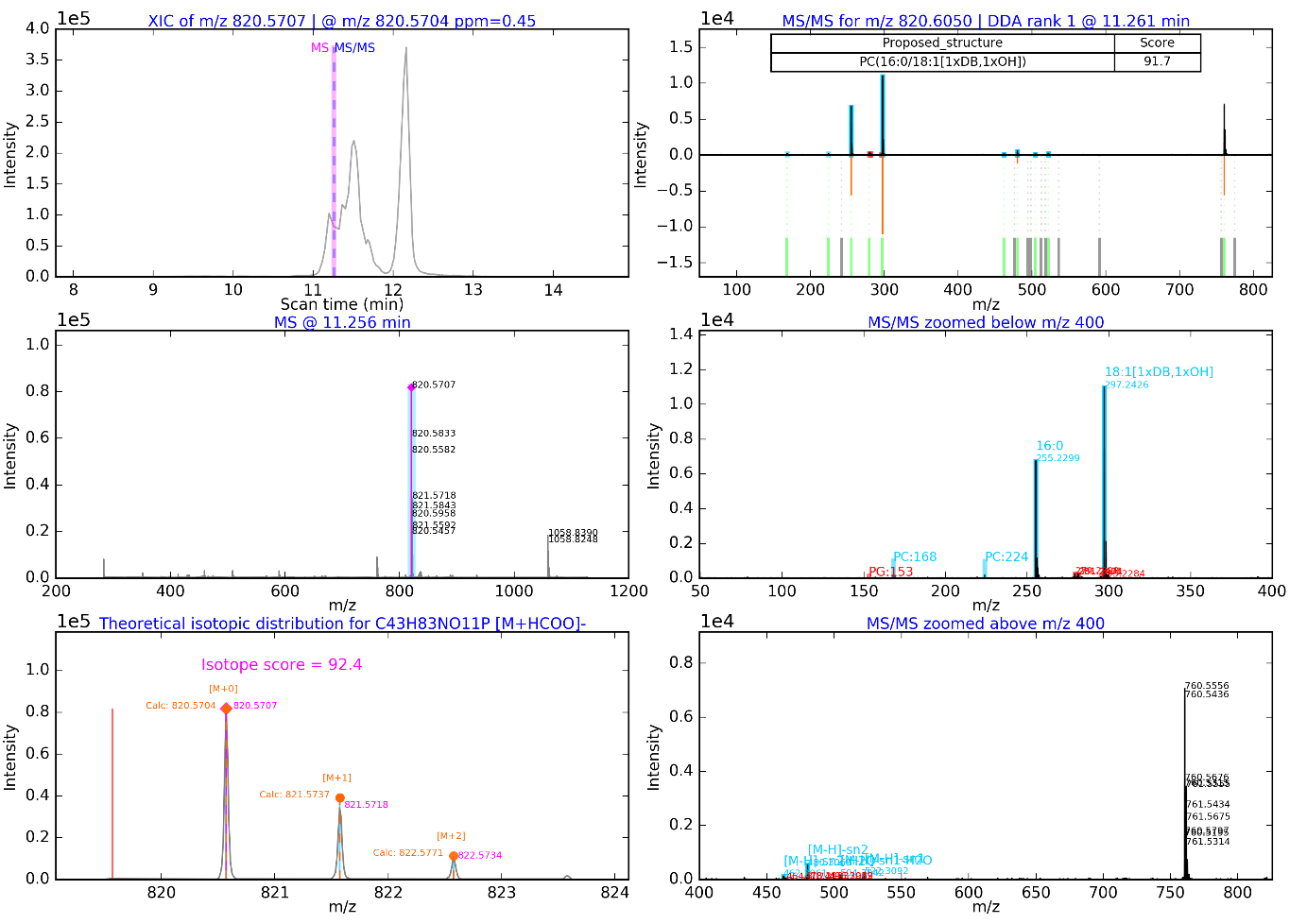


A

B

C

D

E

F

Example of the six-panel image from LPPtiger .html report for precursor at *m/z* 846.5789 identified as PC(18:0/18:1[1xDB,1xKETO]).

1. extracted ion chromatogram (XIC)
2. corresponding MS scan
3. zoomed MS scan with isotope pattern and Isotope Score
4. MS/MS with identification information
5. MS/MS zoomed at the region of fatty acid fragments
6. MS/MS zoomed at the region of neutral loss signals

**!!! General Notes**

- PL class specific (marked also in cyan color with PL name) and unspecific (red) fragment ions in panel D, E and F are considered for the specificity score only and not used for the rank score in LPPtiger.

All output images are integrated and indexed in an informative **HTML report file** for manual reviewing. Availability of the graphical data representation and organized report files allows fast evaluation of identification results. HTML report file with a built-in identification table and a log of the corresponding LPPtiger parameters provides a simple solution for data tracking and storage.


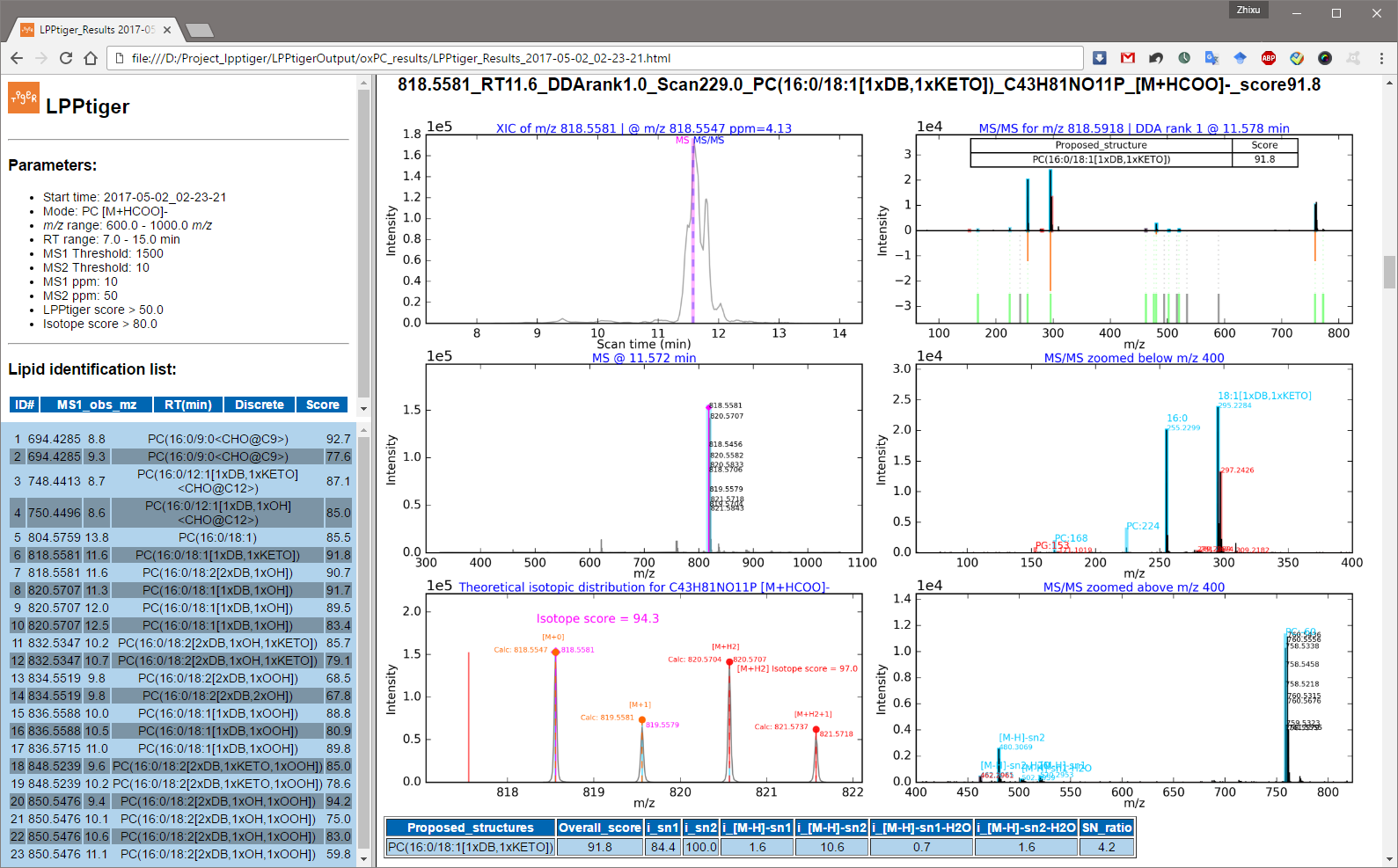


A

B

C

D

Screenshot of LPPtiger HTML output .html report file opened in Mozilla Firefox browser. The LPPtiger report file provides a simple and organized solution to review, store and exchange the assigned outputs. The rich information .html report file from LPPtiger has four major parts: (A) the main parameters used for the identification, (B) the overall identification table, (C) the output images and (D) a relative intensity table for each identified lipid. Users can navigate between output images by clicking on the corresponding entry in the identification table. The original image can be accessed by clicking on the six-panel image (B).


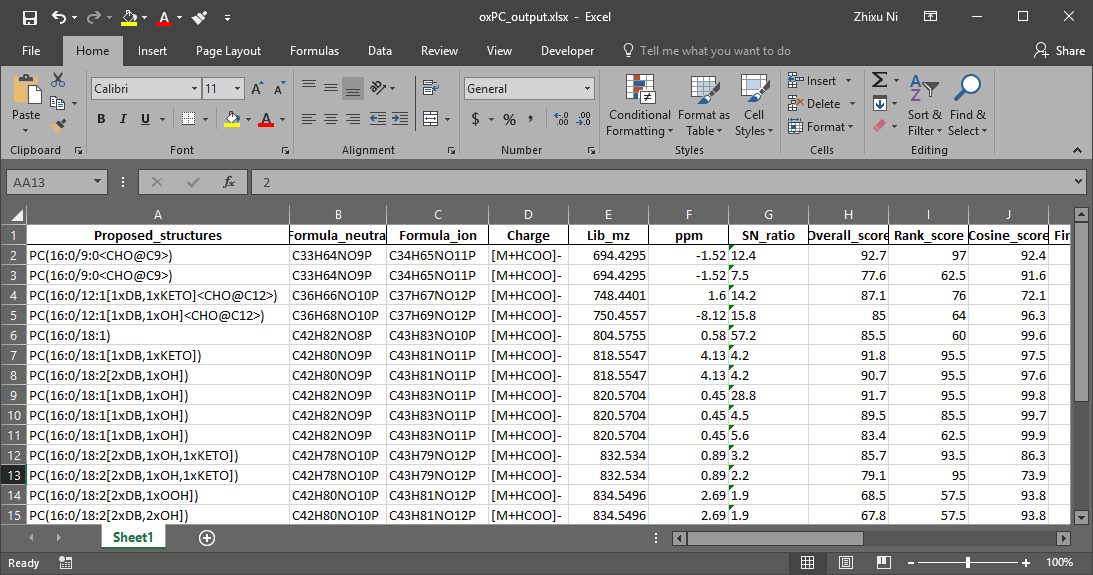


All the information in the html report file are also available in an excel file which contains the summary from all the identifications. The individual scores of all identifications are exported to this excel and named accordingly.

# Configuration files

You can easily modify/create configuration files to define your own FA preferences using template files provided with LPPtiger. You can also adapt PL specific fragments list and weight factors depending on the MS instruments, collision energies and ion adducts used in the study by modifying simple .xlsx tables provided with LPPtiger:

1. .csv configuration file of modification types and levels for single double bond unit
2. .csv configuration file of fatty acids for oxidation
3. .csv configuration file of prostanes and other special LPP types for three or more double bond unit
4. .csv configuration file of abbreviation settings for prostanes and other special LPPs
5. .xlsx configuration file of phospholipid fragmentation patterns
6. .xlsx configuration file of phospholipid head group specific fragments and neutral losses
7. .xlsx configuration file for the fragmentation rank score settings.

**!!! Note:**

All default configuration files are located under “LPPtigerFolder\ConfigureationFiles”. You can save modified configuration files in different folders and change them accordingly for different data.


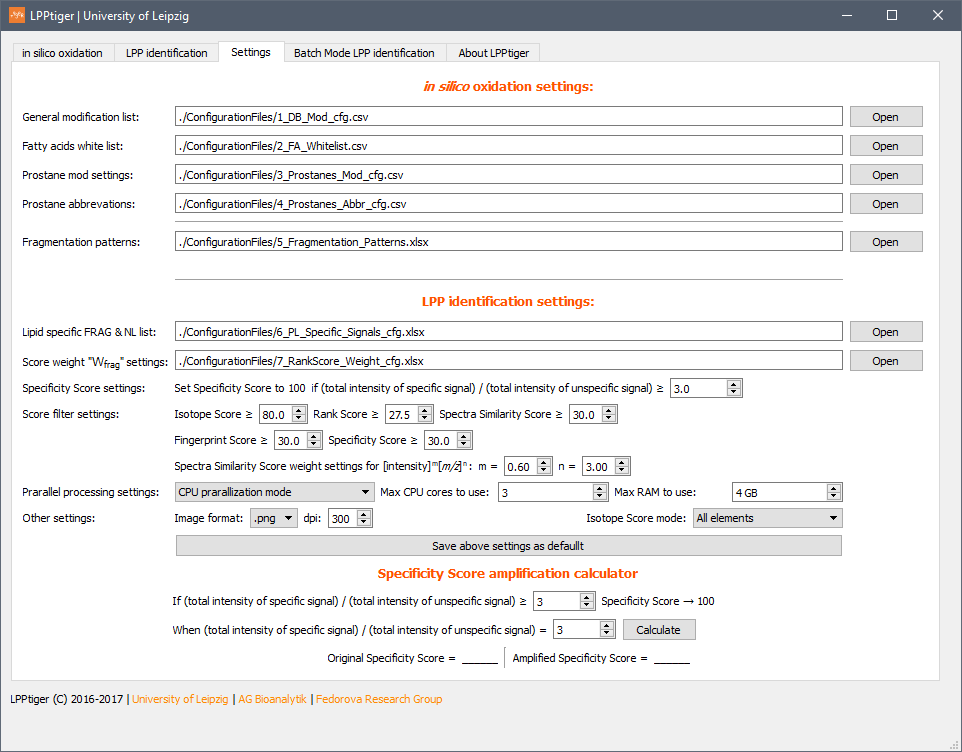


**!!! Note:** If you edit any .csv configuration file in Excel, take care that FA abbreviation e.g. 14:0 are saved correctly and not converted to the time format (e.g. 14:00:00). Check modified .csv file by opening it with Notepad.

1. Screenshot of .csv configuration file of modification types and levels for a single double bond unit


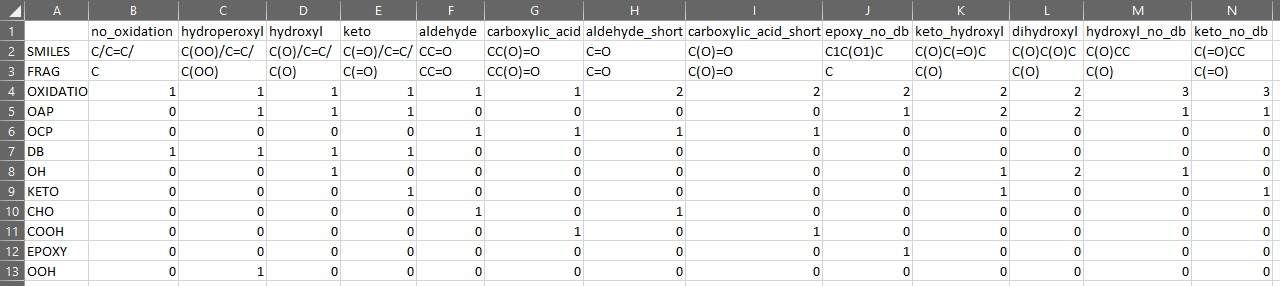


The three levels of oxidation are defined by as follows:


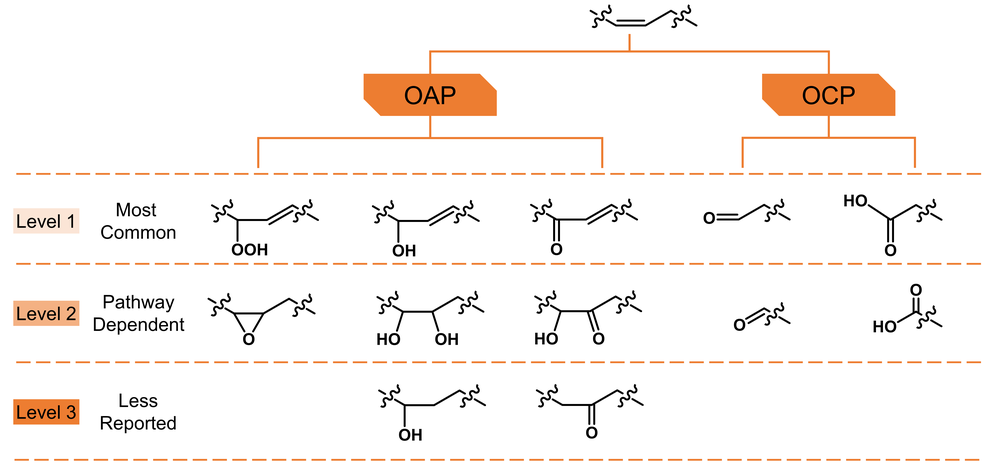


1. .csv configuration file of fatty acids for oxidation


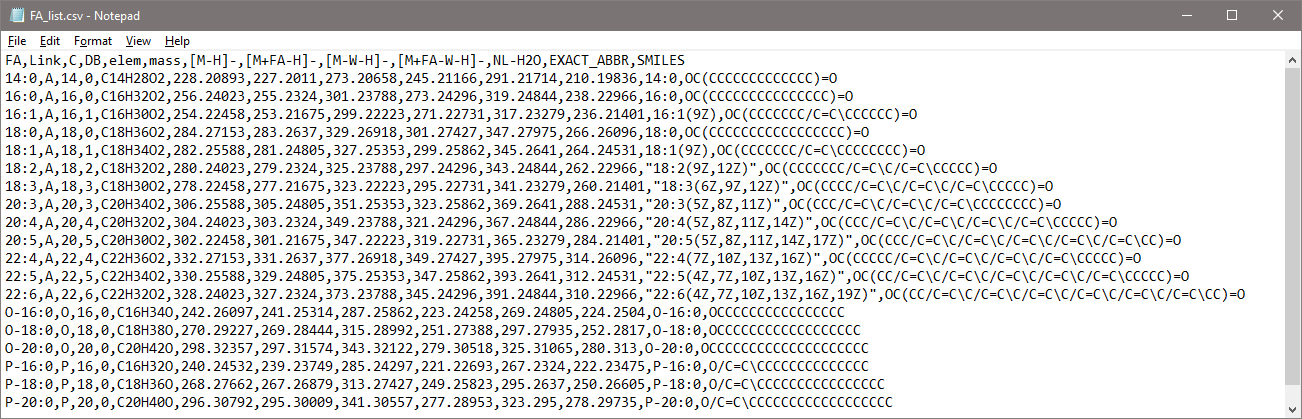


1. .csv configuration file of prostanes and other special LPP types for three or more double bound unit


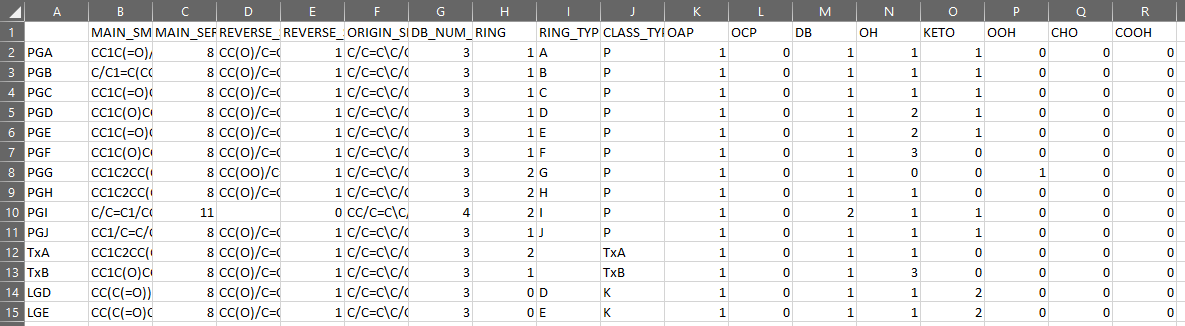


1. .csv configuration file of abbreviation settings for prostanes and other special LPPs


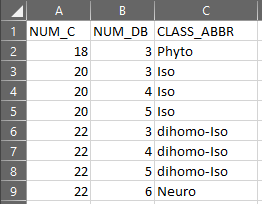


1. .xlsx configuration file of phospholipid fragmentation patterns


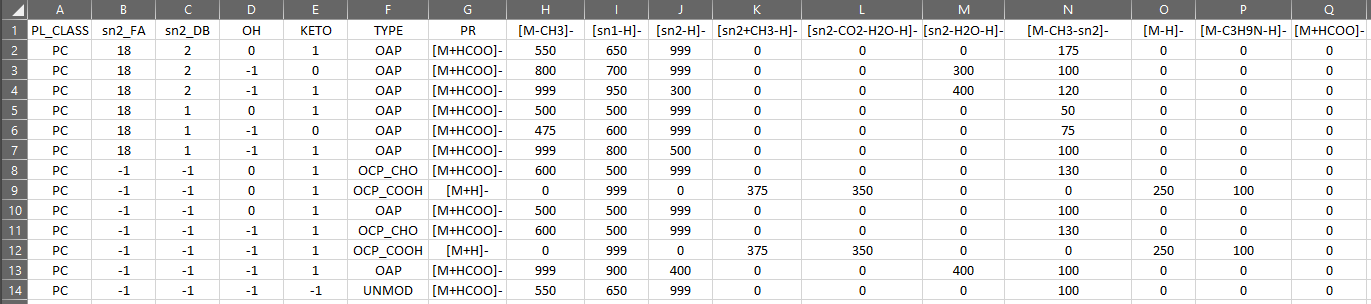


1. .xlsx configuration file of phospholipid head group specific fragments and neutral losses


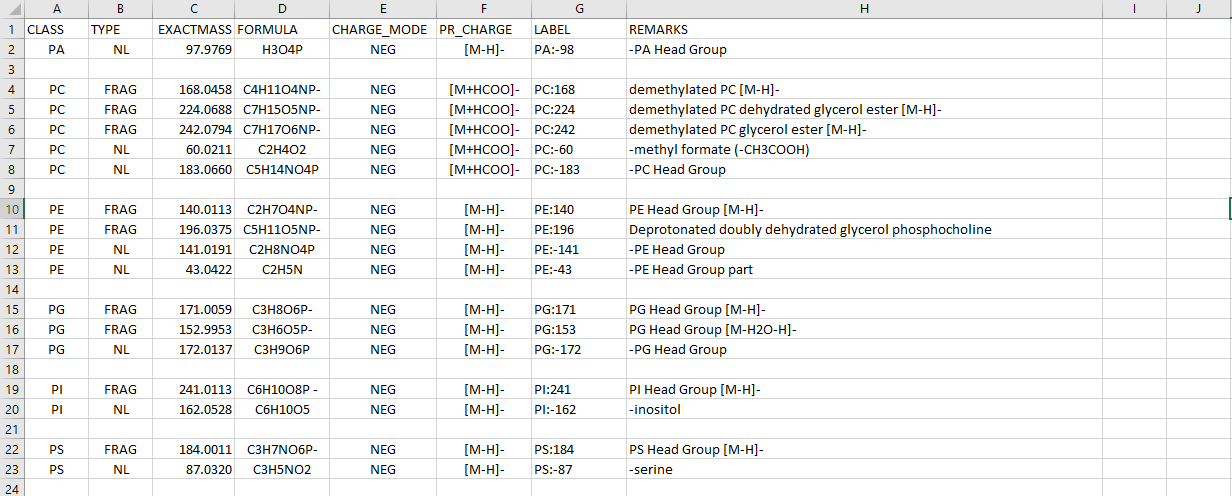


1. .xlsx configuration file for the fragmentation rank score settings


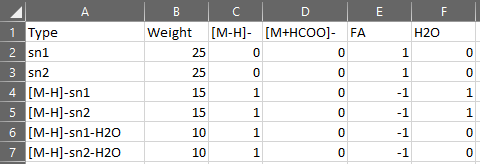

Supplement: Supplementary file 19 — Supplementary File 11 [file 41598_2017_15363_MOESM19_ESM.doc]
