# Supplementary material for: LPPtiger software for lipidome-specific prediction and identification of oxidized phospholipids from LC-MS datasets
Source: Sci Rep. 2017 Nov 9;7:15138. doi: 10.1038/s41598-017-15363-z (PMC5680299; doi:10.1038/s41598-017-15363-z)

1. LPPtiger identification examples from training datasets (*in vitro* oxidized PLs)

**Example 1:** LPPtiger identification report and corresponding original CID spectrum for ion at *m/z* 694.43^-^ (RT 8.8 min) identified as PC(16:0/9:0<CHO@C9>) or 1-palmitoyl-2-(9-oxononanoyl)-*sn*-glycero-3-phosphocholine in *in vitro* oxidized PC samples.


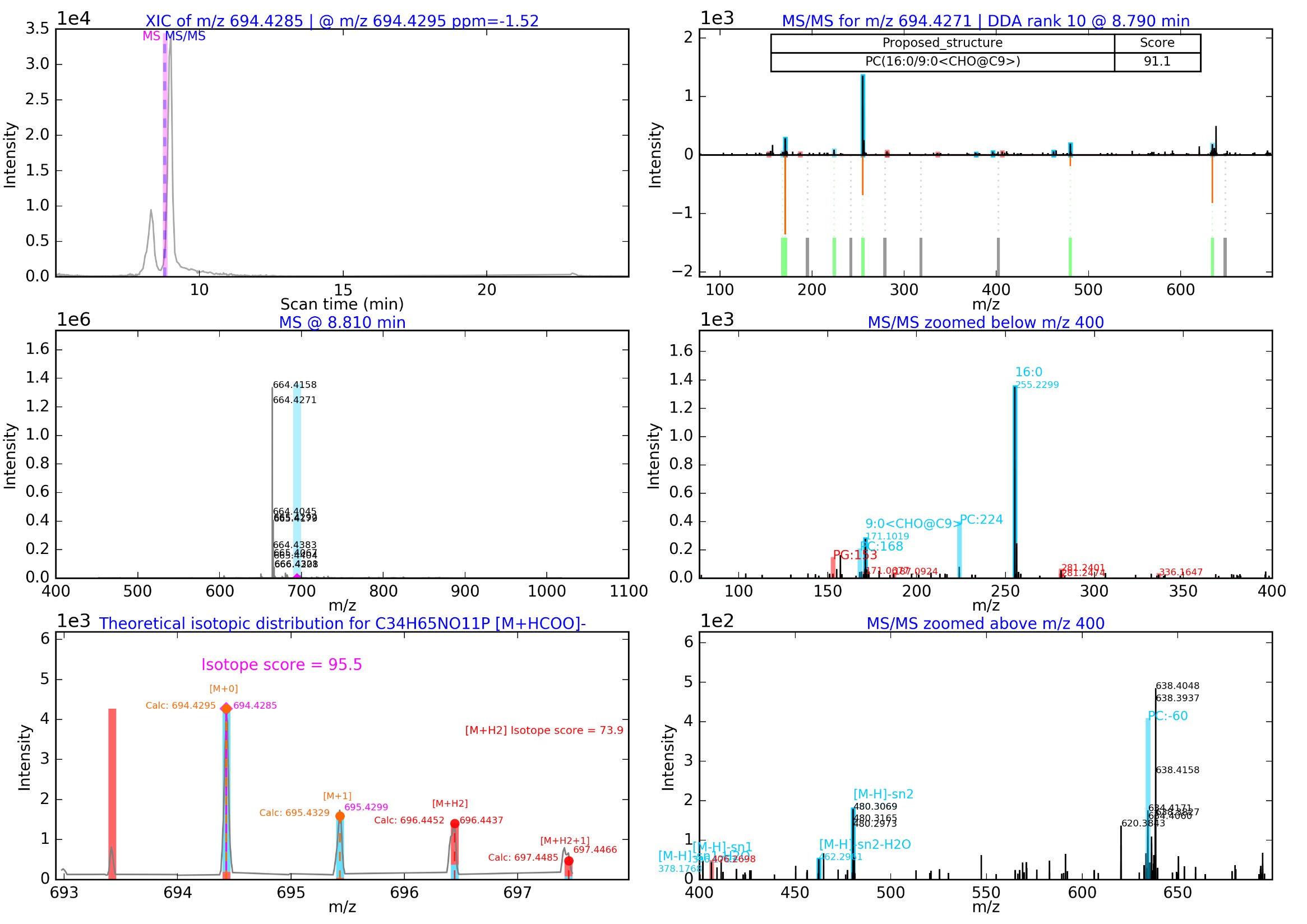


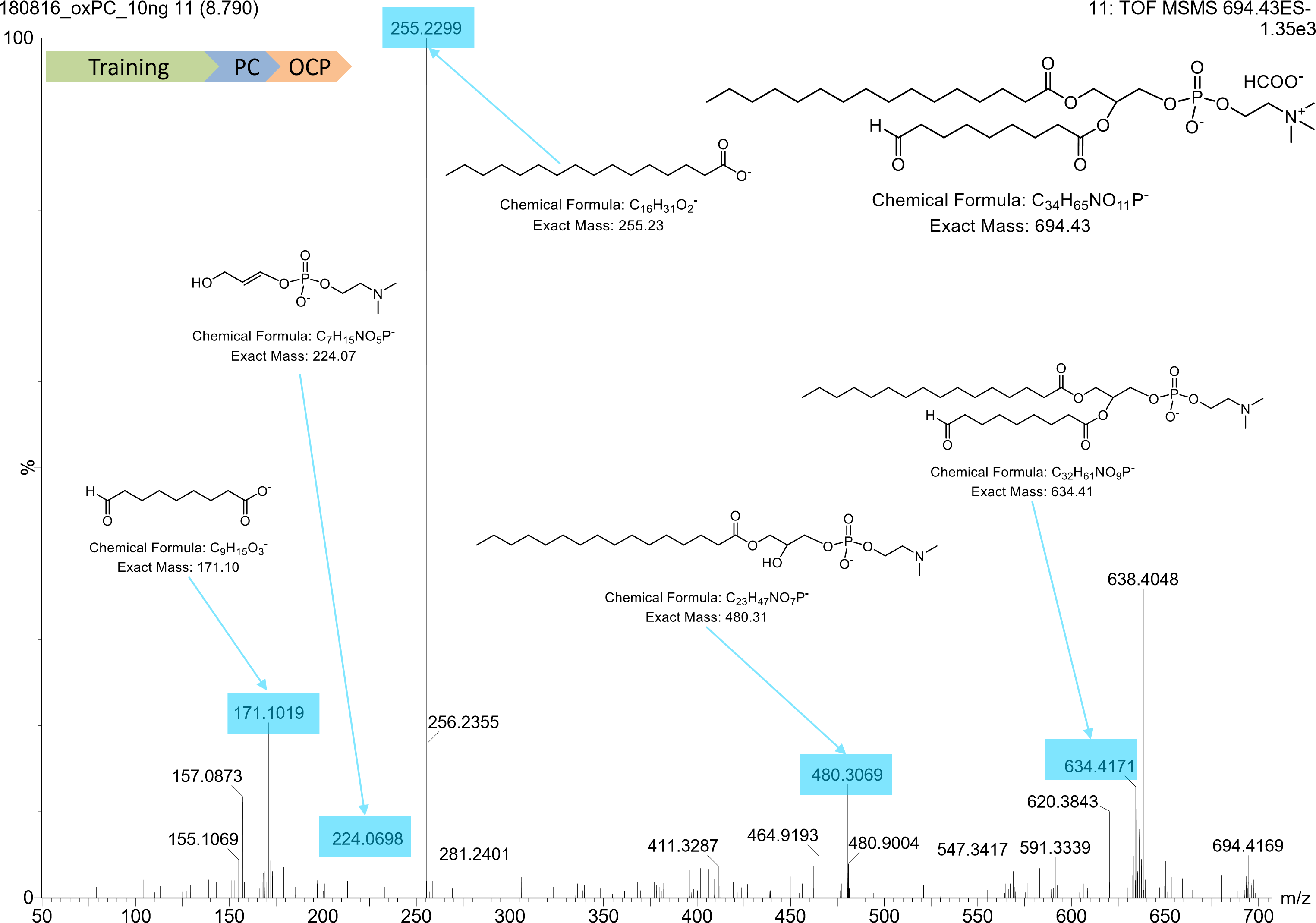


**Example 2:** LPPtiger identification report and corresponding original CID spectrum for ion at *m/z* 818.59^-^ (RT 11.6 min) identified as PC(16:0/18:2[2xDB,1xOH]) or 1-palmitoyl-2(hydroxy-octadecadienoyl)-sn-glycero-3-phosphocholine in *in vitro* oxidized PC samples.


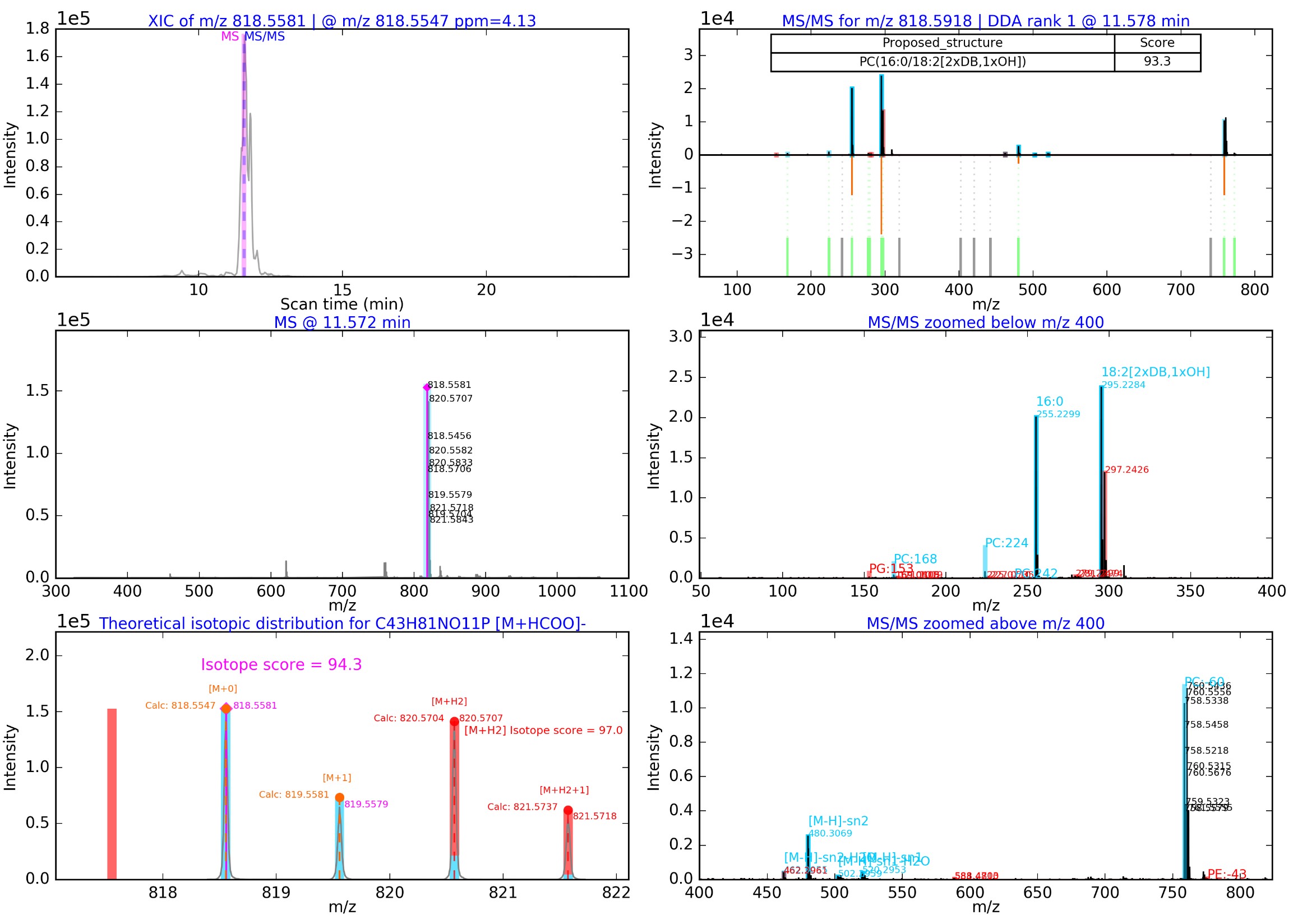


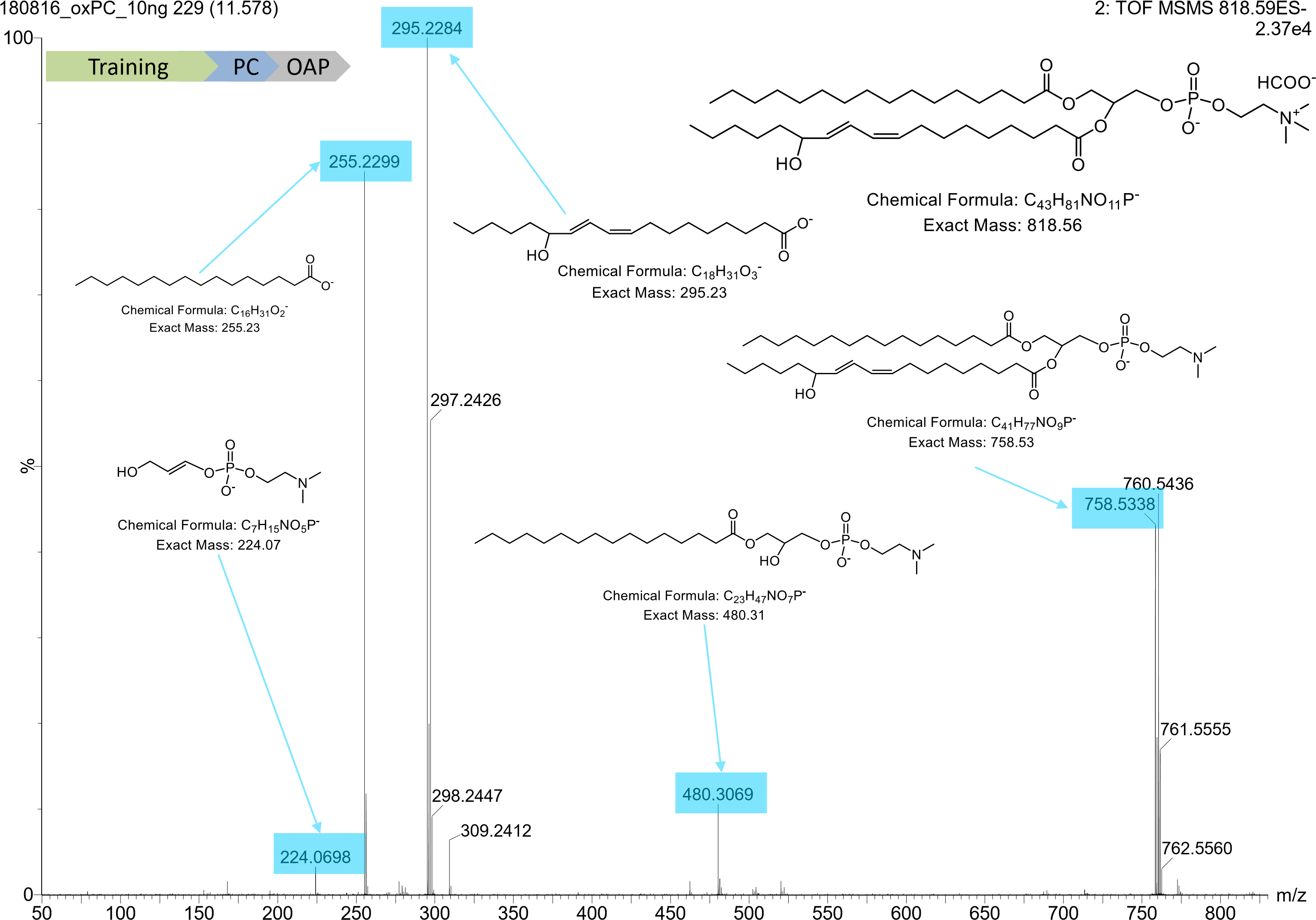


**Example 3:** LPPtiger identification report and corresponding original CID spectrum for ion at *m/z* 536.25^-^ (RT 8.6 min) identified as PE(16:0/4:0<CHO@C4>) or 1-palmitoyl-2-(4-oxobutanoyl)-sn-glycero-3-phosphoethanolamine in *in vitro* oxidized PE samples.


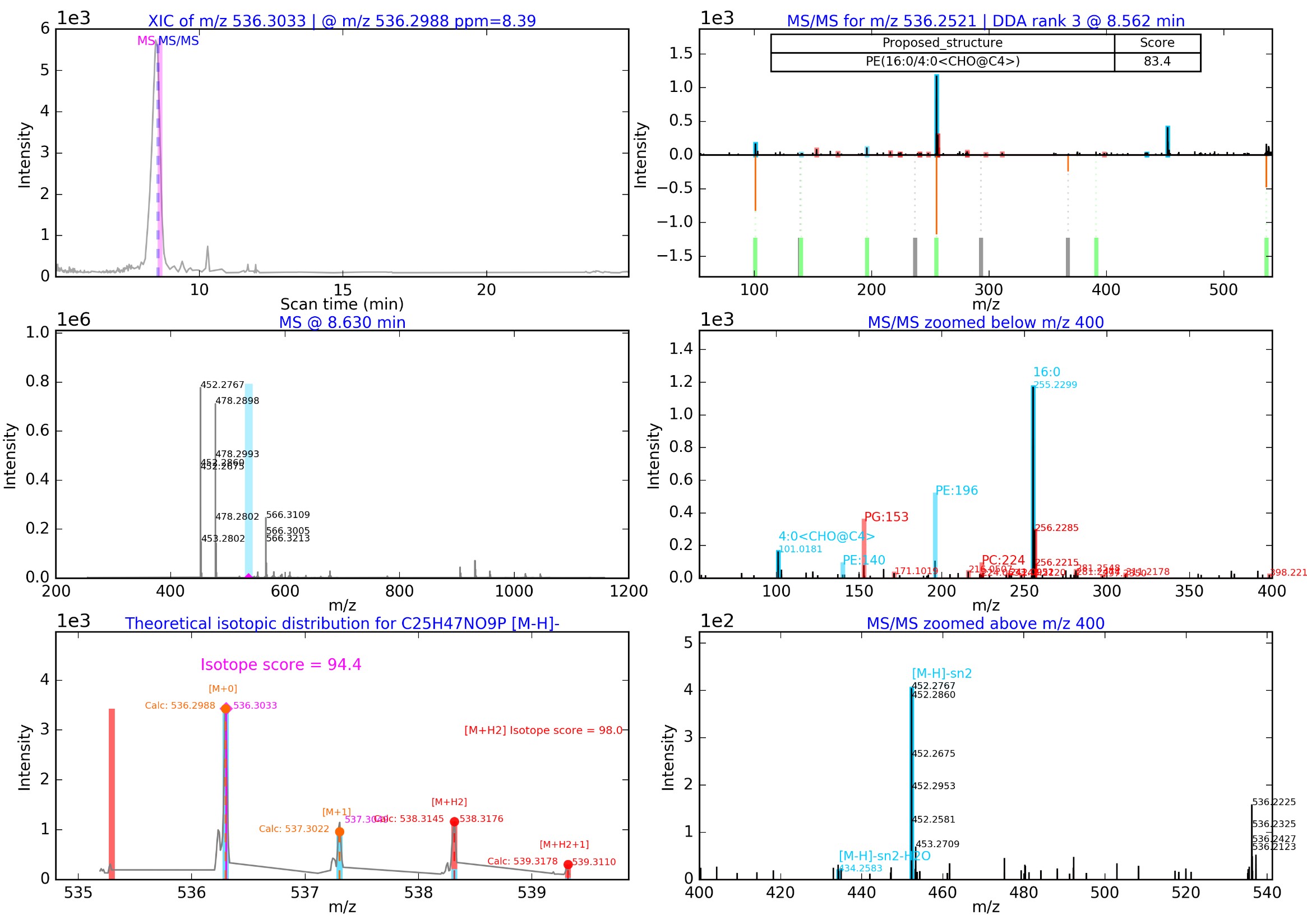


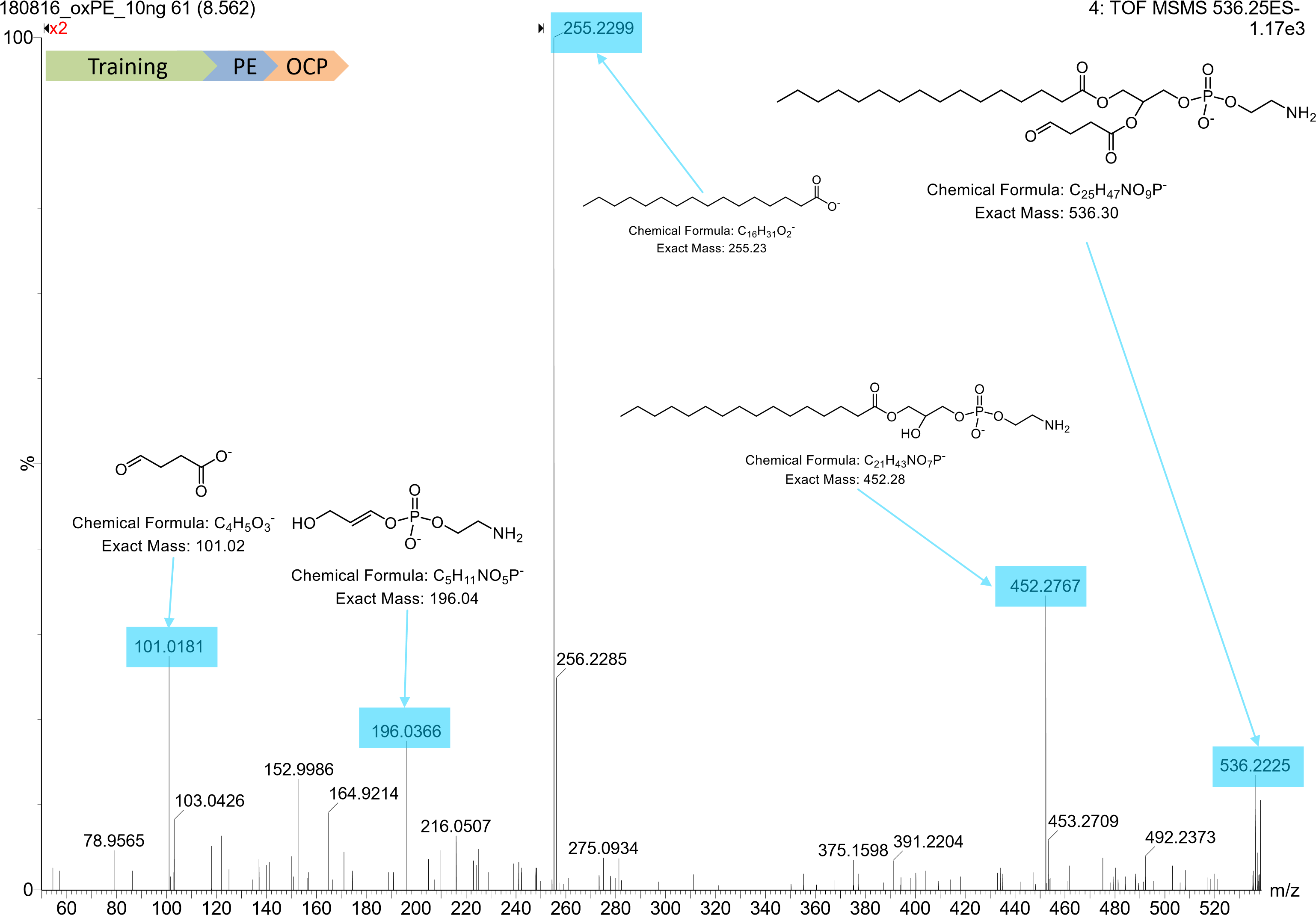


**Example 4:** LPPtiger identification report and corresponding original CID spectrum for ion at *m/z* 606.36^-^ (RT 9.1 min) identified as PE(16:0/9:0<CHO@C9>) or 1-palmitoyl-2-(9-oxononanoyl)-sn-glycero-3-phosphoethanolamine in *in vitro* oxidized PE samples.


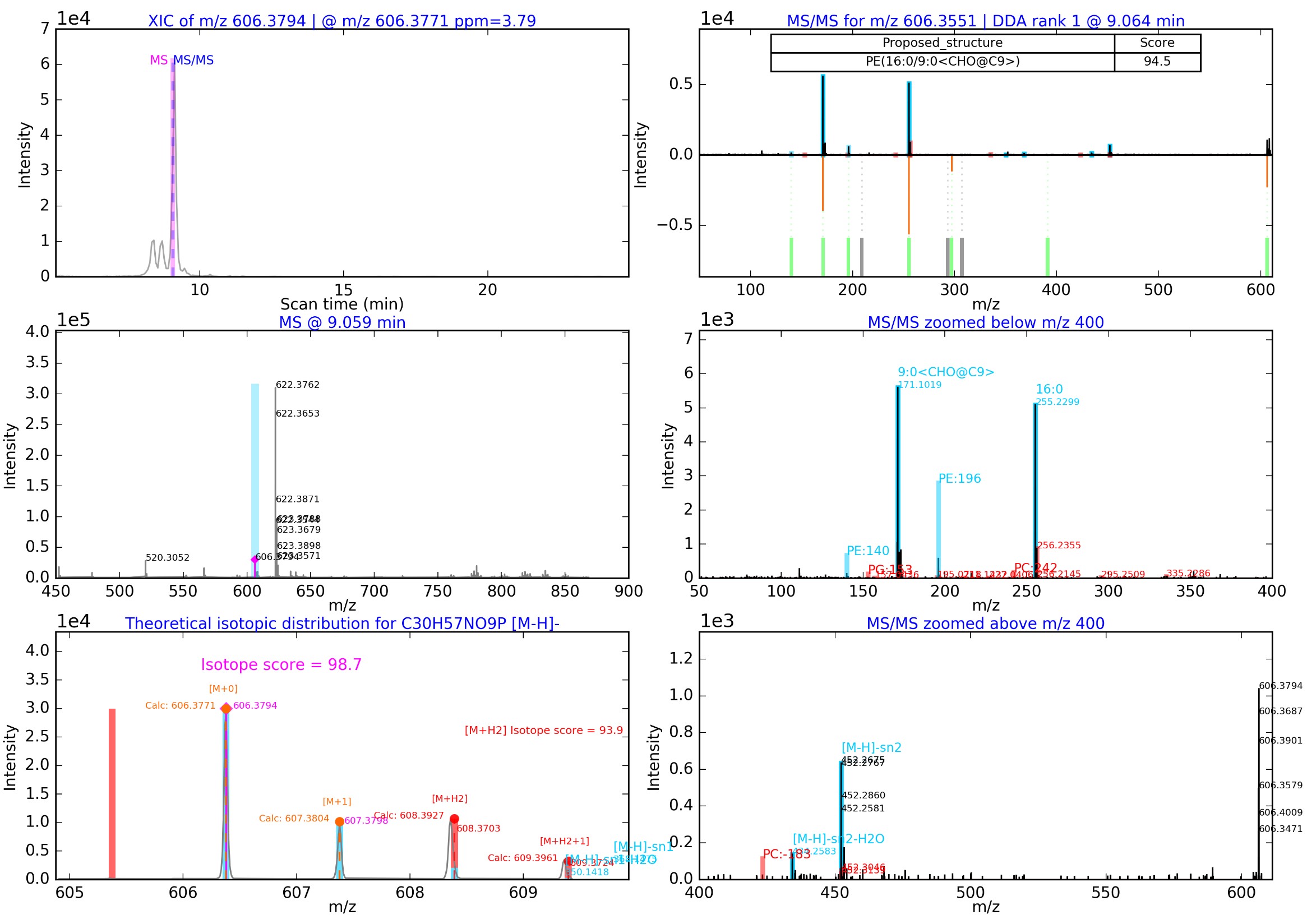


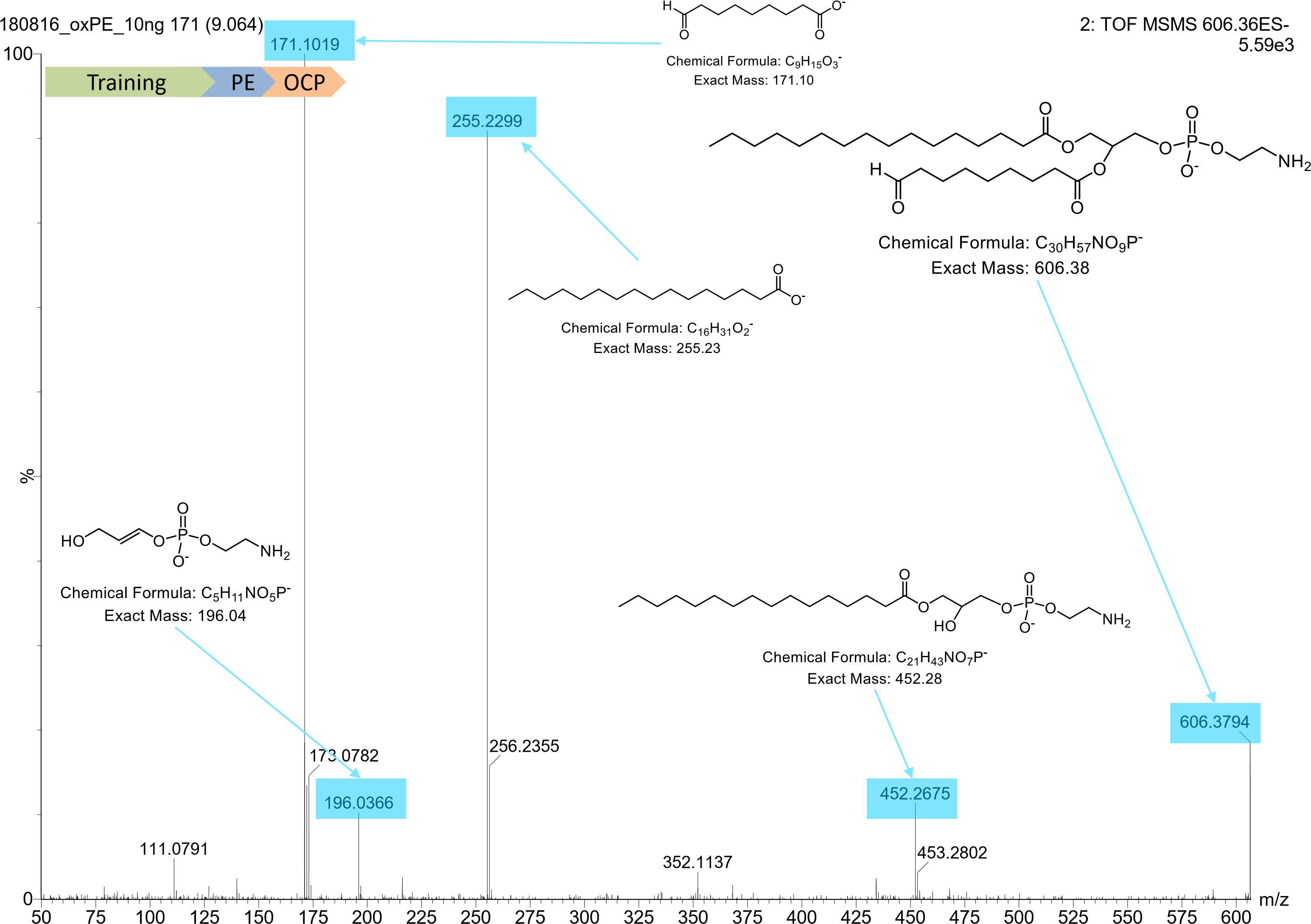


**Example 5:** LPPtiger identification report and corresponding original CID spectrum for ion at *m/z* 660.38^-^ (RT 8.8 min) identified as PE(16:0/12:1[1xDB,1xKETO]<CHO@C12>) or 1palmitoyl-2-(dioxo-dodecenoyl)-sn-glycero-3-phosphoethanolamine in *in vitro* oxidized PE samples.


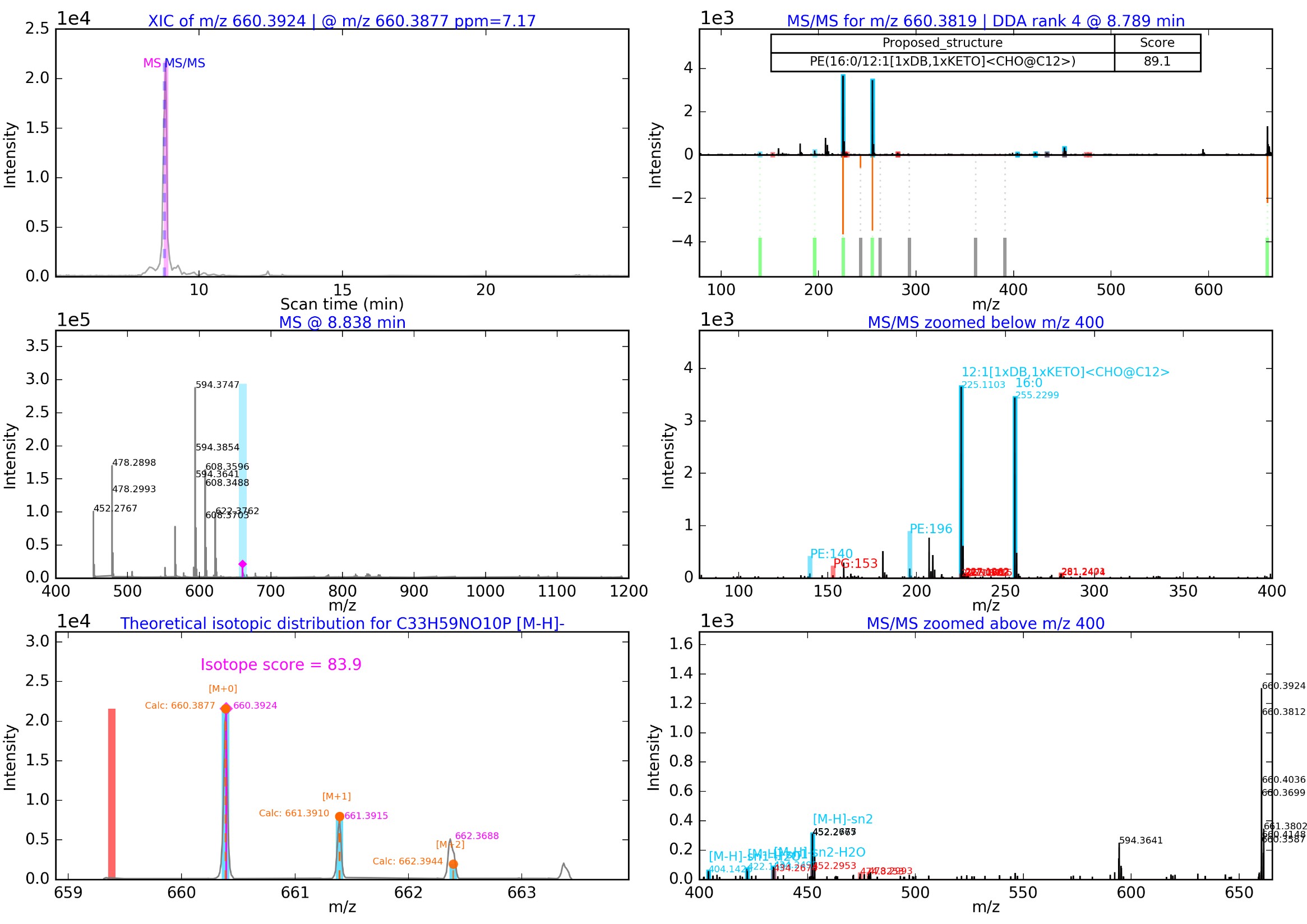


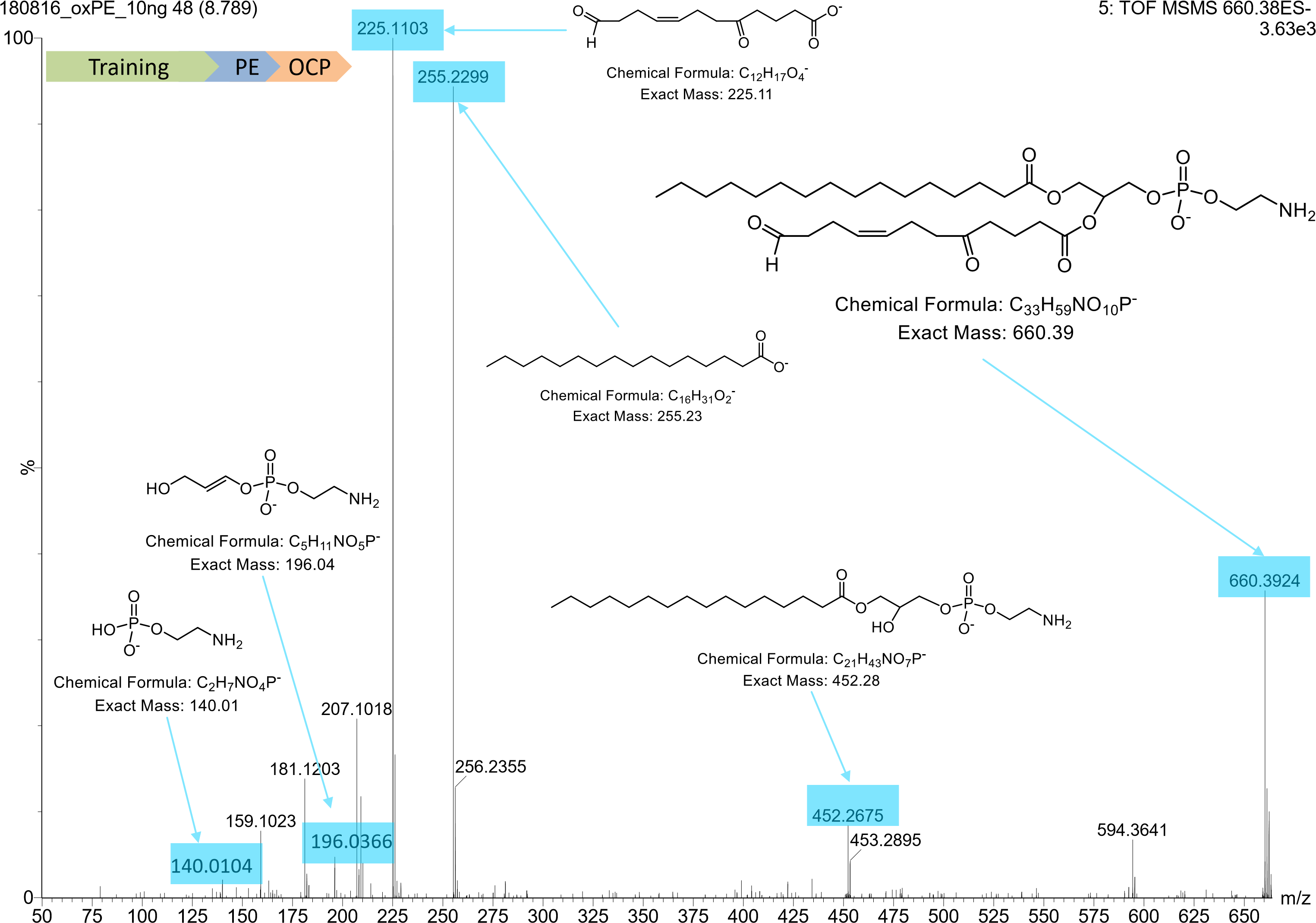


**Example 6:** LPPtiger identification report and corresponding original CID spectrum for ion at *m/z* 778.50^-^ (RT 9.3 min) identified as PE(16:0/22:6[6xDB,1xOH]) or 1-palmitoyl-2(hydroxy-docosahexaenoyl)-sn-glycero-3-phosphoethanolamine in *in vitro* oxidized PE samples.


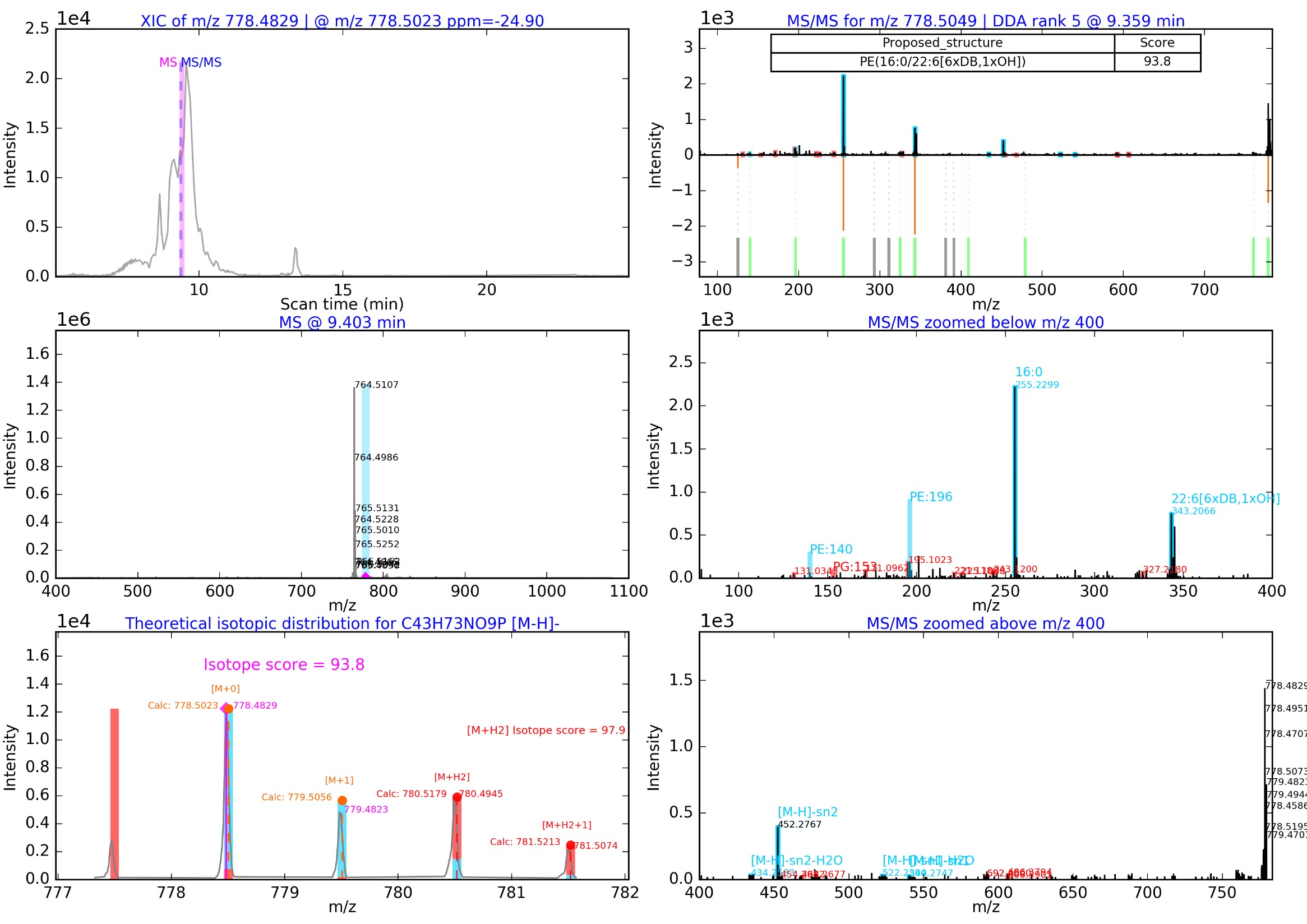


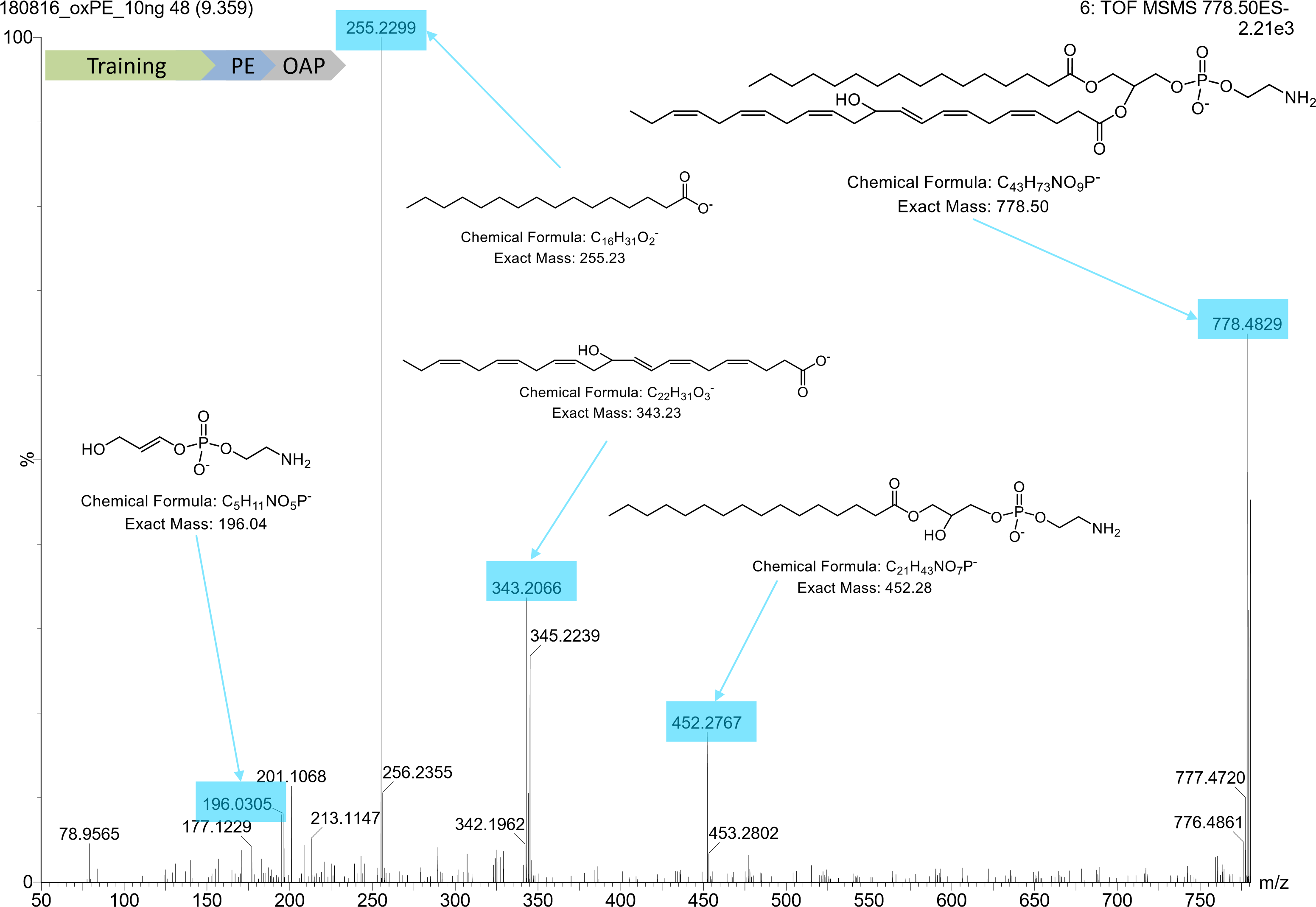


**Example 7:** LPPtiger identification report and corresponding original CID spectrum for ion at *m/z* 650.35^-^ (RT 13.8 min) identified as PS(16:0/9:0<CHO@C9>) or 1-palmitoyl-2-(9-oxononanoyl)-sn-glycero-3-phosphoserine in *in vitro* oxidized PS samples.


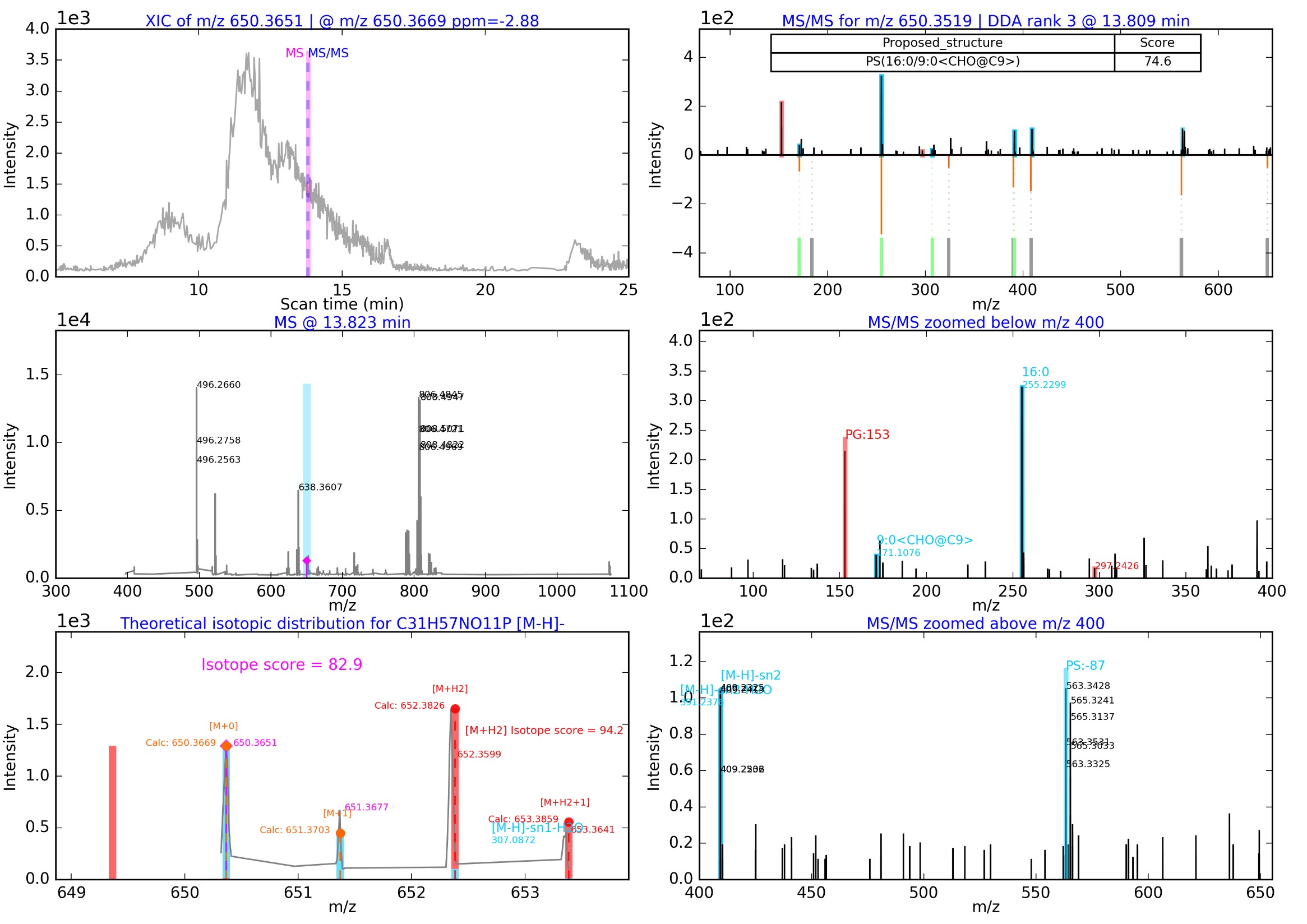


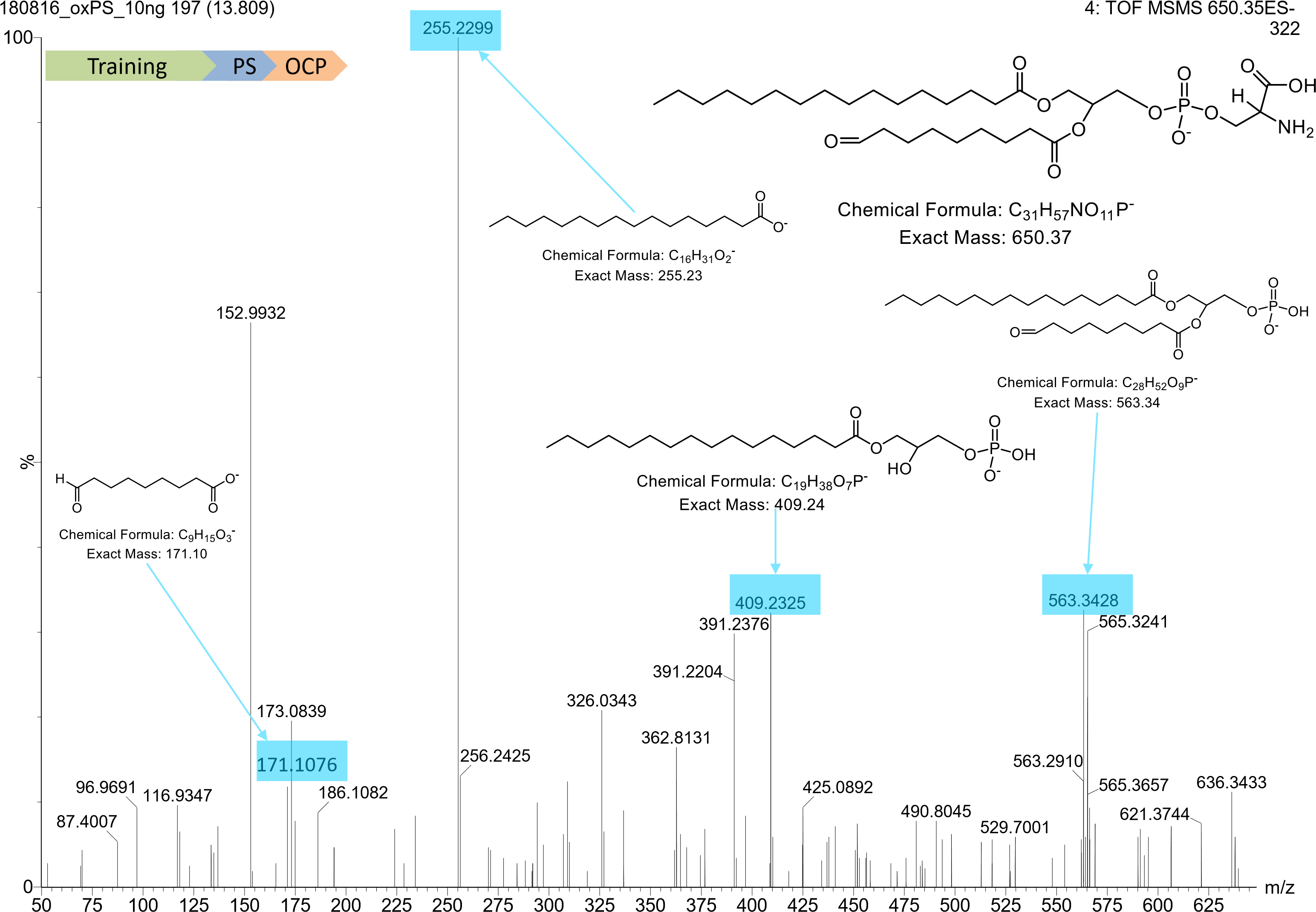


**Example 8:** LPPtiger identification report and corresponding original CID spectrum for ion at *m/z* 776.53^-^ (RT 16.7 min) identified as PS(16:0/18:1[1xDB,1xOH]) or 1-palmitoyl-2-

(hydroxy-octadecenoyl)-sn-glycero-3-phosphoserine in *in vitro* oxidized PS samples.


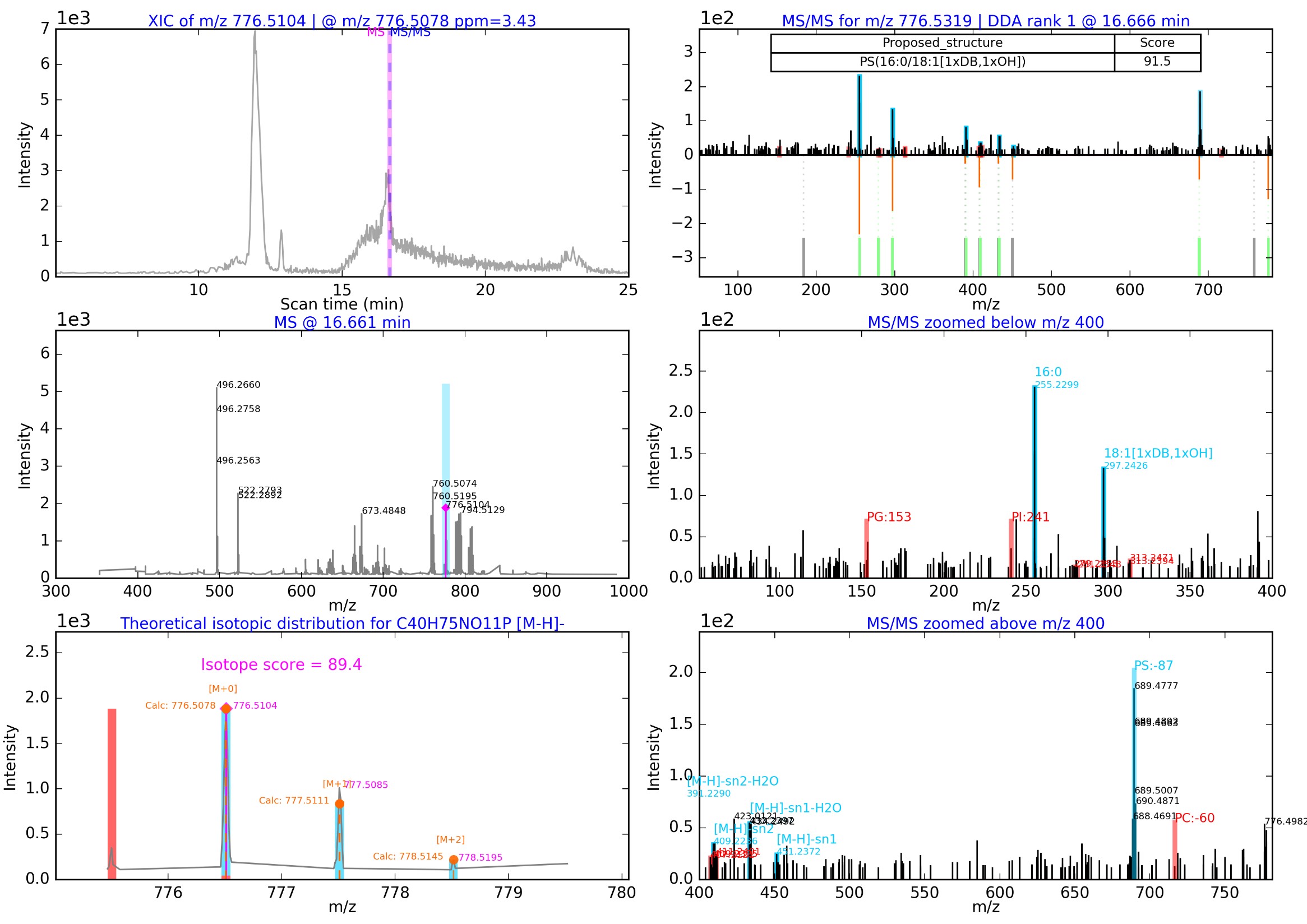


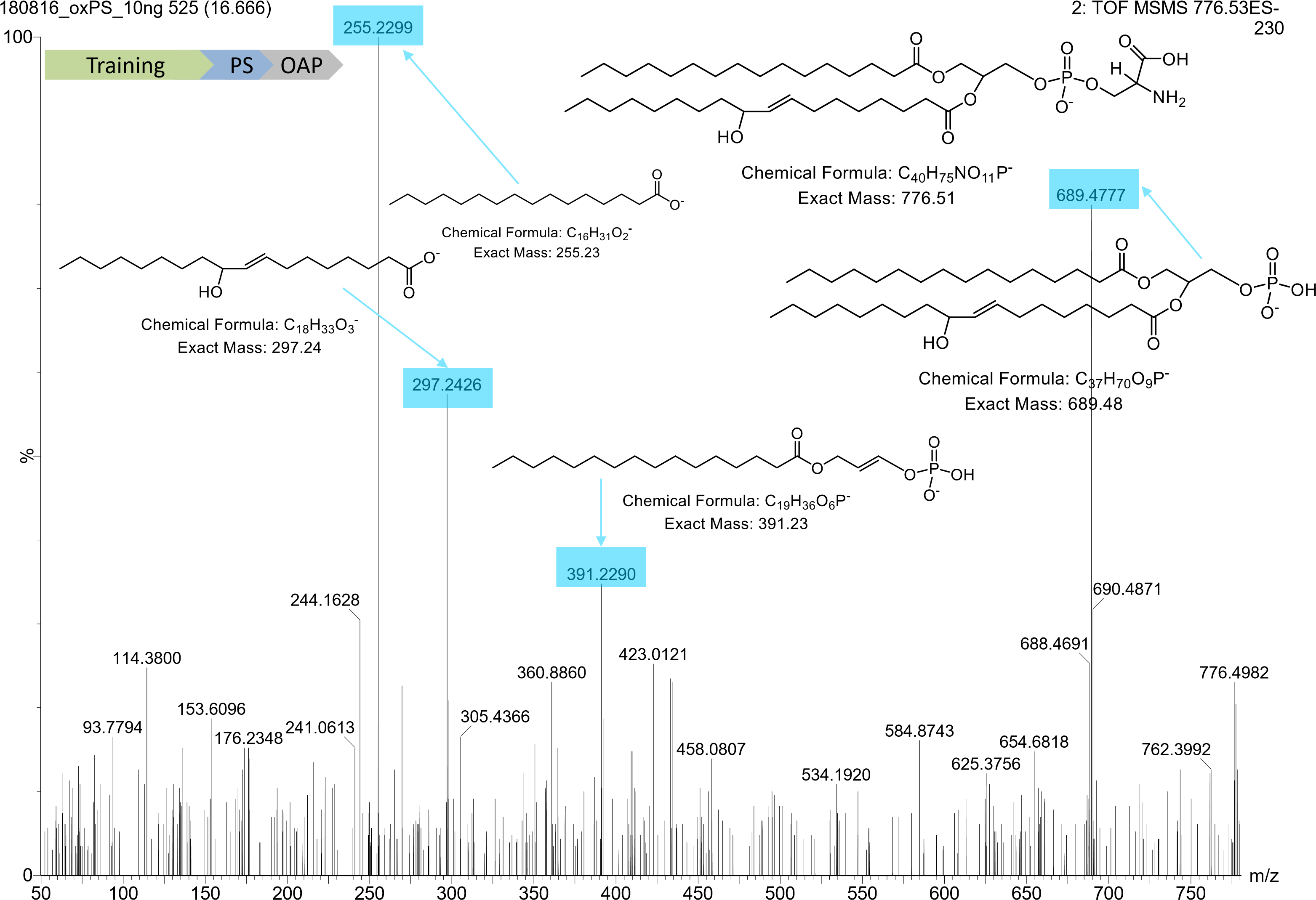


**Example 9:** LPPtiger identification report and corresponding original CID spectrum for ion at *m/z* 579.30^-^ (RT 10.2 min) identified as PA(16:0/9:0<COOH@C9>) or 1-palmitoyl-2-

(nonanedioyl)-sn-glycero-3-phosphate in *in vitro* oxidized PA samples.


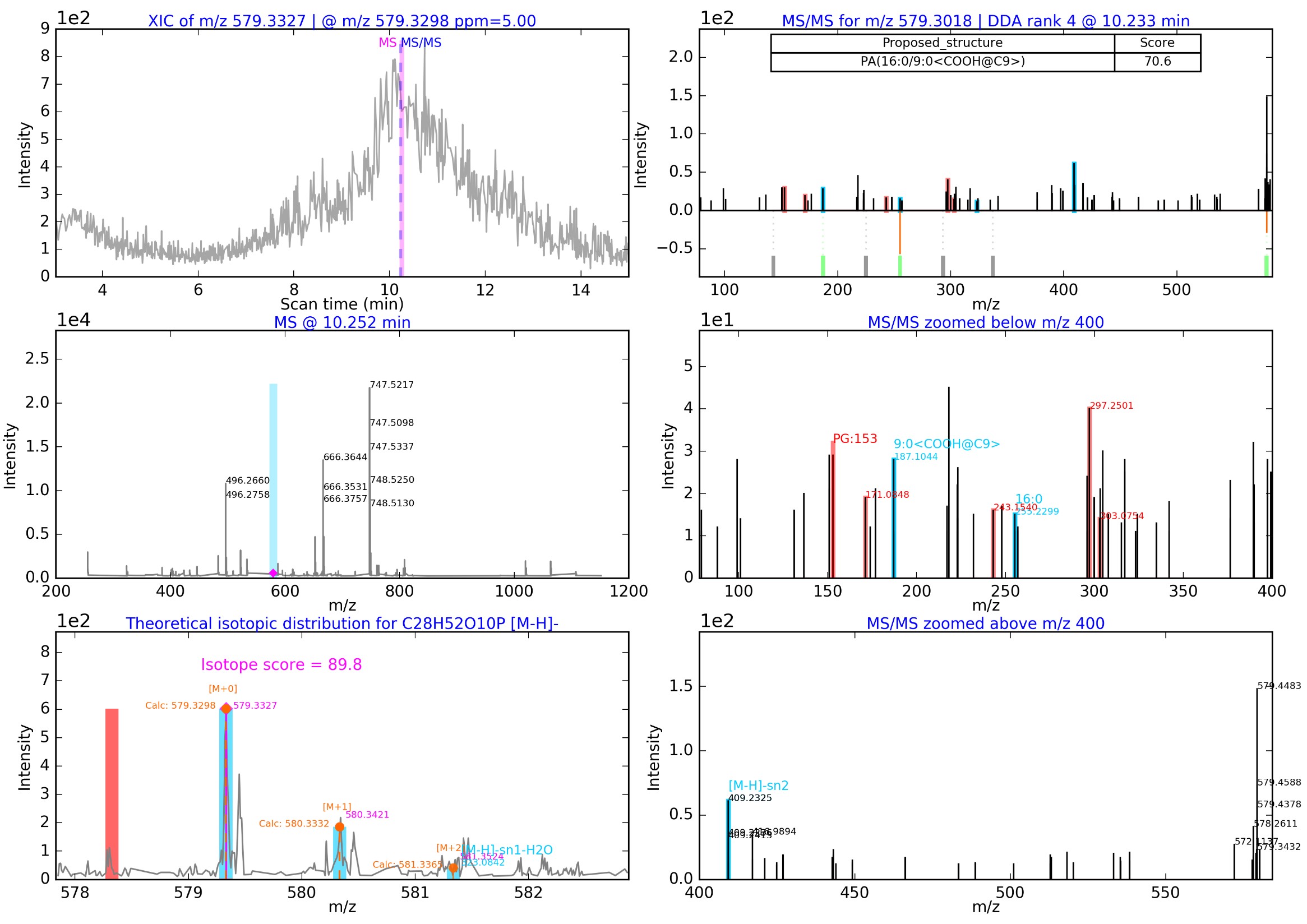


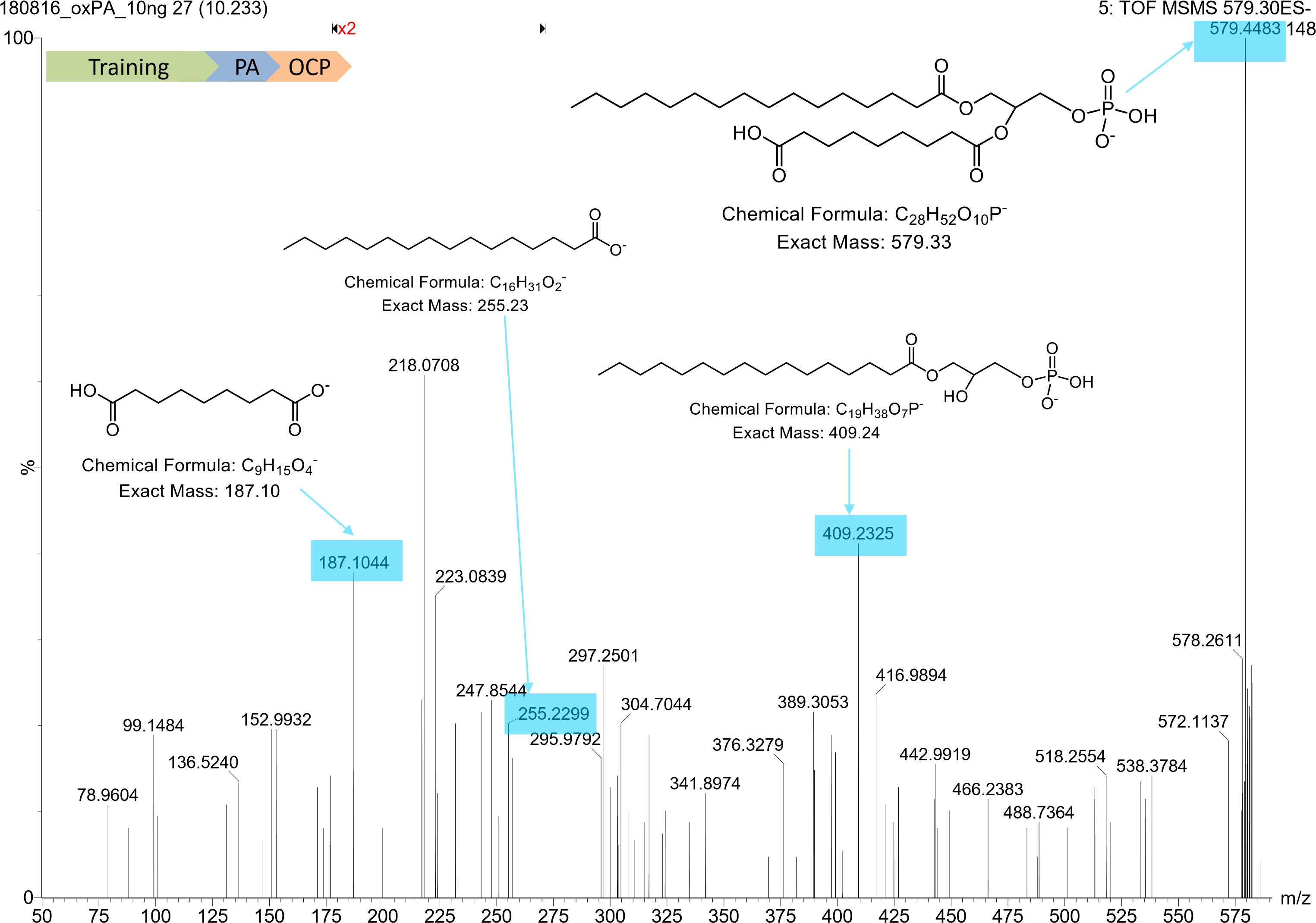


**Example 10:** LPPtiger identification report and corresponding original CID spectrum for ion at *m/z* 689.98^-^ (RT 19.7 min) identified as PA(16:0/18:1[1xDB,1xOH]) or 1-palmitoyl-2-

(hydroxy-octadecenoyl)-sn-glycero-3-phosphate in *in vitro* oxidized PA samples.


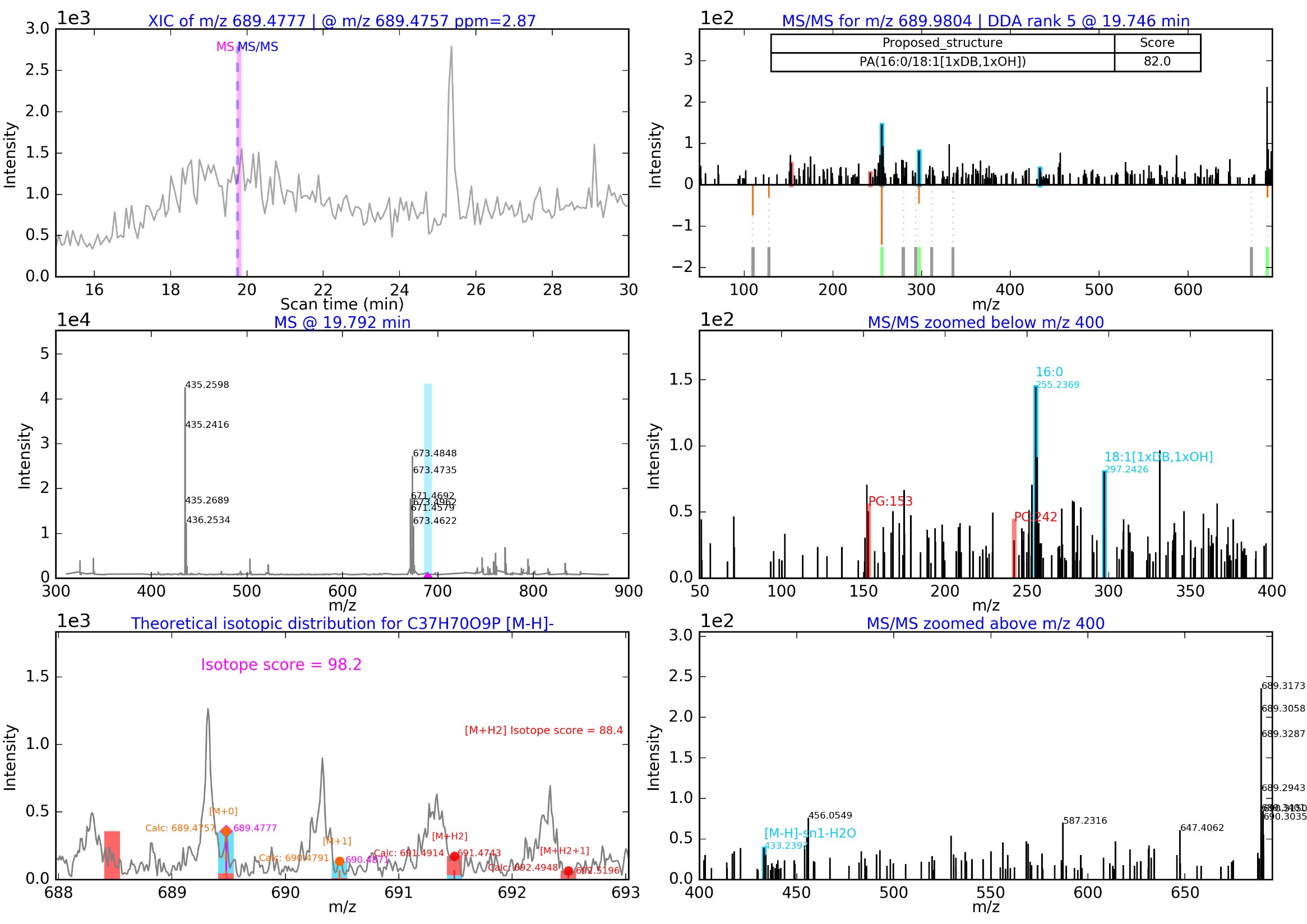


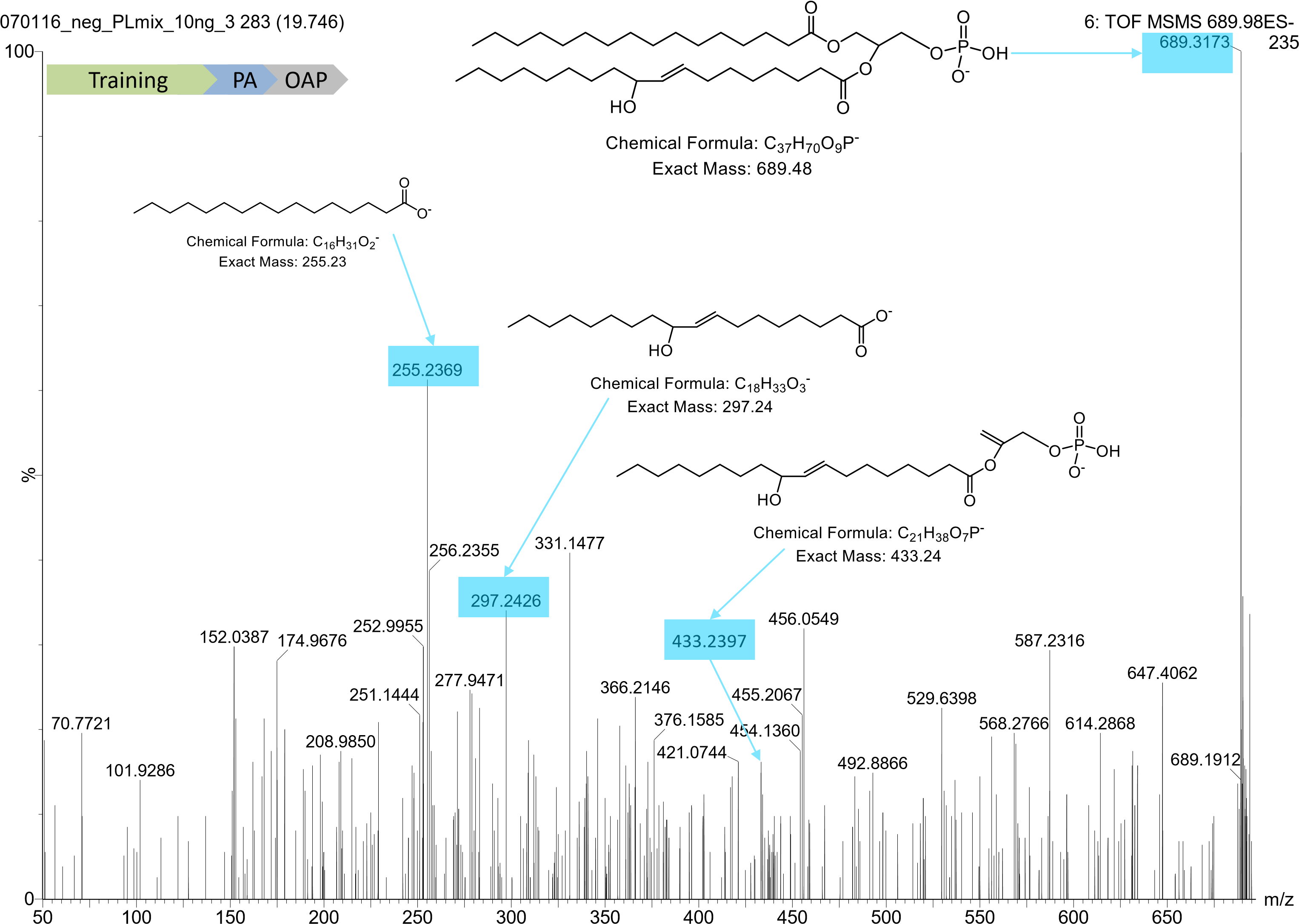


**Example 11:** LPPtiger identification report and corresponding original CID spectrum for ion at *m/z* 653.35^-^ (RT 14.5 min) identified as PG(16:0/9:0<COOH@C9>) or 1-palmitoyl-2-

(nonanedioyl)-sn-glycero-3-phosphoglycerol in *in vitro* oxidized PG samples.


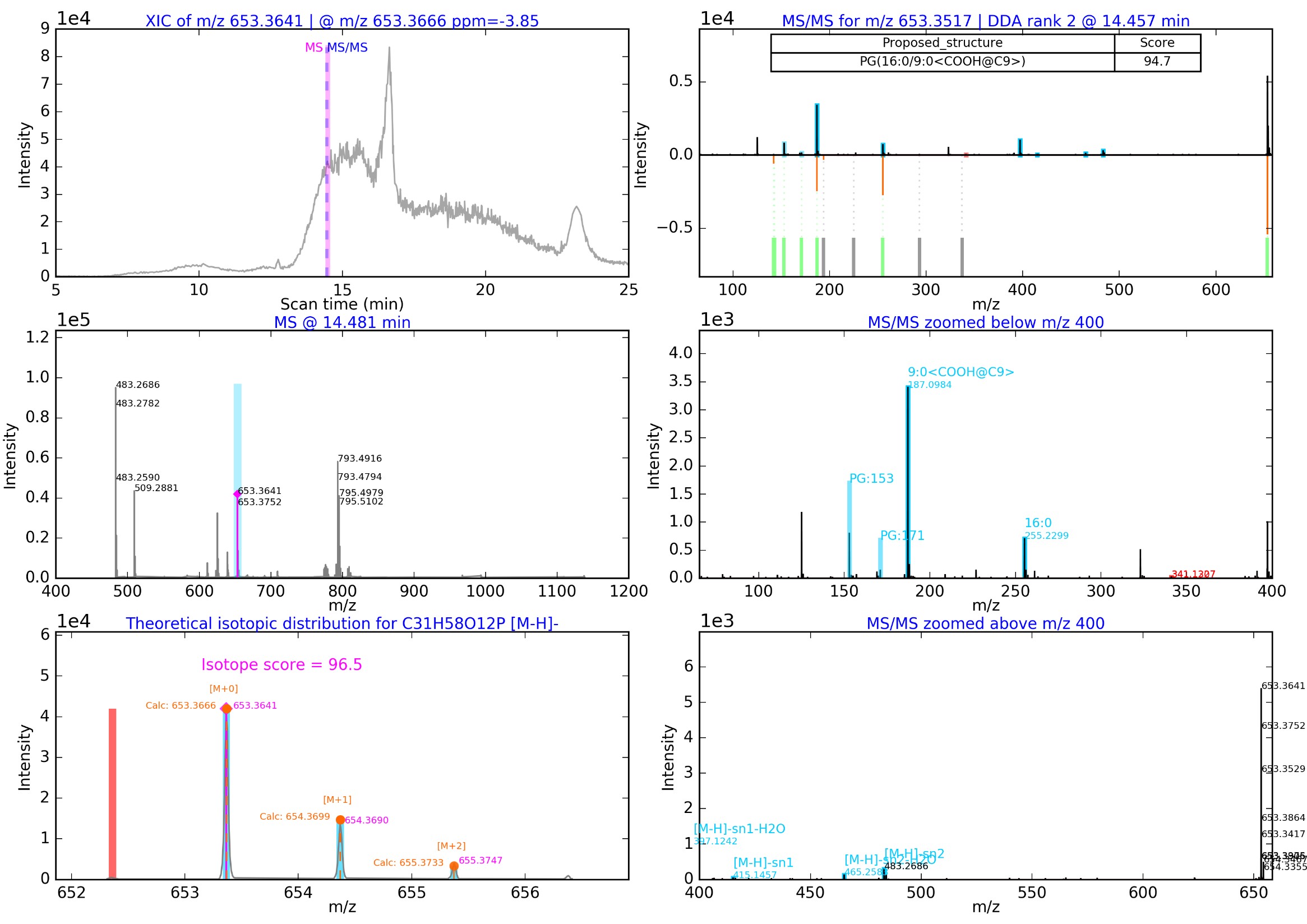


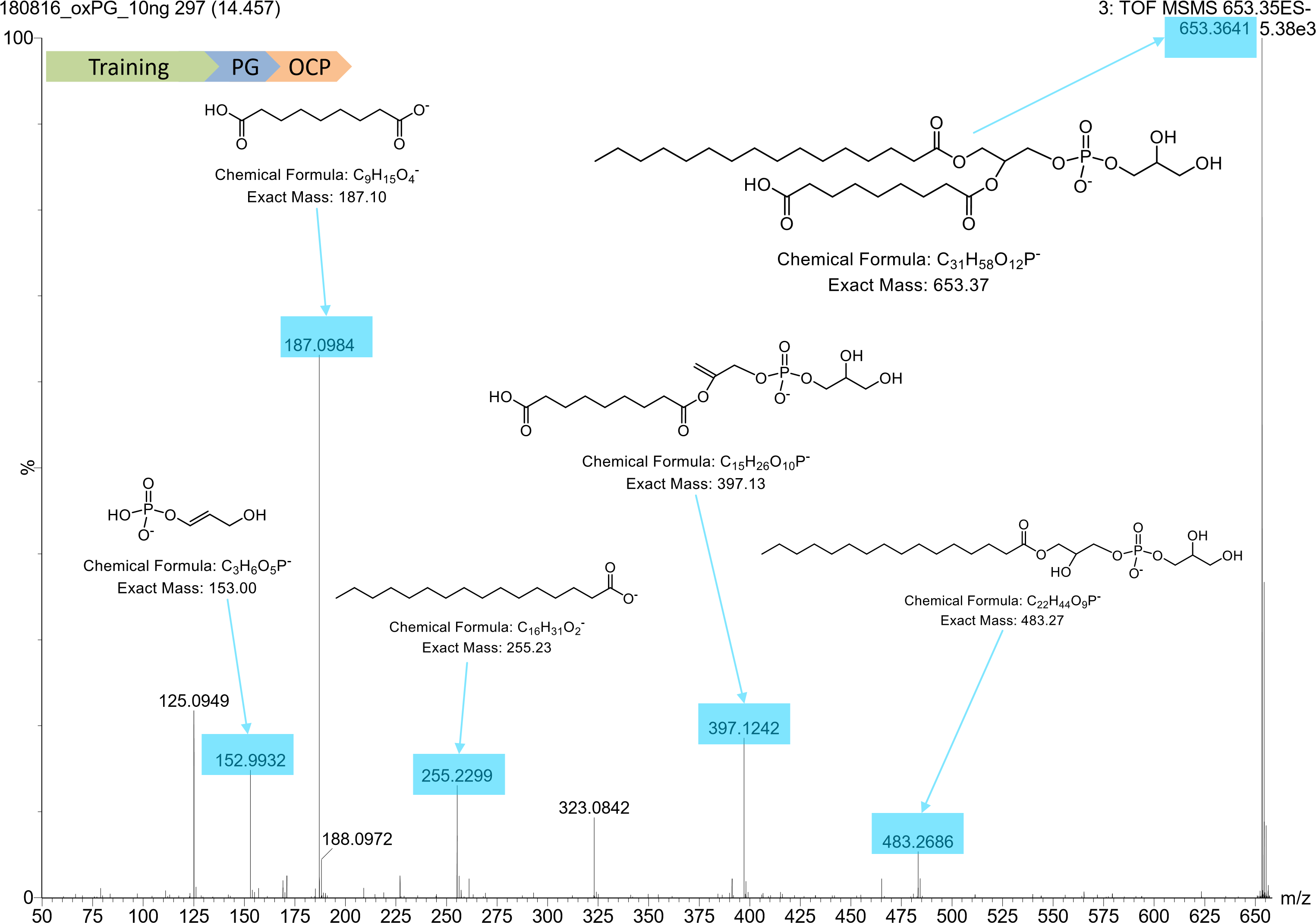


**Example 12:** LPPtiger identification report and corresponding original CID spectrum for ion at *m/z* 763.53^-^ (RT 20.5 min) identified as PG(16:0/18:1[1xDB,1xOH]) or 1-palmitoyl-2-

(hydroxy-octadecenoyl)-sn-glycero-3-phosphoglycerol in *in vitro* oxidized PG samples.


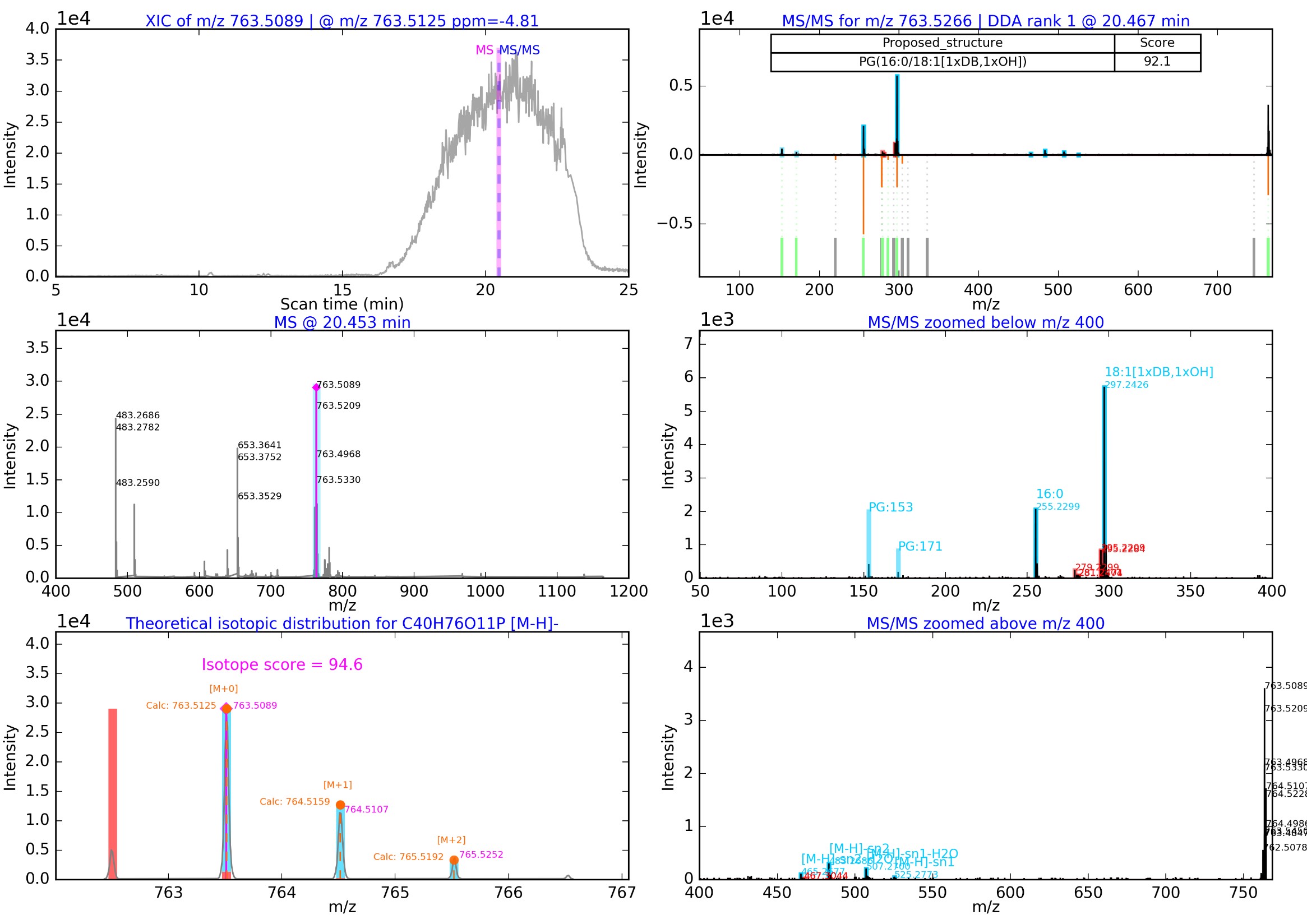


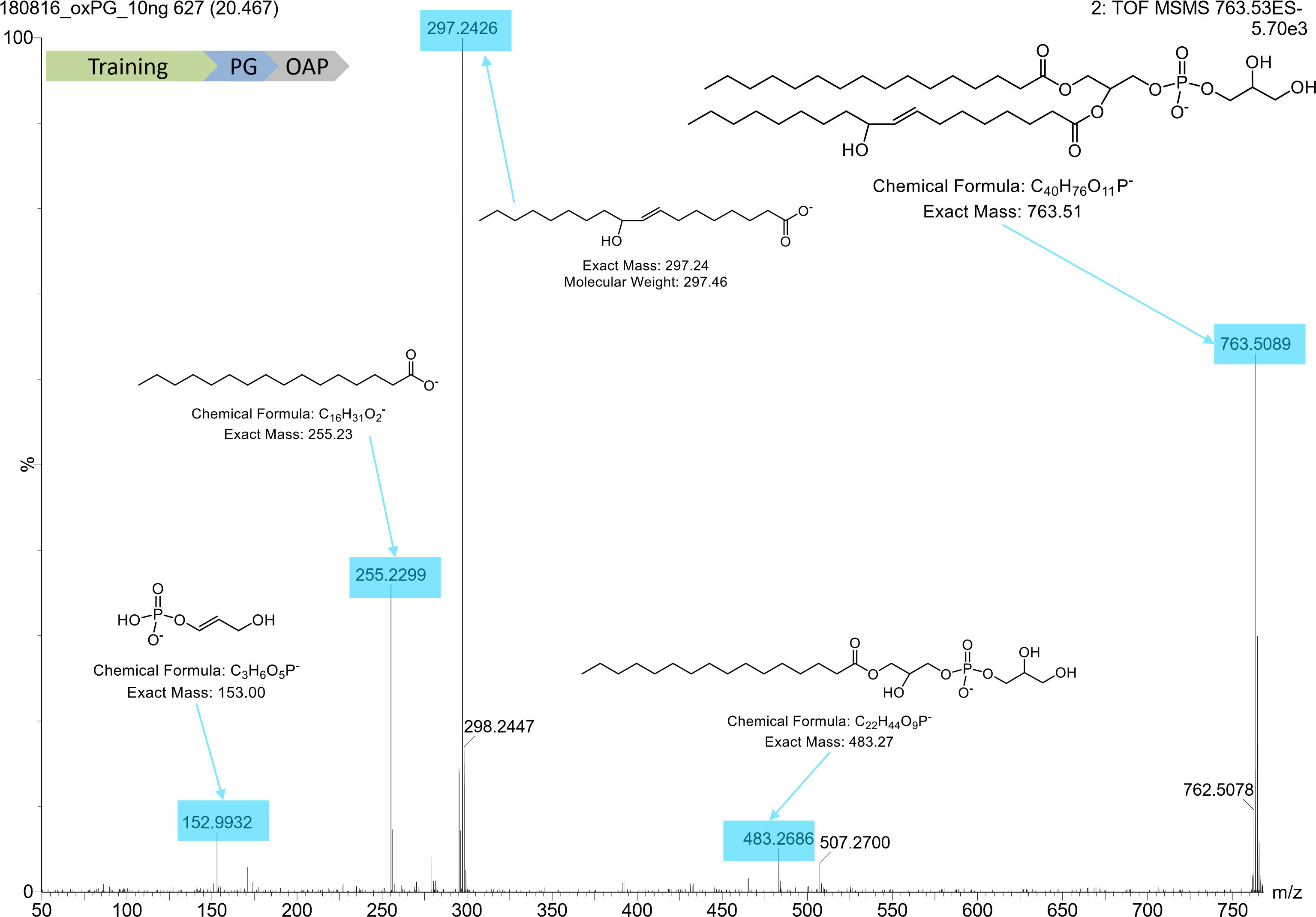


2. LPPtiger identification examples from SIN-1 treated cardiomyocytes lipidomes

**Example 13:** LPPtiger identification report and corresponding original CID spectrum for ion at *m/z* 694.77^-^ (RT 10.2 min) identified as PC(16:0/9:0<CHO@C9>) or 1-palmitoyl-2-(9-oxononanoyl)-*sn*-glycerol-3-phosphocholine in cardiomyocytes lipid extracts.


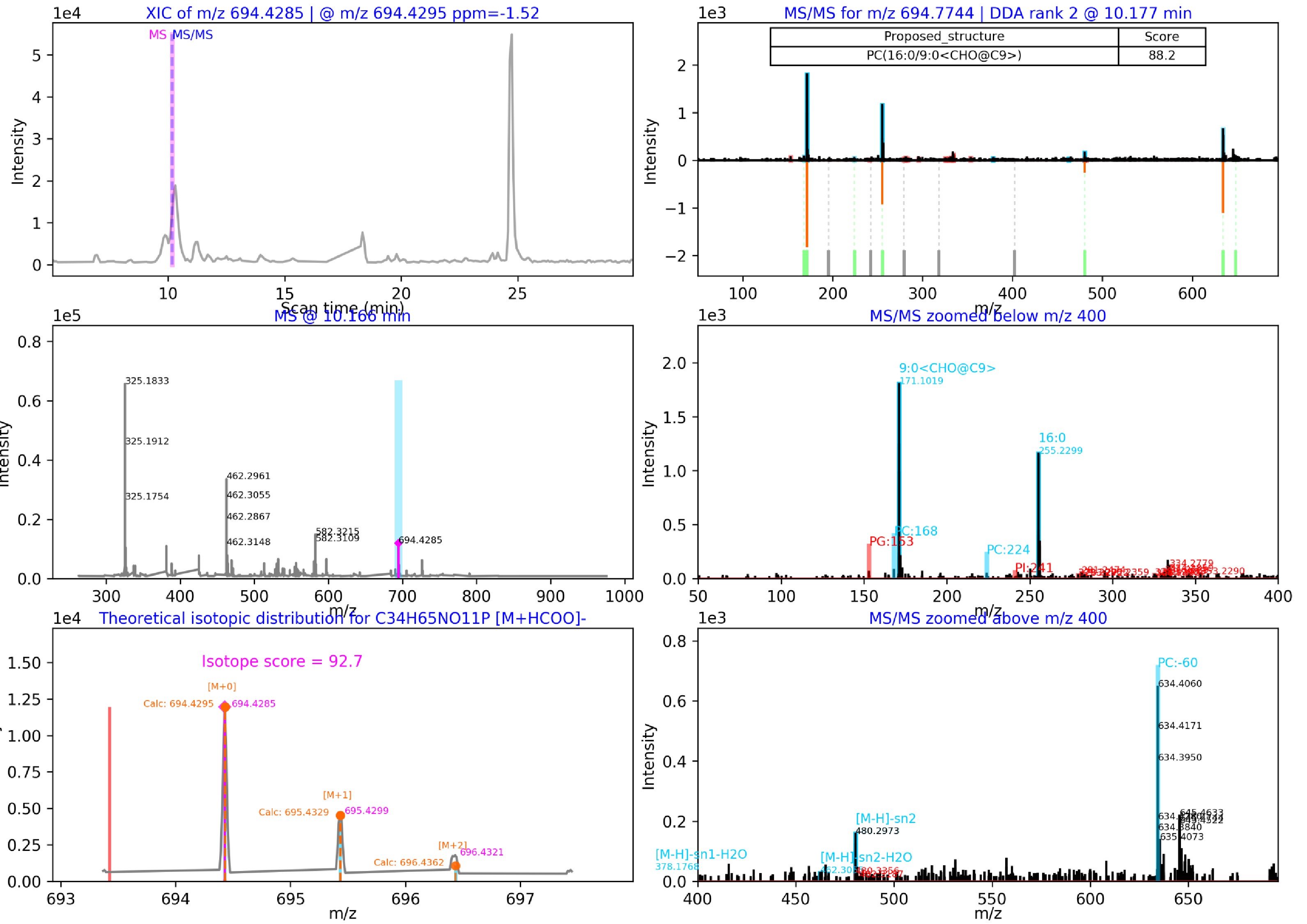


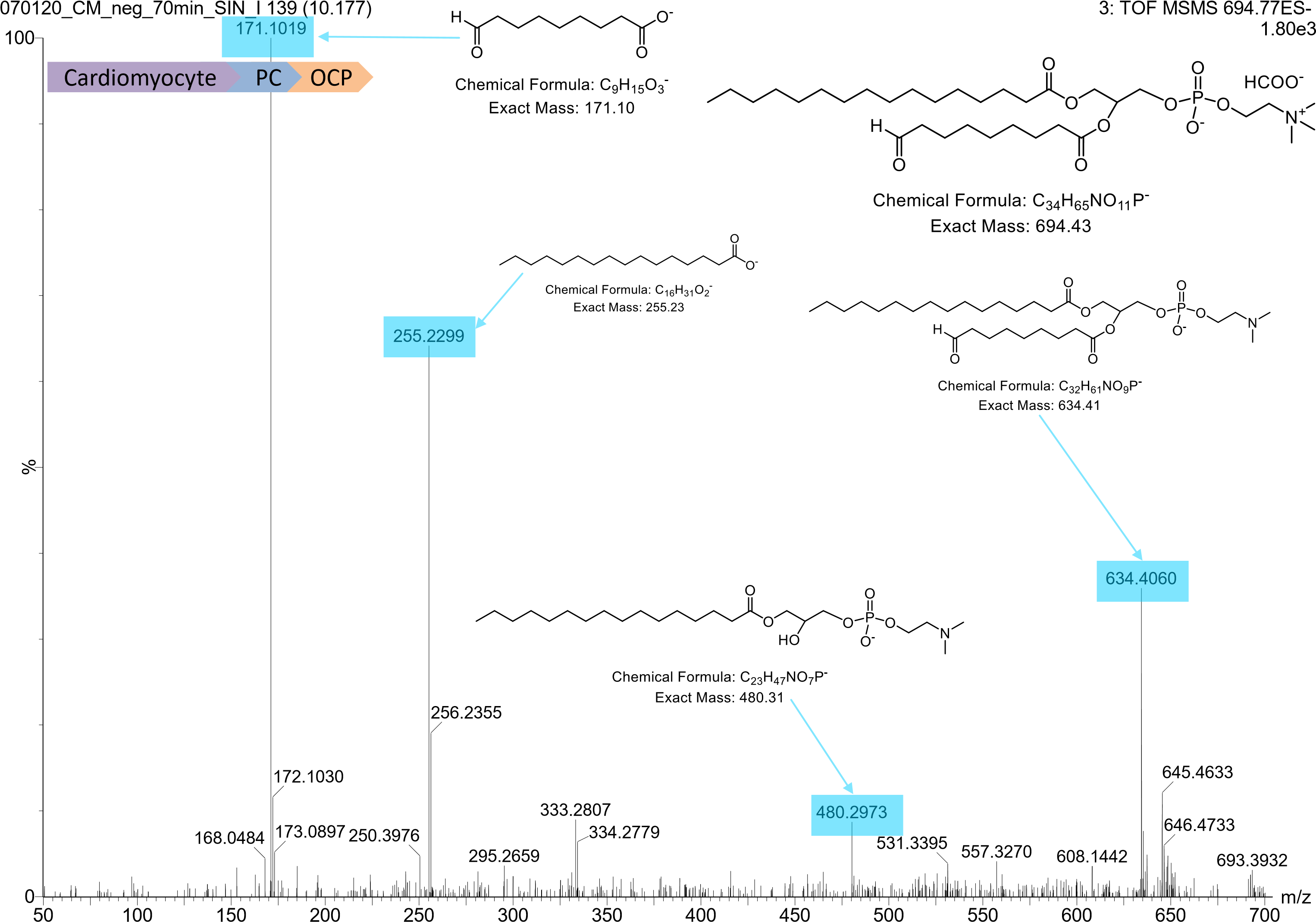


**Example 14:** LPPtiger identification report and corresponding original CID spectrum for ion at *m/z* 818.96^-^ (RT 19.1 min) identified as PC(16:0/18:1[1xDB,1xKETO]) or 1-palmitoyl-2-

(oxo-octadecenoyl)-sn-glycero-3-phosphocholine in cardiomyocytes lipid extracts.


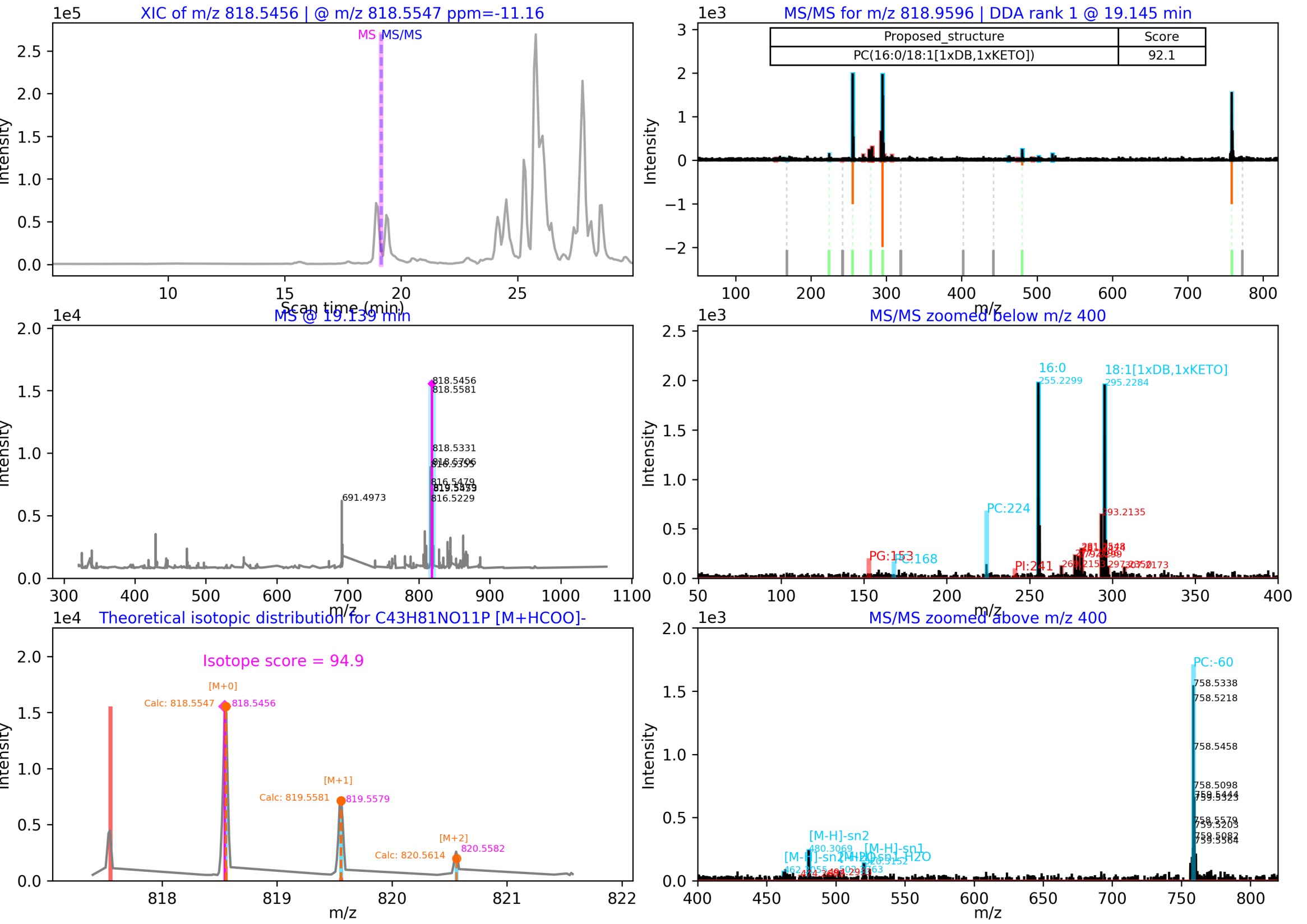


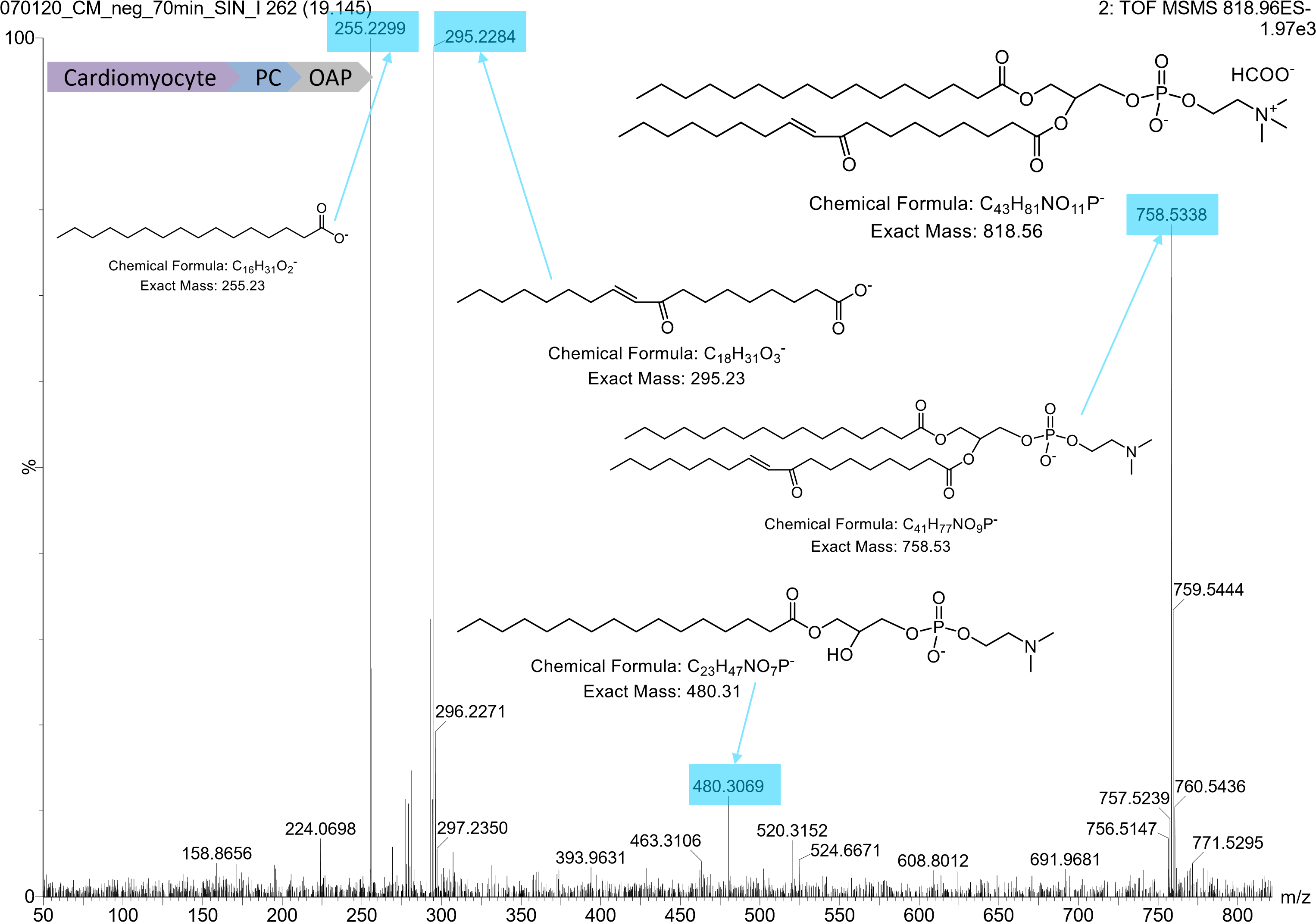


**Example 15:** LPPtiger identification report and corresponding original CID spectrum for ion at *m/z* 578.63^-^ (RT 12.0 min) identified as PE(18:0/5:0<CHO@C5>) or 1-stearoyl-2-(5-oxopentanoyl)-sn-glycero-3-phosphoethanolamine in cardiomyocytes lipid extracts.


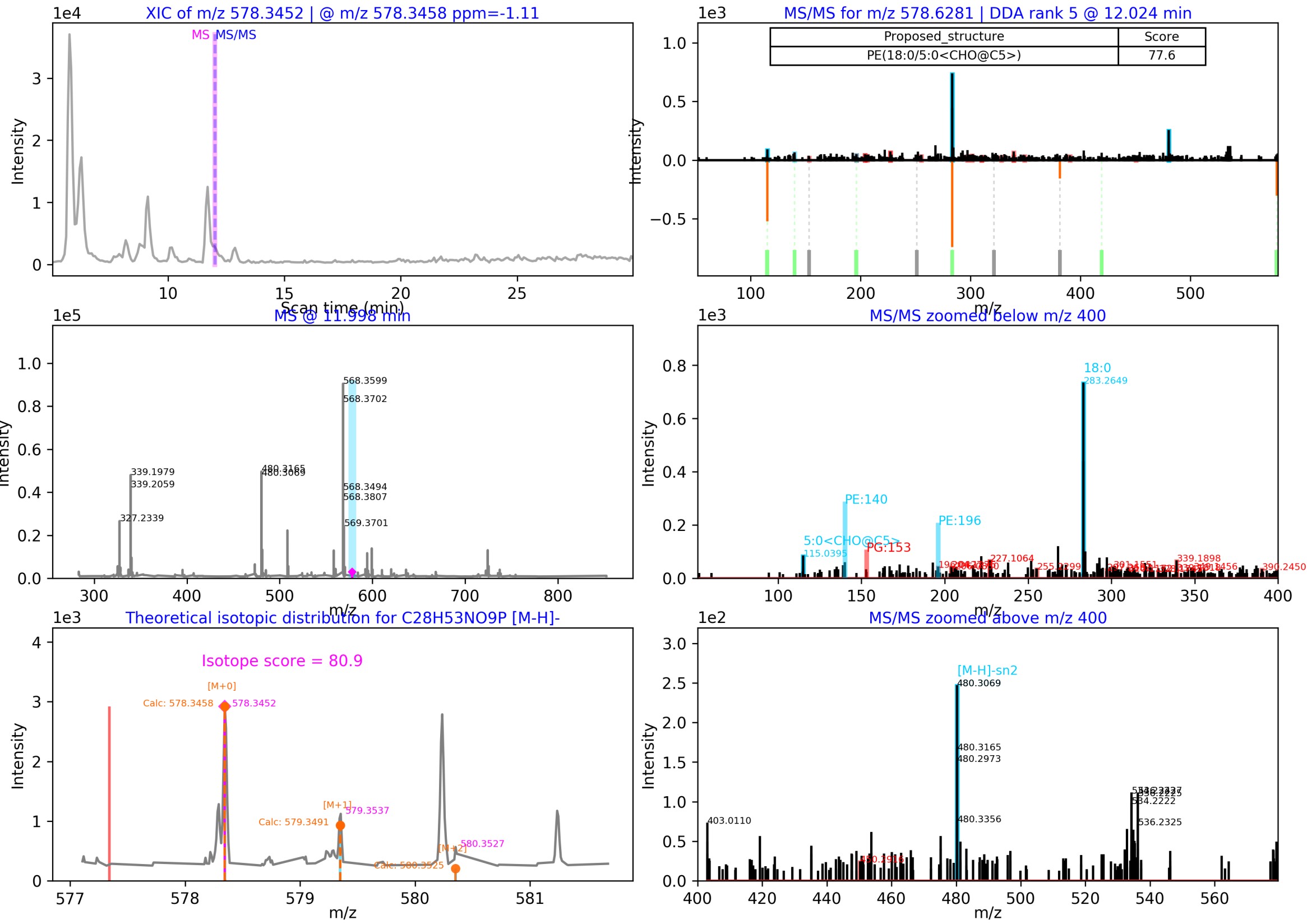


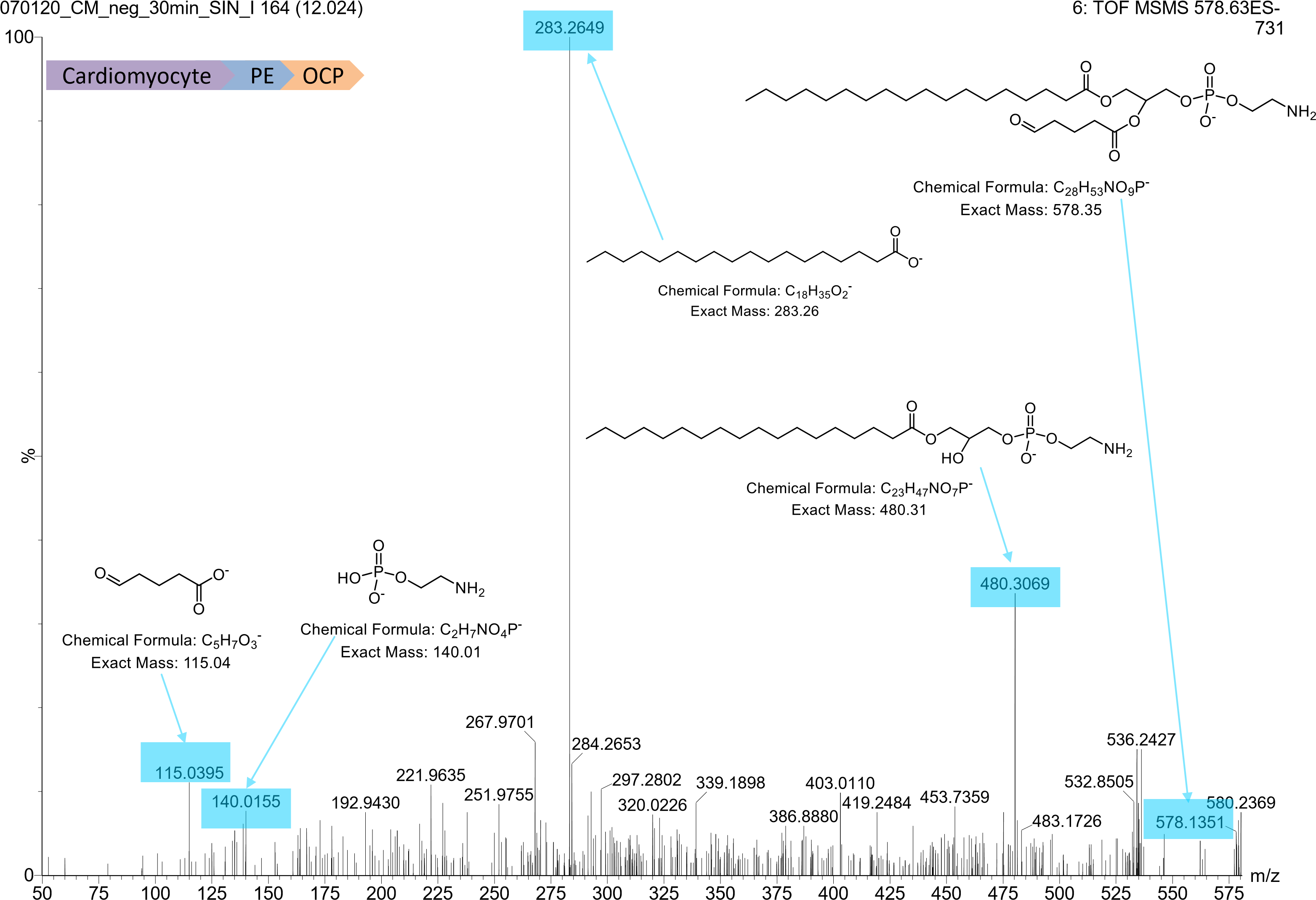


**Example 16:** LPPtiger identification report and corresponding original CID spectrum for ion at *m/z* 606.67^-^ (RT 12.8 min) identified as PE(18:0/7:0<CHO@C7>) or 1-stearoyl-2-(7-oxoheptanoyl)-sn-glycero-phosphoethanolamine in cardiomyocytes lipid extracts.


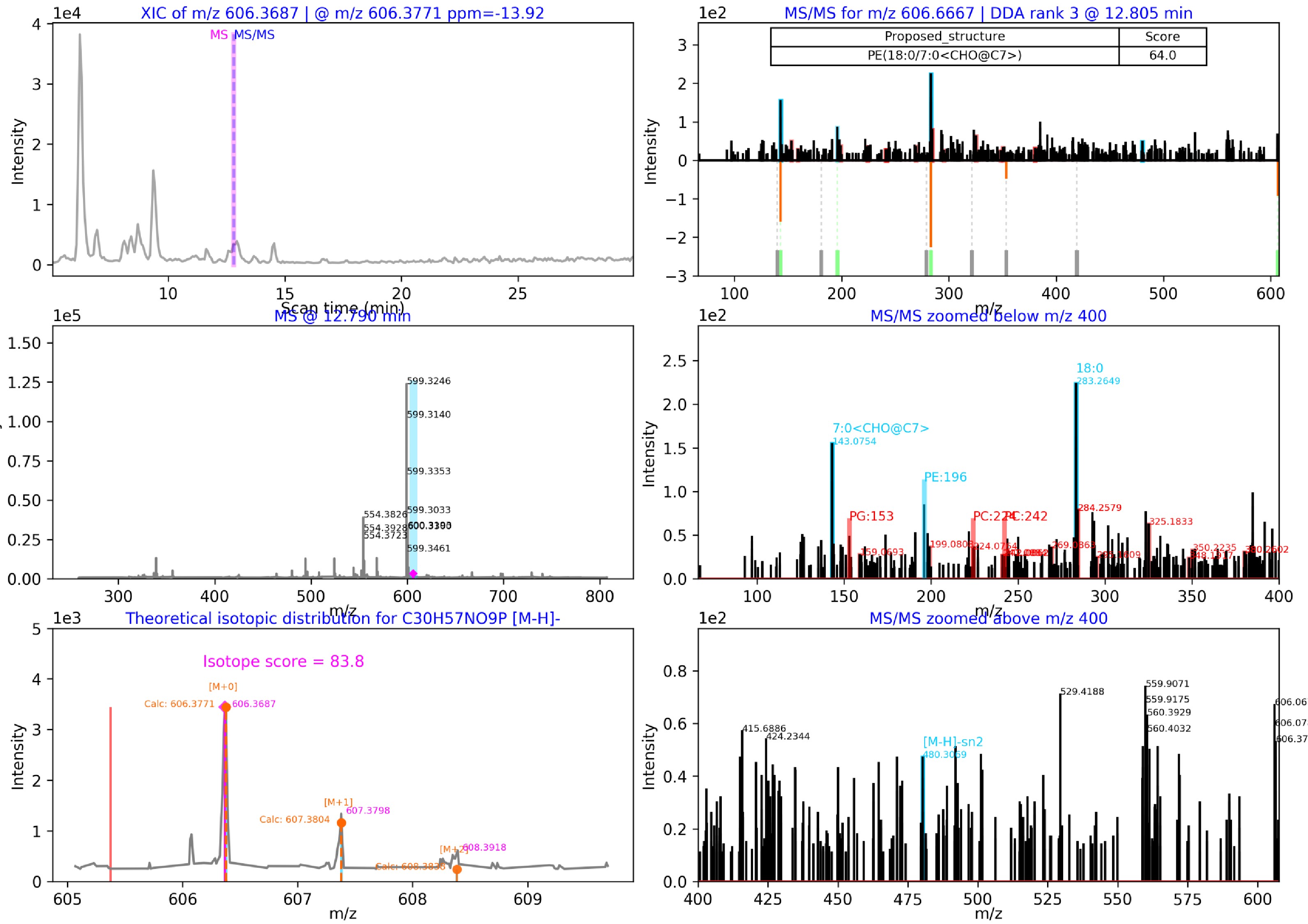


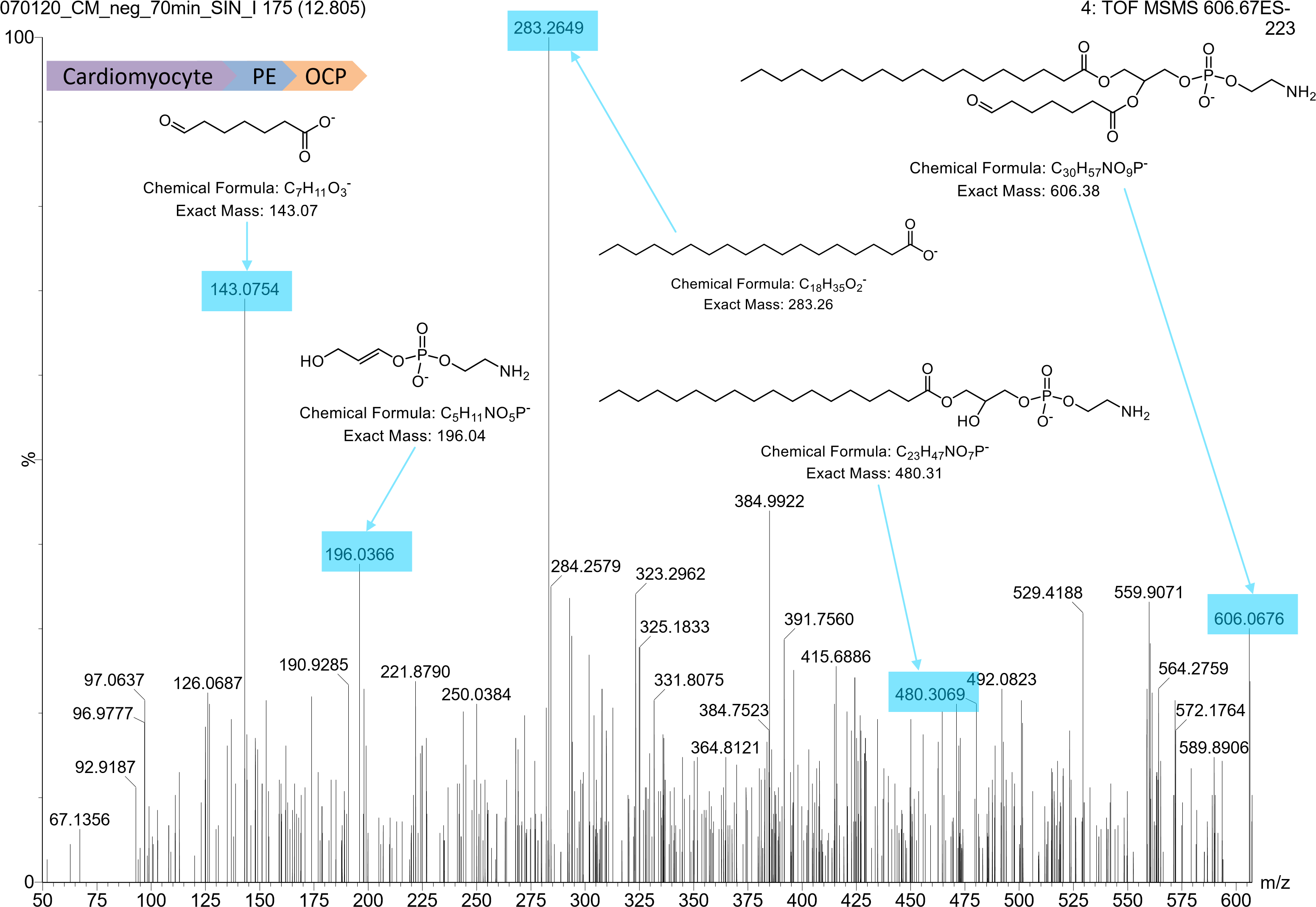


**Example 17:** LPPtiger identification report and corresponding original CID spectrum for ion at *m/z* 622.64^-^ (RT 12.5 min) identified as PS(18:0/5:0<CHO@C5>) or 1-stearoyl-2-(5-oxopentanoyl)-sn-glycero-3-phosphoserine in cardiomyocytes lipid extracts.


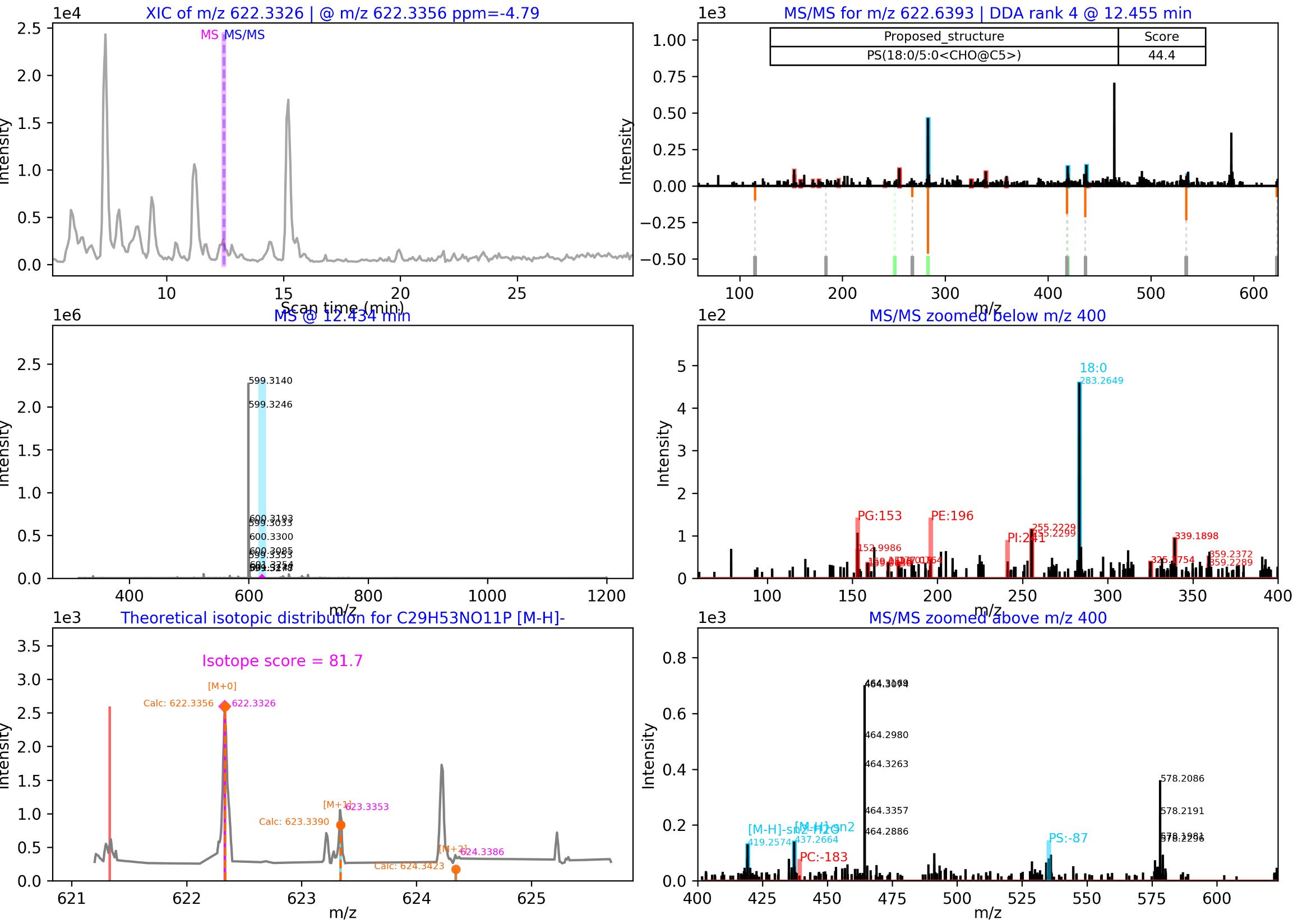


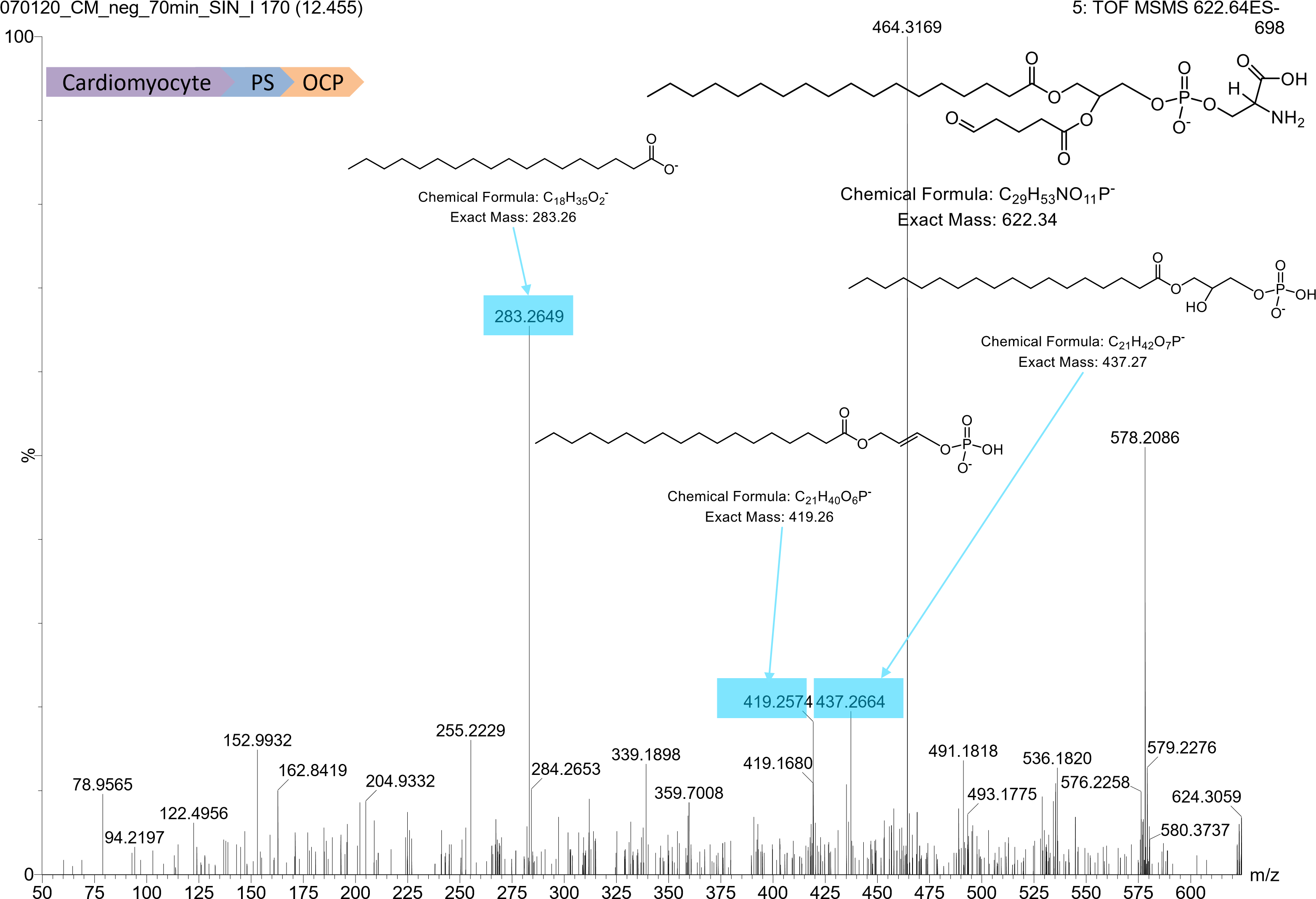


**Example 18:** LPPtiger identification report and corresponding original CID spectrum for ion at *m/z* 886.09^-^ (RT 28.7 min) identified as PS(18:0/22:4[4xDB,3xOH]) or 1-stearoyl-2(dihydroxy-docosatetraenoyl)-sn-glycero-3-phosphoserine in cardiomyocytes lipid extracts.


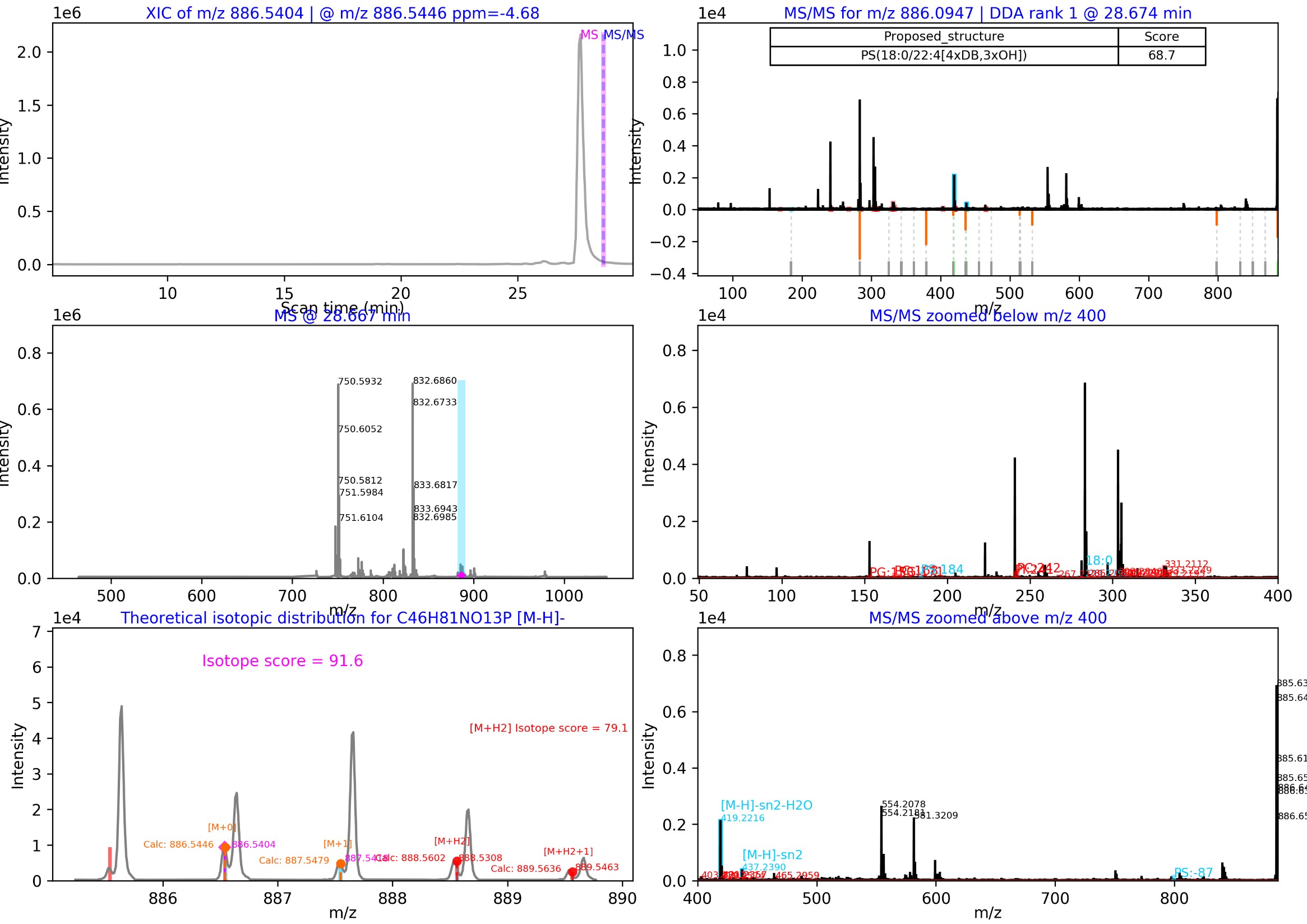


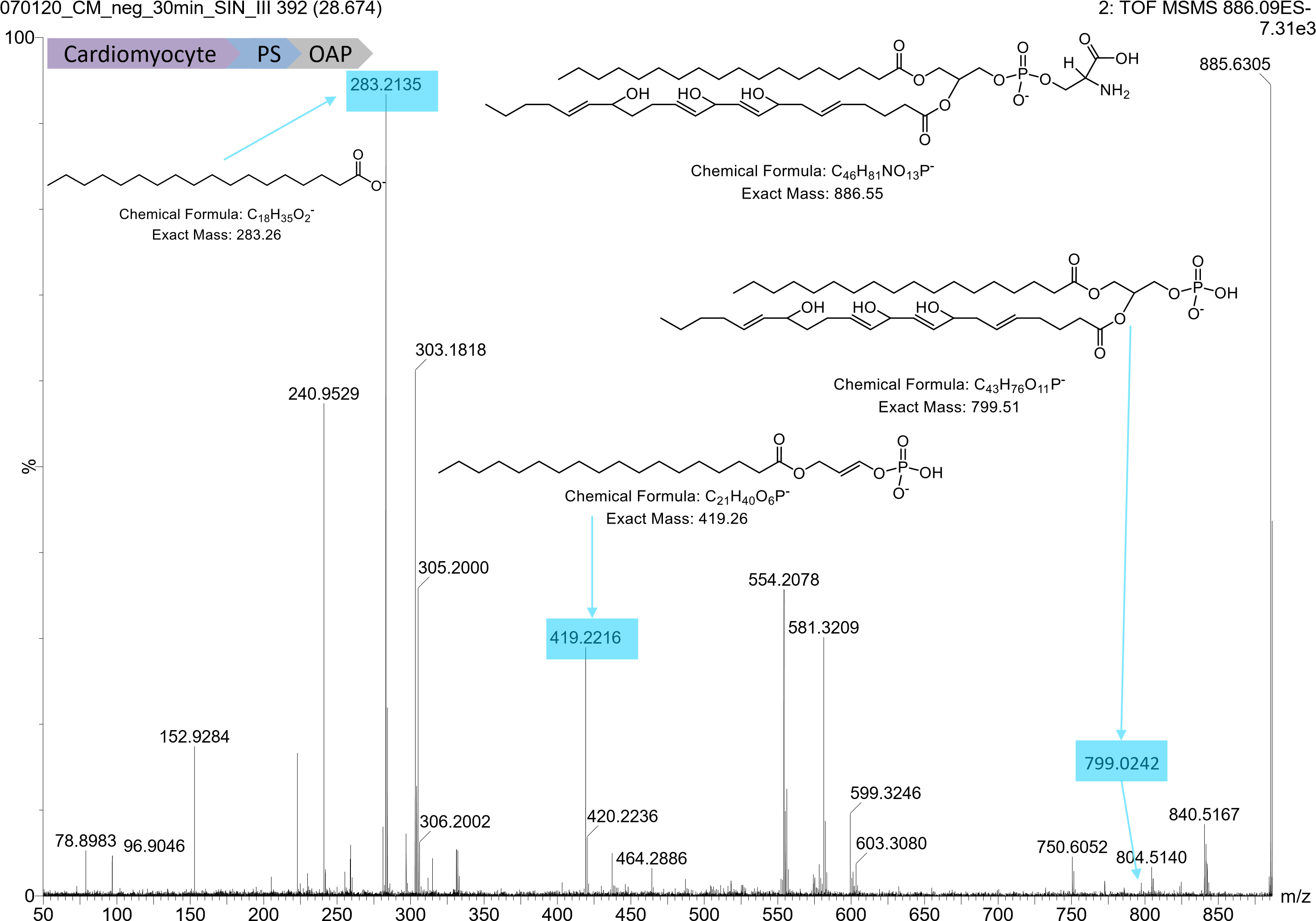


**Example 19:** LPPtiger identification report and corresponding original CID spectrum for ion at *m/z* 777.84^-^ (RT 7.8 min) identified as PA(18:1/J3-dihomo-IsoP-22:3) or 1-stearoyl-2-(J3dihomo-isoprostane)-sn-glycero-3-phosphate in cardiomyocytes lipid extracts.


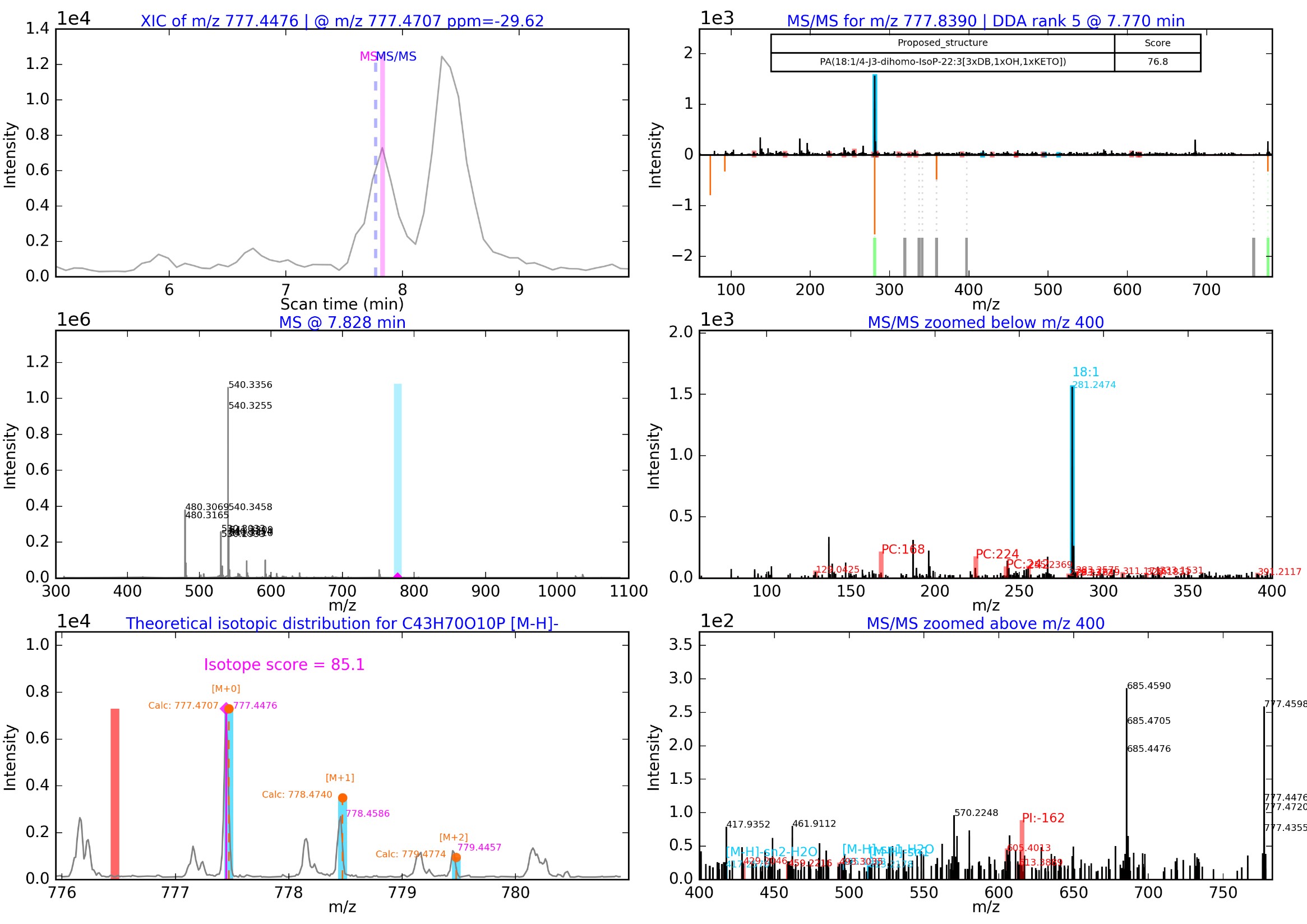


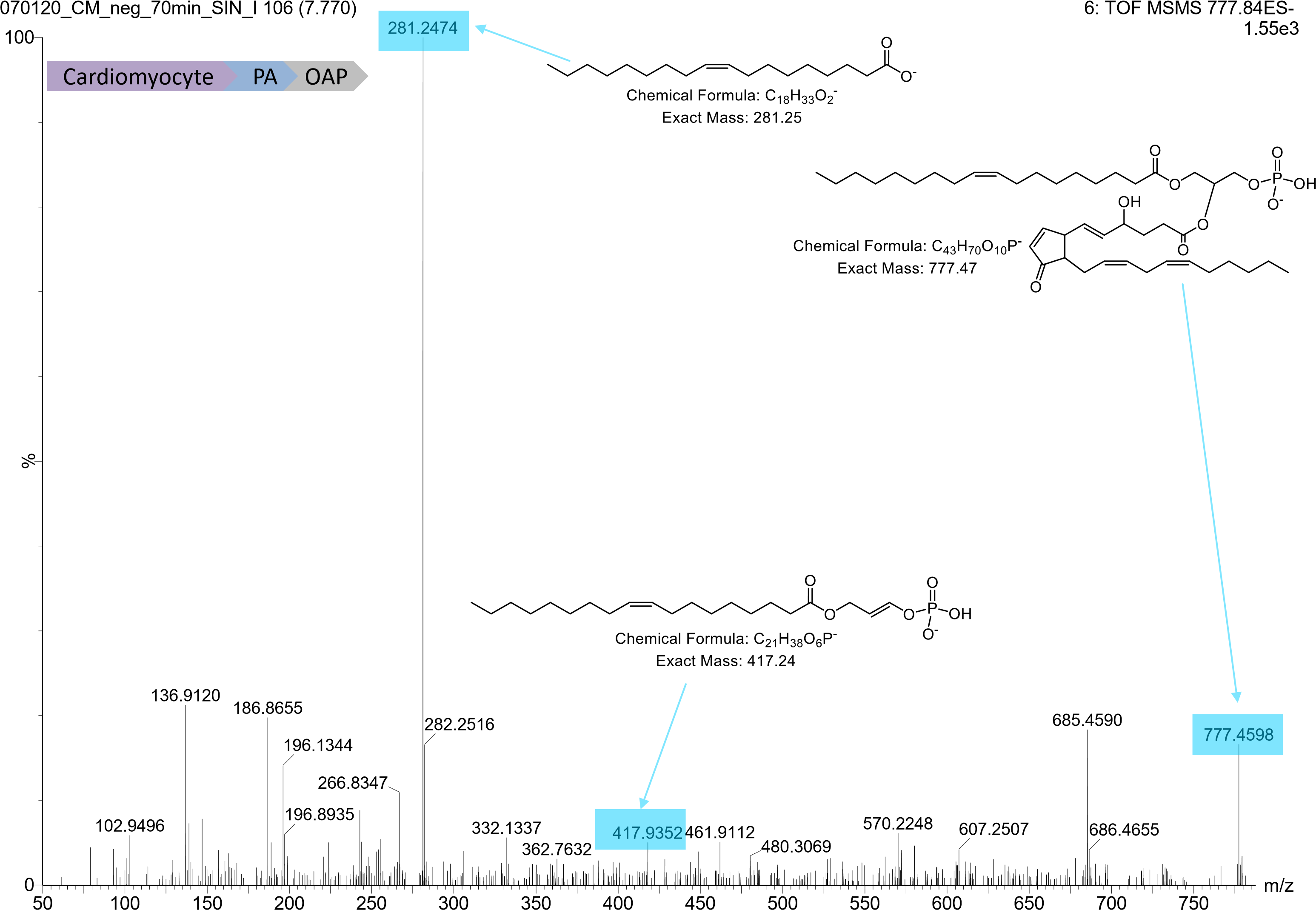


**Example 20:** LPPtiger identification report and corresponding original CID spectrum for ion at *m/z* 867.96^-^ (RT 24.2 min) identified as PG(22:5/F1-PhytoP-18:1) or 1-docosapentaenoyl-

2-(F1-phytoprostane)-sn-glycero-3- phosphoglycerol in cardiomyocytes lipid extracts.


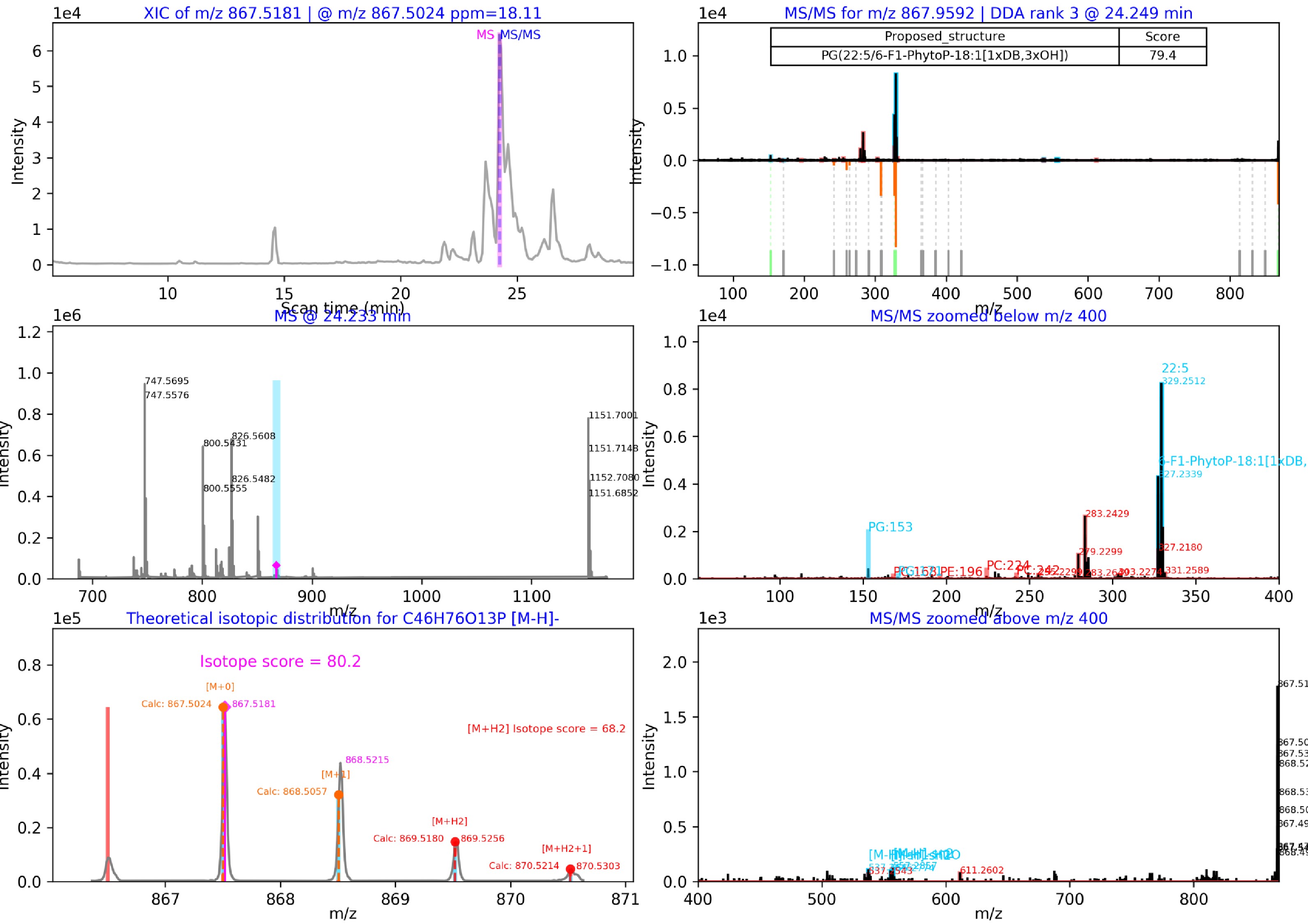


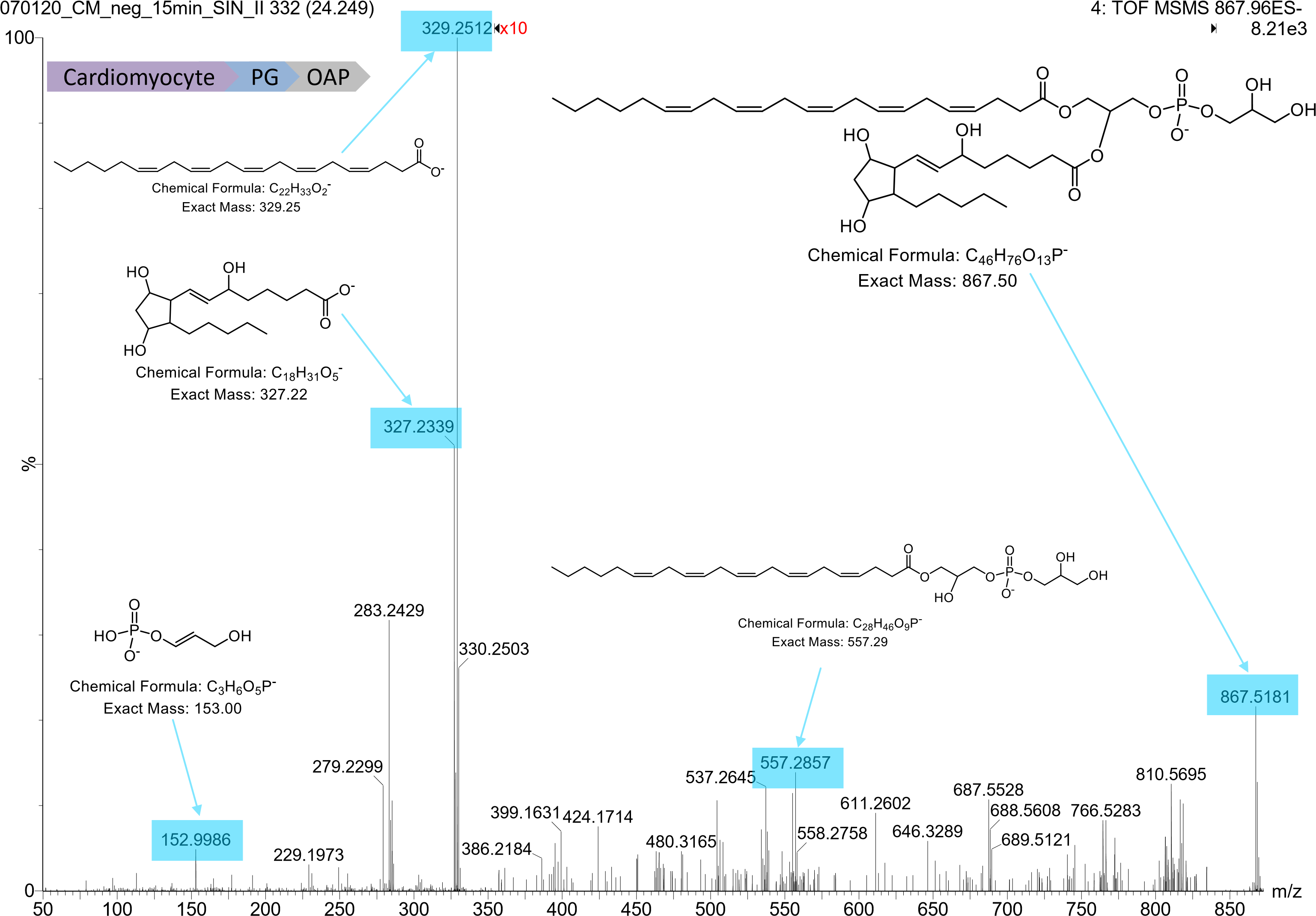

Supplement: Supplementary file 20 — Supplementary File 12 [file 41598_2017_15363_MOESM20_ESM.doc]
